# Supplementary material for: Perceived socio-economic impacts of the marbled crayfish invasion in Madagascar
Source: PLoS One. 2020 Apr 15;15(4):e0231773. doi: 10.1371/journal.pone.0231773 (PMC7159205; doi:10.1371/journal.pone.0231773)
Supplement: S1 Data — (DOCX) [file pone.0231773.s001.docx]

Supporting Information

**Socio-economic impacts of the marbled crayfish invasion in Madagascar**

Andriantsoa et al.


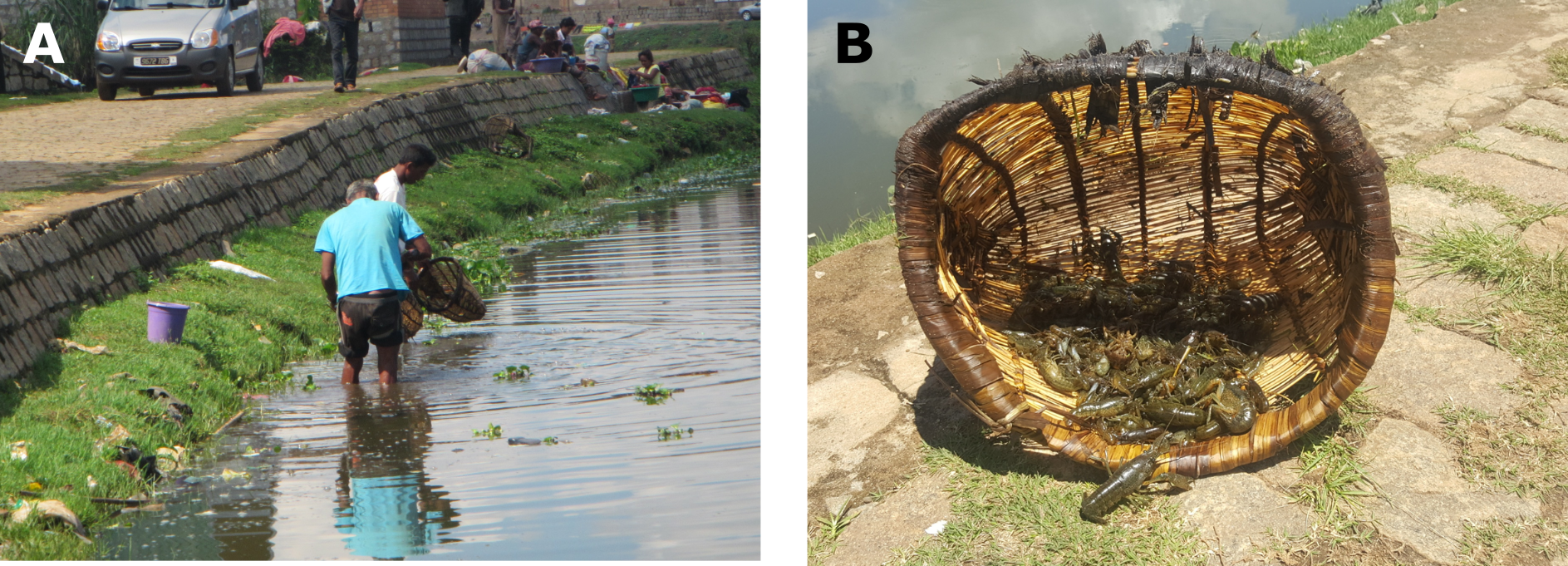


S1 Figure. Marbled crayfish collection. (A). Fishing in a lake with the traditional fishing tool: “*Tandroho*”. (B) The traditional fishing tool: “*Tandroho*” with marbled crayfish.


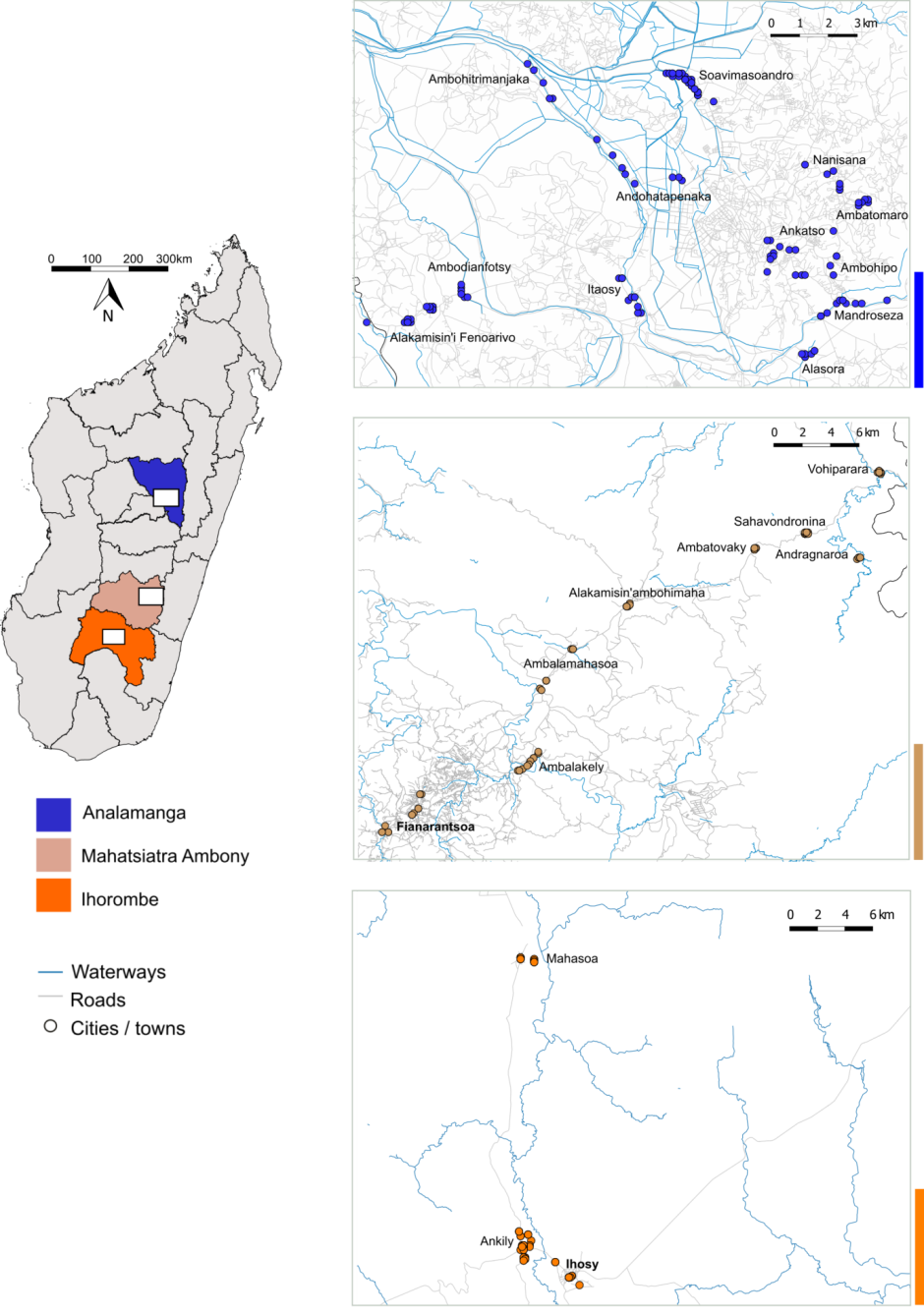


**S2 Figure. Map of survey locations.**


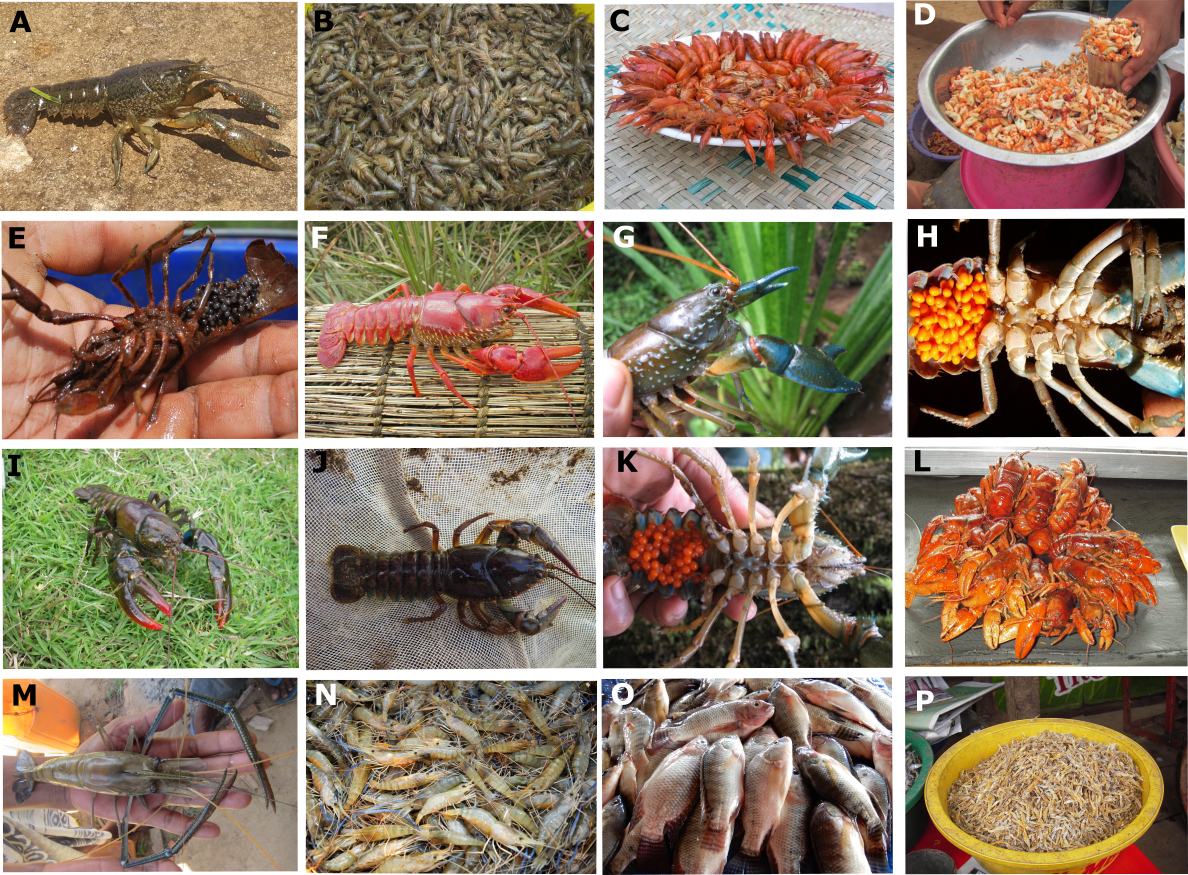


S3 Figure. Pictures of crustaceans and fish for marbled crayfish identification. (A) *Procambarus virginalis*: single animal. (B) *P. virginalis*: batch of live animals. (C) *P. virginalis*: fried animals. (D) *P. virginalis*: tail meat. (E) *P. virginalis*: eggs attached to abdomen. (F) *Astacoides betsileoensis* red: single animal. (G) *A. betsileoensis* blue: single animal. (H) *A. betsileoensis* blue: eggs attached to abdomen. (I) *A. caldwelli*: single animal. (J) *A. madagascariensis*: eggs attached to abdomen. (K) *A. granulimanus*: fried animals. (L) *A. crosnieri:* fried animals. (M) *Macrobrachium* spp.: single animal. (N) *Macrobrachium* spp.: batch of live animals. (O*) Paratilapia* spp.: on market stall. (P) *Gambusia* spp.: dried fish.


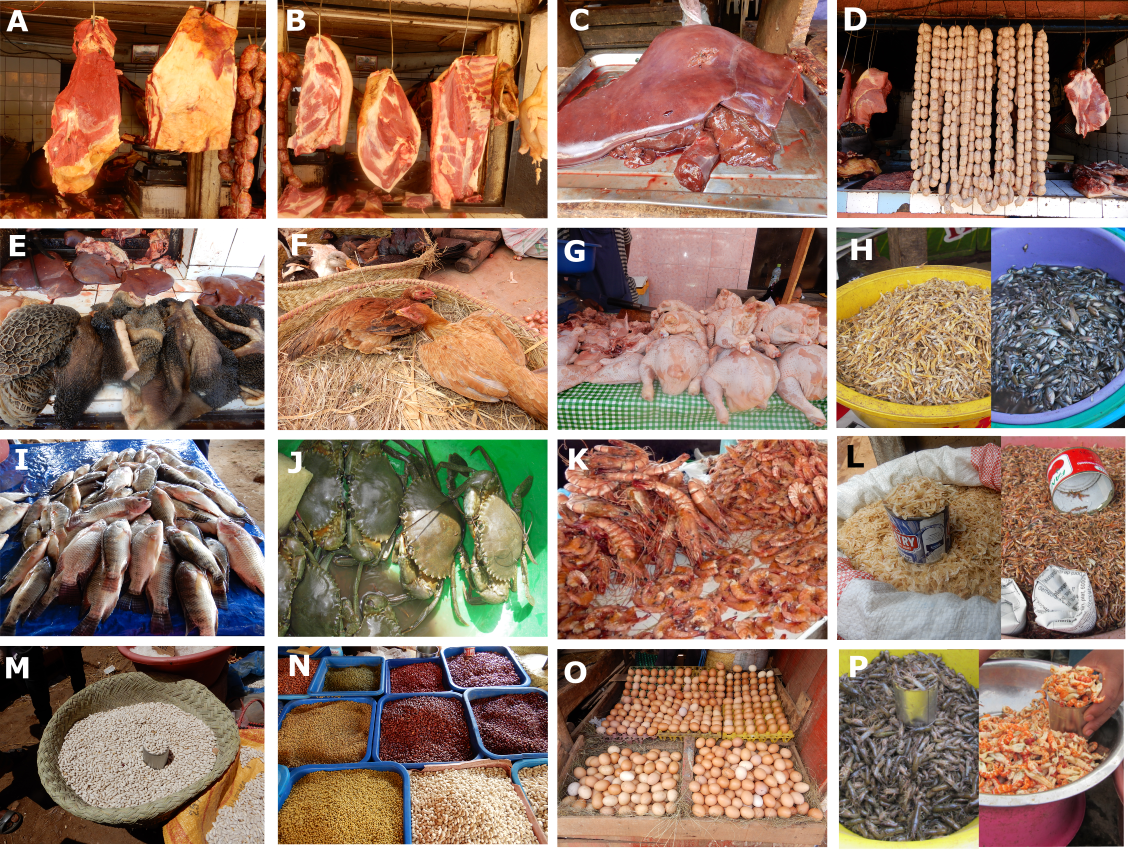


S4 Figure. Pictures representing sources of dietary protein. (A) Zebu meat (*hen’omby*). (B) Pork meat (*hena kisoa*). (C) Zebu liver (*ati-kena*). (D) Sausage (*sosisy*). (E) Tripes (*vorivorin-kena*). (F) Traditionally reared chicken (*akoho gasy*). (G) Factory produced chicken (*poulet de chair*). (H) Tiny fish (*pirina*). (I) Tilapia (*tilapia*). (J) Crabs (*drakaka*). (K) Prawns (*crevettes*). (L) Tiny shrimp (*patsa fotsy/mena*). (M) White beans (*tsaramaso fotsy*). (N) Peas and lentils (*voamaina hafa*). (O) Eggs (*atody*). (P) Marbled crayfish (*foza orana*). Malagasy names are given in brackets.


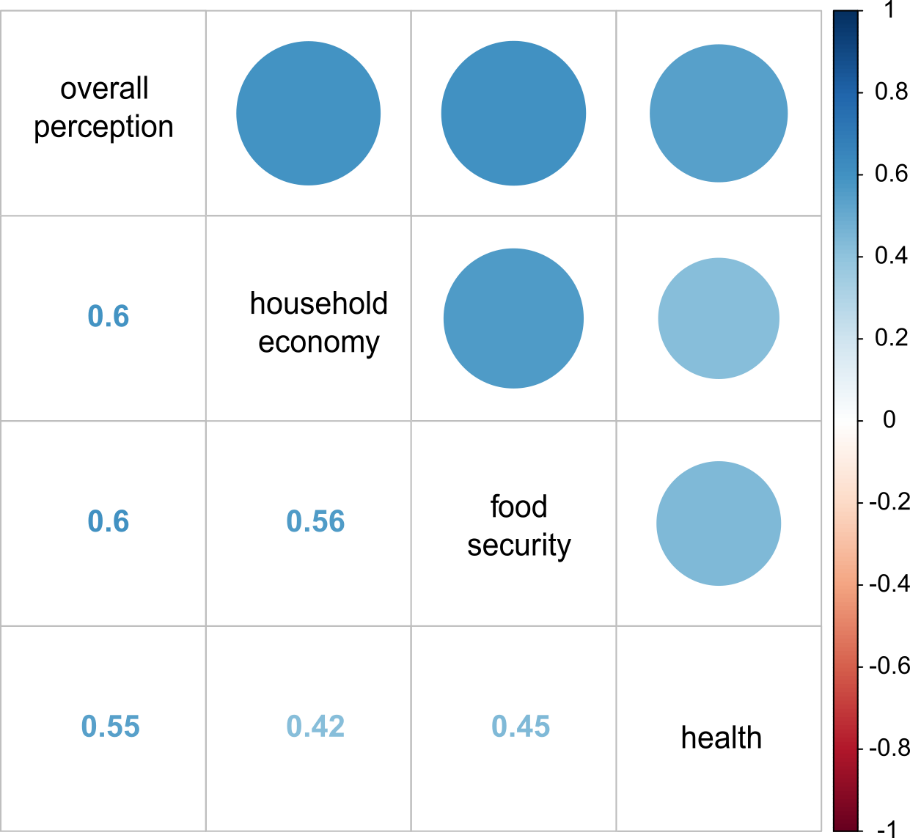


S5 Figure. Bivariate item correlation. Values are Spearman’s Rho. Colour scale indicates a correlation between two items, dark blue being positively correlated and dark red negatively correlated. Circle sizes refer to the strength of the correlation.


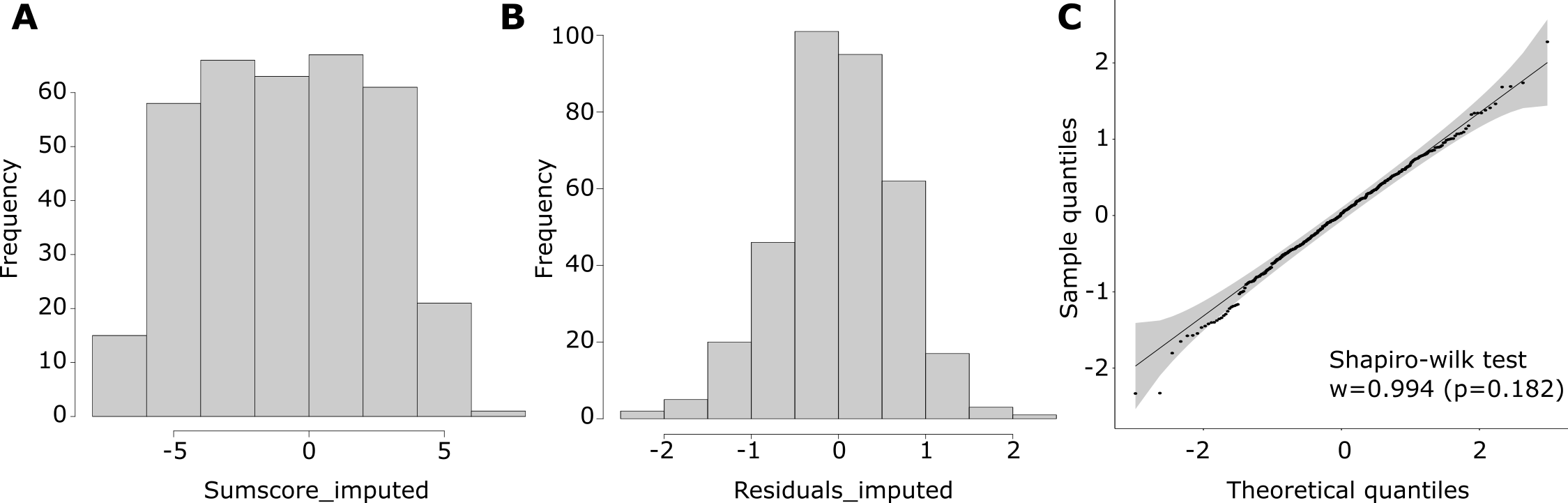


S6 Figure. Histograms and Q-Q plot of data and residuals. (A) Histogram of the imputed sum-scored index. (B) Histogram of the residuals indicating a normal distribution satisfying the assumption of normality. (C) Q-Q plot of the residuals with 95% confidence interval. The linearity of the point satisfies the assumption of normality.

S1 Table. 2019 status of the marbled crayfish presence-absence in three regions previously sampled with no marbled crayfish discovery in 2017 [1].

| **Region** | **District** | **Locality** | **Site** | **Latitude** | **Longitude** | **Status 2017** | **Status 2019** |
| --- | --- | --- | --- | --- | --- | --- | --- |
| **Analanjirofo** | Soanierana Ivongo | Marimbona | River | 16°55.370' | 49°34.657' | NEG | NEG |
|  |  |  | River | 16°55.381' | 49°34.533' | NEG | NEG |
|  |  |  | Fishpond | 16°55.402' | 49°34.602' | NEGD | NEG |
|  |  | Ankoraka | Channel | 16°55.443' | 49°34.810' | NEG | NEG |
|  |  |  | Rice field channel | 16°55.520' | 49°34.794' | NEG | NEG |
|  |  |  | Rice field channel | 16°55.538' | 49°34.767' | NEG | NEG |
|  |  |  | Channel | 16°55.443' | 49°34.329' | NEG | NEG |
|  |  |  | Pond | 16°55.435' | 49°34.605' | **NA** | **NEG** |
|  |  |  | Pond | 16°55.484' | 49°34.606' | **NA** | **NEG** |
|  |  |  | Channel | 16°55.487' | 49°34.799' | **NA** | **NEG** |
|  |  | Ambatarihy | Rice field channel | 16°56.024' | 49°34.501' | **NA** | **NEG** |
|  |  |  | Rice field channel | 16°55.987' | 49°34.546' | **NA** | **NEG** |
|  |  |  | Rice field channel | 16°55.874' | 49°34.583' | **NA** | **NEG** |
| **Menabe** | Miandrivazo | Ampanasana | Marsh | 19°32.477' | 45°27.112' | NEG | NEG |
|  |  |  | Marsh | 19°32.371' | 45°27.364' | NEG | NEG |
|  |  |  | Rice field | 19°32.107' | 45°27.435' | NEG | NEG |
|  |  |  | Rice field | 19°32.094' | 45°27.401' | NEG | NEG |
|  |  |  | Rice field | 19°32.146' | 45°27.327' | NEG | NEG |
|  |  | Mahajilo | Stream | 19°31.295' | 45°27.279' | NEG | NEG |
|  |  | Ankisira | Marsh | 19°34.157' | 45°27.471' | **NA** | **NEG** |
|  |  |  | Rice field channels | 19°34.183' | 45°27.494' | **NA** | **NEG** |
|  |  |  | Marsh | 19°34.332' | 45°27.642' | **NA** | **NEG** |
|  |  |  | Marsh | 19°34.157' | 45°27.471' | **NA** | **NEG** |
|  |  | Ampasambazaha | Rice field | 19°31.638' | 45°27.341' | **NA** | **NEG** |
|  |  |  | Channel | 19°31.701' | 45°27.348' | **NA** | **NEG** |
| **Vatovavy Fitovinany** | Manakara | Andranodaro (Pangalanes) | Stream | 22°09.393' | 48°00.573' | NEG | NEG |
|  |  | Andrena | Channel | 22°09.272' | 48°00.616' | NEG | NEG |
|  |  |  | Rice field channel | 22°09.132' | 48°00.531' | NEG | NEG |
|  |  |  | Channel | 22°08.009' | 48°01.264' | NEG | NEG |
|  |  |  | Stream | 22°08.347' | 48°01.287' | NEG | NEG |
|  |  | Andranodaro | Rice field channels | 22°09.099' | 48°00.606' | **NA** | **NEG** |
|  |  | Ampilao | Rice field channels | 22°08.133' | 48°01.333' | **NA** | **NEG** |

NA: Data not available. NEG: Negative observation (no discovery of marbled crayfish).

**S2 Table. Updated discovery sites of marbled crayfish in discovery regions previously described in 2017** [1].

| **Region** | **District** | **Locality** | **Site** | **Latitude** | **Longitude** | **Status 2017** | **New Stations 2019** |
| --- | --- | --- | --- | --- | --- | --- | --- |
| **Analamanga** | Antananarivo | Anosy | Lake | 18°55,046' | 47°31,388' | POS |  |
|  |  | Atsingita ByPass | Rice field channel | 18°56,821' | 47°31,280' | POS |  |
|  |  | Ankatso Hazo be ravina | Rice field | 18°54,696' | 47°33,448' | POS |  |
|  |  | Ambodianfotsy | Rice field | 18°56,278' | 47°27,680' | POS |  |
|  |  | Miandrarivo Ambanidia | Rice field channel | 18°55,394' | 47°32,278' | POS |  |
|  |  | Ikopa Anosizato Andrefana | Stream | 18°56,269' | 47°29,912' | POS |  |
|  |  | Soavimasoandro | Stream | 18°52.044' | 47°30.927' | **NA** | **POS** |
|  |  |  | Marsh | 18'52.082' | 47°30.688' | **NA** | **POS** |
|  |  |  | Rice field | 18'52.930' | 47°30.600' | **NA** | **POS** |
|  |  | Alakamisin'i Fenoarivo | Rice field | 18°55.866' | 47°26.399' | **NA** | **POS** |
|  |  | Ambohipo | Rice field | 18°55.438' | 47°33.641' | **NA** | **POS** |
|  |  | Andotapenaka | Rice field | 18°53.632' | 47°30.516' | **NA** | **POS** |
|  |  | Mandroseza | Rice field | 18°56.354' | 47°33.352' | **NA** | **POS** |
|  | Ankazobe | Ankazobe | Rice field channel | 18°18,862' | 47°07,272' | POS |  |
| **Mahatsiatra Ambony** | Fianarantsoa | Ambanimaso | Rice field + fishpond | 21°27,915' | 47°04,324' | POS |  |
|  |  | Ambalamena | Rice field | 21°22.214' | 47°10.276' | **NA** | **POS** |
|  | Lalangina | Sahavondronina | Rice field channel | 21°16,579' | 47°20,111' | POS |  |
|  |  | Andragnaroa | Stream | 21°17,551' | 47°22,292' | POS |  |
|  |  |  | Rice field channel | 21°17,567' | 47°22,295' | POS |  |
|  |  | Sahambavy | Lake | 21°26,808' | 47°15,416' | POS |  |
|  |  | Ambatovaky | Rice field | 21°17.252' | 47°18.091' | **NA** | **POS** |
|  |  | Vohiparara | Rice field | 21°14.262' | 47°23.094' | **NA** | **POS** |
|  | Ambohimahasoa | Iarindrano Ambodisahave | Stream | 21°04,051' | 47°05,548' | POS |  |
|  |  |  | Rice field | 21°03,825 | 47°05,583' | POS |  |
| **Ihorombe** | Ihosy | Ankily Ihosy | River | 22°22,512' | 46°06,016' | POS |  |
|  |  | Vatrina | Rice field channel | 22°22,440' | 46°05,722' | POS |  |
|  |  | Ampandratokana | Rice field | 22°22.567' | 46°05.602' | **NA** | **POS** |
|  |  | Ihosy | Pond | 22°22.563' | 46°05.361' | **NA** | **POS** |
|  |  | Ampandra | Pond | 22°23.849' | 46°05.013' | **NA** | **POS** |
|  |  | Mahasoa | Rice field | 22°11.887' | 46°06.168' | **NA** | **POS** |
|  |  |  | River | 22°11.405' | 46°06.372' | **NA** | **POS** |

NA: Data not available. POS: Positive observation (discovery of marbled crayfish).

S3 Table. Geographic coordinates of survey locations.

| **District** | **Localities** | **Latitude** | **Longitude** |
| --- | --- | --- | --- |
| **Antananarivo** | Alakamisin'i Fenoarivo | 18°55.835' | 47°26.185' |
|  | Alasora | 18°57.035' | 47°32.932' |
|  | Ambatomaro | 18°54.122' | 47°34.139' |
|  | Ambodianfotsy | 18°56.172' | 47°27.662' |
|  | Ambohipo | 18°55.732' | 47°33.716' |
|  | Ambohitrimanjaka | 18°52.919' | 47°28.986' |
|  | Andohatapenaka | 18°53.773' | 47°29.693' |
|  | Ankatso | 18°55.031' | 47°32.617' |
|  | Itaosy | 18°56.121' | 47°29.730' |
|  | Mandroseza | 18°56.020' | 47°33.554' |
|  | Nanisana | 18°53.172' | 47°32.869' |
|  | Soavimasoandro | 18°51.811' | 47°30.789' |
| **Fianarantsoa** | Alakamisin'Amabohimaha | 21°19.414' | 47°13.344' |
|  | Ambalakely | 21°24.991' | 47°09.959' |
|  | Ambalamahasoa | 21°21.049' | 47°11.257' |
|  | Ambatovaky | 21°17.257' | 47°18.178' |
|  | Andragnaroa | 21°17.638' | 47°22.085' |
|  | Fianarantsoa city center | 21°26.571' | 47°05.493' |
|  | Sahavondronina | 21°16.655' | 47°20.122' |
|  | Vohiparara | 21°14.337' | 47°22.964' |
| **Ihosy** | Ihosy city center | 22°24.247' | 46°07.678' |
|  | Ankily | 22°23.062' | 46°05.810' |
|  | Mahasoa | 22°11.864' | 46°06.184' |

**S4 Table. Cronbach's Alpha reliability coefficient.**

|  | **raw_alpha** | | **std.alpha** | |
| --- | --- | --- | --- | --- |
|  | 0.8 | | 0.81 | |
|  | **95% confidence interval** | | | |
|  | **lower** | **alpha** | | **upper** |
|  | 0.77 | 0.8 | | 0.84 |
| **Items** | **Reliability if one item is dropped** | | **Item statistics** | |
|  | **raw_alpha** | **std.alpha** | **r_std^1^** | **r_drop^2^** |
| **Overall impact** | 0.72 | 0.72 | 0.88 | 0.72 |
| **Household economy impact** | 0.75 | 0.77 | 0.79 | 0.62 |
| **Food security impact** | 0.75 | 0.76 | 0.79 | 0.64 |
| **Health impact** | 0.79 | 0.8 | 0.73 | 0.55 |

^1^r_std: correlation of each item with the total score (not corrected for item overlap) if the

items were all standardized.

^2^r_drop: item whole correlation for this item against the scale without this item.

**S5 Table. K-cross-validation analysis.**

| **k-fold cross-validation**  **seed set 523** | | |
| --- | --- | --- |
| Linear Regression  352 samples  8 predictors | | |
| No pre-processing  Resampling: Cross-Validated (10-fold)  Summary of sample sizes: 317, 317, 316, 317, 317, 317, … | | |
| **Resampling results** | | |
| **RMSE** | **Rsquared** | **MAE** |
| 0.712 | 0.505 | 0.564 |
| Tuning parameter 'intercept' was held constant at a value of TRUE | | |

S6 Table. Quotes from the semi-structured interviews concerning the impacts of marbled crayfish (with English translations).

|  | **Original quotes in Malagasy** | **English translation** |
| --- | --- | --- |
| **Q1** | Manimba valam-parihy ny foza orana (male farmer, Analamanga) | Marbled crayfish are destroying the rice fields dykes |
| **Q2** | Manimba tahalaka ny foza orana (young male farmer, Mahatsiatra Ambony) |  |
| **Q3** | Manimba tahalaky ny tsipeopeo (older female, Ihorombe) |  |
| **Q4** | Miava ny tany dia maniry tsara ny vary (older female trader, Fianarantsoa) | Marbled crayfish aerate the soil |
| **Q5** | Pirina voany fa ny trondro tsy ataony (older male farmer, Mahatsiatra Ambony). | They can attack tiny fish but not the large ones |
| **Q6** | Mbola tsy tena hita satria vao 2 taona no nampisy azy ka tsy afaka hiteny hoe ratsy be (female farmer, Mahatsiatra Ambony) | The impacts on fishing can’t really be seen since the marbled crayfish are present for only two years |
| **Q7** | Biby kely tsy fantam-piaviana, mahavelona fianakaviana (male Fokontany worker, Analamanga) | A small animal coming from nowhere which supports the needs of the family |
| **Q8** | Mahavelona tokatrano, efa ho 10 taona no nanaovany io asa io fa nahatsara azy (male harvester, Analamanga) | They support the needs of the family, its nearly 10 years I’ve done this work [harvested marbled crayfish]; and its been good |
| **Q9** | Calcium be hoan’ny mpanao baolina mikotrana. Mahazo hery avy amin'ny foza orana (...). Mampisy vitamine sy Calcium, be protein be (female crayfish harvester, Analamanga) | They provide plentiful calcium for football players when they train. They get strength from the marbled crayfish (….). They contain lots of vitamins, calcium and protein |
| **Q10** | Mankarary troky ny tsipeopeo (female farmer, Ihorombe) | Marbled crayfish cause stomach pain |

Description of the survey questionnaire for ODK in English. Modules and questions.

| Module name | Questionnaire: English |
| --- | --- |
| Meta data | Perfect invader survey |
| Meta data | subscriberid |
| Meta data | imei |
| Meta data | simserial |
| Meta data | phonenumber |
| Questionaire_filter | Select the site location |
| Questionaire_filter | Enumerator name |
| Questionaire_filter | Are you conducting the questionnaire or the key informant interview? |
| Questionaire_filter | Is this haphazard or a targeted questionnaire? |
| Ethics | |
| Ethics | Our names are….. We are from….. We are undertaking research into the marbled crayfish. We have asked you to participate because we are simply talking to as many people as we can to get a good understanding of their impacts. |
| Ethics | The exercise will take about 15 minutes, but you are free to leave at any time, you don't have to answer our questions if you don't want to |
| Ethics | The exercise will take about 25 minutes, but you are free to leave at any time, you don't have to answer our questions if you don't want to |
| Ethics | Before we start, we want to make sure that you understand the research we are doing and what we will do with the information. We are not collecting any information which could identify you as an individual. We will use the information in reports we will write (for the Malagasy government and interested people internationally). |
| Ethics | Do you have any questions? |
| Ethics | Consent noted |
| Demography | |
| Demo | Age |
| Demo | Gender |
| Demo | Primary livelihood |
| Demo | Specify 'other' |
| Demo | Secondary livelihood |
| Demo | Specify 'other' |
| Test of knowledge: ability to identify the marbled crayfish | |
| Test of knowledge | Show pictures of the marbled crayfish. Were these correctly identified? |
| Test of knowledge | Show pictures of maximum size scale of marbled crayfish. Were these correctly identified? |
| Food preferences: which protein sources are ranked in the top five? | |
| food preference | Top 5 rank preferences for common meal: 1st choice |
| food preference | 2nd choice |
| food preference | 3rd choice |
| food preference | 4th choice |
| food preference | 5th choice |

| Module name | Questionnaire: English |
| --- | --- |
| Other involvements | |
| other involvement | Which position has the marbled crayfish if it is not in top 5 |
| other involvement | Do you farm rice fields? |
| other involvement | Do you fish for freshwater fish? |
| Involvements in marbled crayfish: economic activities around marbled crayfish | |
| Involvement in MC | Do you harvest marbled crayfish? |
| Involvement in MC | Have you ever farmed marbled crayfish? |
| Involvement in MC | Do you currently farm marbled crayfish? |
| Involvement in MC | For which purpose do you farm marbled crayfish? More information? |
| Involvement in MC | How do you farm marbled crayfish and what are the benefits? |
| Involvement in MC | Do you buy marbled crayfish? |
| Involvement in MC | Do you sell marbled crayfish? |
| Involvement in MC | For how many years do you sell marbled crayfish? |
| Involvement in MC | Is selling marbled crayfish your main activity now? |
| Involvement in MC | What did you do before selling marbled crayfish? |
| Involvement in MC | If selling marbled crayfish is your main activity now, why did you switch from previous activity? |
| Involvement in MC | Is selling marbled crayfish a seasonal work? |
| Involvement in MC | By selling marbled crayfish, do you any comments about business and economic model |
| Involvement in MC | Do you farm animals? |
| Involvement in MC | Do you feed marbled crayfish to animals? |
| Involvements in marbled crayfish as animal feed: which animals are fed with marbled crayfish? | |
| Involvement in MC feed | What do you feed marbled crayfish to? Animal 1 |
| Involvement in MC feed | Animal 2 |
| Involvement in MC feed | Animal 3 |
| Involvement in MC feed | Animal 4 |
| Involvement in MC feed | Animal 5 |
| Involvements in marbled crayfish as animal feed: other animals fed with marbled crayfish? | |
| Involvement in MC animal | Do you feed other animals with marbled crayfish? |
| Involvement in MC animal | What other animal do you marbled crayfish to? |
| Involvement in MC animal | Any further comment on feeding animals with marbled crayfish? |

| Module name | Questionnaire: English |
| --- | --- |
| Marbled crayfish impacts: marbled crayfish history | |
| MC impact | Do you know when marbled crayfish first appeared in this area? |
| MC impact | When was the earliest marbled crayfish might have appeared? |
| MC impact | When was the latest marbled crayfish might have appeared? |
| MC impact | Do you know how marbled crayfish got there? |
| MC impact | How did marbled crayfish got there? |
| MC impact | Have marbled crayfish affected people's life in your surroundings compared to when they were not here? |
| MC impact | How did marbled crayfish affected people's life in your surroundings? |
| MC impact | Have marbled crayfish affected farming such as rice farming or fish farming or other farming activities? |
| MC impact | How the marbled crayfish are used? |
| Involvements in Astacoides: knowledge on Astacoides (native crayfish species) | |
| Involvement in Astacoides | Do you know Astacoides? (Were they correctly identified?) |
| Involvement in Astacoides | Do you harvest Astacoides? |
| Involvement in Astacoides | Do you sell Astacoides? |
| Involvement in Astacoides | Do you buy Astacoides |
| Involvement in Astacoides | What is the trend in abundance of Astacoides (relative to 2009) |
| Involvement in Astacoides | What is the main reason for this change of abundance in Astacoides? |
| Involvement in Astacoides | Do you find marbled crayfish in the same streams as Astacoides? |
| Marbled crayfish specific impacts. | |
| MC specific impact | How do you perceive the impacts of marbled crayfish on rice farming? |
| MC specific impact | Open ended follow-up – further information about impact on rice farming? |
| MC specific impact | How do you perceive the impacts of marbled crayfish on fishing? |
| MC specific impact | Open ended follow-up – which fish and how does marbled crayfish impact on fishing? |
| MC specific impact | How do you perceive the impacts of marbled crayfish on your household income? (relative to if marbled crayfish didn't exist in the area) |
| MC specific impact | Follow up? |
| MC specific impact | How do you perceive the impacts of marbled crayfish on food security through household consumption? (relative to if marbled crayfish didn't exist in the area) |
| MC specific impact | Follow up? |
| MC specific impact | How do you perceive the impacts of marbled crayfish on your health? (relative to if marbled crayfish didn't exist in the area) |
| MC specific impact | Follow up? |
| MC specific impact | Perceived impacts on household economy through use of animal feed? |
| MC specific impact | Follow up? |

| Module name | Questionnaire: English |
| --- | --- |
| MC specific impact | Have you noticed an impact of the arrival of marbled crayfish on Astacoides populations? |
| MC specific impact | Do you have any comments about interactions between Astacoides and Marbled crayfish? |
| MC specific impact | Other perceived impacts of marbled crayfish in general? |
| MC specific impact | How do you perceive the impact of marbled crayfish overall? |
| MC specific impact | Any other information on the impacts of marbled crayfish? |
| Ethics | |
| ethics2 | [If appropriate] If you agree, we would like to take some photos. We might use these in presentations or publications about this project. |
| Location details | |
| final | Fokontany |
| final | Commune |
| final | GPS location of interview location |

ODK: Open Data Kit. MC: marbled crayfish. GPS: Global Positioning System.

Description of the survey questionnaire for ODK in Malagasy. Modules and questions.

| Module name | Questionnaire:Malagasy |
| --- | --- |
| Meta data | Perfect invader survey |
| Meta data | subscriberid |
| Meta data | imei |
| Meta data | simserial |
| Meta data | phonenumber |
| Questionnaire filter | |
| Questionaire_filter | Hisafidy toerana |
| Questionaire_filter | Anaran'ny mpanadihady |
| Questionaire_filter | Fanadihadiahana tsotra sa voatondro manokana no ataonao? |
| Questionaire_filter | Nosafidiana manokana ve kisendra ny olona hadihadianao? |
| Ethics | |
| Ethics | …… no anaranay. Avy ao amin'ny sampana …... no misy anay. Manao fikarohana momban'ny foza orana izahay mba ahafahana mandalina sy mahazo be be kokoa ny vokatra mety hoaterak'izy ireo. Noho izany dia manadihady olona maro be izahay ka izany no antony angatahanay ny fandraisanao anjara. |
| Ethics | Maharitra eo amin'ny 15 minitra eo ny fanadihadihana fa manan-jo handeha ianao na ovina na oviana ary tsy an-tery ny famaliana ny fanontaniana. |
| Ethics | Maharitra eo amin'ny 25 minitra eo ny fanadihadihana fa manan-jo handeha ianao na ovina na oviana ary tsy an-tery ny famaliana ny fanontaniana. |
| Ethics | Alohan'ny hanombohantsika dia tianay ho fantatra hoe azonao tsara ve ny antony hanaovanay izao fikarohana izao sy ny fomba hampiasaina ny vokatra. Tsy manadihadihady ny mombanao manokana izahay, izany hoe tsy ho fantatra mihitsy hoe hevitrao no voalaza mandritry ny fanadihadiana. Ny vokatry ny fanadihadiana dia hoampiasaina hanoratana sy hanaovana tatitra eo anivon'ny fitondram-panjakana sy hoan'ireo olona izay liana eo amin'ny sehatra iraisam-pirenena. |
| Ethics | Sao dia misy fanontaniana hafa manitikitika anao? |
| Ethics | Fanomezana alalana voaray |
| Demography | |
| Demo | Taona |
| Demo | Karazana |
| Demo | Foto-pivelomana |
| Demo | Mariho raha toa ka "hafa" |
| Demo | Fivelomana fanampiny |
| Demo | Mariho raha toa ka "hafa" |

| Module name | Questionnaire:Malagasy |
| --- | --- |
| Test of knowledge: ability to identify the marbled crayfish | |
| Test of knowledge | Asehoy ny sarin'ny foza orana. Marina tsara ve ny fahafantarany azy? |
| Test of knowledge | Asehosy ny sarin'ny hahabeny. Marina tsara ve ny fahafantarany azy? |
| Food preferences: which protein sources are ranked in the top five? | |
| food preference | reo laoka mahazatra 5 tena ankafizina: safidy voalohany |
| food preference | Safidy faha-2 |
| food preference | Safidy faha-3 |
| food preference | Safidy faha-4 |
| food preference | Safidy faha-5 |
| Other involvements | |
| other involvement | Aiza ny toerana misy ny foza orana raha toa ka tsy ao anaty safidy 5 voalohany? |
| other involvement | Mamboly vary ve ianao (tanimbary)? |
| other involvement | Manjono / Mamintana trondon-dranomamy ve ianao? |
| Involvements in marbled crayfish: economic activities around marbled crayfish | |
| Involvement in MC | Misaka foza orana ve ianao? |
| Involvement in MC | Efa niompy foza orana ve ianao hatramin'izay? |
| Involvement in MC | Miompy foza orana ve ianao amin'izao fotoana izao? |
| Involvement in MC | Inona no antony hiompianao foza orana? Fanamarihana fanampiny? |
| Involvement in MC | Ahoana ny fomba fiompianao foza orana ary inona no tombontsoa azonao amin'izany? |
| Involvement in MC | Mividy foza orana ve ianao? |
| Involvement in MC | Mivarotra foza orana ve ianao? |
| Involvement in MC | Hafiriana izay no nivarotanao foza orana (taona)? |
| Involvement in MC | Mivarotra foza orana ve no foto-pivelomanao? |
| Involvement in MC | Inona ny asanao taloha raha mivarotra foza orana no foto-pivelomanao taloha? |
| Involvement in MC | Inona ny antony nialanao tamin'ny asanao taloha raha toa ka mivarotra foza orana no foto-pivelomanao taloha? |
| Involvement in MC | Mandavataona ve sa mandritry ny vanim-potoana manokana no hivarotanao foza orana? |
| Involvement in MC | Amin'ny fivarotana foza orana, manana soso-kevitra eo amin'ny fandehan'ny tsena sy ny varotra ve ianao? |
| Involvement in MC | Manana biby fiompy ve ianao? |
| Involvement in MC | Ampiasainao ho sakafon'ireo biby fiompinao ireo ve ny foza orana? |
| Involvements in marbled crayfish as animal feed: which animals are fed with marbled crayfish? | |
| Involvement in MC feed | Inona avy ireo biby omenao foza orana ho sakafo? Biby 1 |
| Involvement in MC feed | Biby 2 |
| Involvement in MC feed | Biby 3 |
| Involvement in MC feed | Biby 4 |
| Involvement in MC feed | Biby 5 |

| Module name | Questionnaire:Malagasy |
| --- | --- |
| Involvements in marbled crayfish as animal feed: other animals fed with marbled crayfish? | |
| Involvement in MC animal | Misy biby hafa ankoatr'ireo omenao foza orana ho sakafo ve? |
| Involvement in MC animal | Inona avy biby hafa omenao foza orana ho sakafo? |
| Involvement in MC animal | Fanamarihana fanampiny momban'ny fanomezana foza orana ho sakafom-biby? |
| Marbled crayfish impacts: marbled crayfish history | |
| MC impact | Fantatrao ve hoe oviana no nisian'ny foza orana voaloha teto amin'ity faritra ity? |
| MC impact | Oviana raha aloha indrindra no nisian'ny foza orana voalohany teto? |
| MC impact | Oviana raha taraiky indrindra no nisian'ny foza orana voalohany teto? |
| MC impact | Fantatrao ve ny fomba nahatongavan'ny foza orana teto? |
| MC impact | Ahoana ny fomba nahatongavan'ny foza orana teto? |
| MC impact | Nisy fiantraikany eo amin'ny fiainan'olona manodidina anao ve ny fisian'ny foza orana raha oharina amin'ny tsy fisiany taloha? |
| MC impact | Inona avy ireo fiantraika nateraky ny foza orana teo amin'ny olona manodidina anao? |
| MC impact | Misy fiantraikany eo amin'ny fiompiana ve ny fisian'ny foza orana raha oharina amin'ny tsy fisiany taloha? |
| MC impact | Inona avy ny fomba ampiasana ny foza orana? |
| Involvements in Astacoides: knowledge on Astacoides (native crayfish species). | |
| Involvement in Astacoides | Mihaza orana zana-tany ve ianao (oramena, orambato)? |
| Involvement in Astacoides | Mivarotra orana ve ianao? |
| Involvement in Astacoides | Mividy orana ve ianao? |
| Involvement in Astacoides | Inona ny zava-mitranga eo amin'ny habetsahan'ny orana zana-tany? |
| Involvement in Astacoides | Inona ny anton'izay fiovana izay? |
| Involvement in Astacoides | Efa nahitanao foza orana ve ny renirano misy ny orana zana-tany? |
| Marbled crayfish specific impact | |
| MC specific impact | Vokatra tsapa eo amin'ny fambolem-bary? |
| MC specific impact | Hevitra, fanazavana na fapahafantarana fanampiny? |
| MC specific impact | Vokatra tsapa eo amin'ny jono? |
| MC specific impact | Hevitra, fanazavana na fapahafantarana fanampiny eo amin'ireo karazana trondro? Amin'ny fomby ahoana? |
| MC specific impact | Vokatra tsapa eo amin'ny vola miditra ao an-trano? (raha oharina amin'ny hoe: tsy nisy foza orana eo amin'ny faritra). |
| MC specific impact | Hevitra fanampiny? |
| MC specific impact | Vokatra tsapa eo amin'ny fahampian'ny sakafo ao an-trano amin'ny fihinana foza orana? (raha oharina amin'ny hoe tsy nisy foza orana eo amin'ny faritra). |
| MC specific impact | Hevitra fanampiny? |
| MC specific impact | Vokatra tsapa amin'ny lafin'ny fahasalamana? (raha oharina amin'ny hoe: tsy nisy foza orana eo amin'ny faritra). |

| Module name | Questionnaire:Malagasy |
| --- | --- |
| MC specific impact | Hevitra fanampiny? |
| MC specific impact | Vokatra tsapa eo amin'ny tahiry ao an-tokantrano amin'ny fampiasana foza orana ho sakafom-biby? |
| MC specific impact | Hevitra fanampiny? |
| MC specific impact | Hatramin’izay nahatongavan'ny foza orana, nahatsikaritra fiovana teo amin'ny orana zana-tany ve ianao? |
| MC specific impact | Manan-kevitra na misy holazaina momban'ny fifandraisany misy eo amin'ny foza orana sy ny orana zana-tany ve ianao? |
| MC specific impact | Vokatra hafa tsapa? |
| MC specific impact | Voakatra tsapa amin'ny ankapobeany |
| MC specific impact | Misy zavatra mety fantatrao nefa tsy nanontaninay momban'ny foza orana ve? (Raiso daholo azafady izay zavatra rehetra mety manan-danja na mahaliana antsika, indrindra momban'ny vokatra ateraky ny foza orana, izay voalazan'ilay olona voahadihady). |
| Ethics | |
| ethics2 | [Raha toa ka mety] / [Raha to ka ilaina] Raha toa ianao ka manome alalana dia mba haka sary vitsivitsy izahay. Mety hoampiasaina ny sary amin'ny tatitra izay ho atao na amin'ny gazety siantifika momban'ity tetik'asa ity izy. |
| Location details | |
| final | Fokontany |
| final | Kaominina |
| final | GPS toerana hanaovana ny fahadihadihana |

ODK: Open Data Kit. MC: marbled crayfish. GPS: Global Positioning System.

**Reference**

1. Gutekunst J, Andriantsoa R, Falckenhayn C, Hanna K, Stein W, Rasamy J, et al. Clonal genome evolution and rapid invasive spread of the marbled crayfish. Nat Ecol Evol. 2018;2: 567–573. doi:10.1038/s41559-018-0467-9.
