# Supplementary material for: Perceived socio-economic impacts of the marbled crayfish invasion in Madagascar
Source: PLoS One. 2020 Apr 15;15(4):e0231773. doi: 10.1371/journal.pone.0231773 (PMC7159205; doi:10.1371/journal.pone.0231773)
Supplement: Attachment — Submitted filename: PONE-D-19-30933.pdf [file pone.0231773.s002.pdf]

PONE-D-19-30933

Review of: *Socio-economic impacts of the marbled crayfish invasion in Madagascar*

Overview

This manuscript is about perceptions of marbled crayfish in Madagascar; some people find them to be acceptable, whereas others do not. Predictably, the responses are based on livelihoods. I think it provides a nice sense of balance to invasion ecology, instead of focusing exclusively on the negative aspects. The naturalistic setting makes the study particularly interesting. Although I have not been to Madagascar, I could envision this situation quite easily having been to other developing countries.

A convenience sample is not preferable, but several interventions were employed to overcome this issue: a large sample size, a variety of sampling locations, and an excellent response rate. The study was well-supported by the literature, followed by some statistical tests, and nice conclusions.

Limitations

- The manuscript is plagued with improper English and needs considerable attention to detail before it is acceptable for publication (i.e., redundancies, contradictions, verb tense, splitting verbs, misspellings, and run-on sentences). I made many edits throughout the paper (see PDF) for consideration in the next round. For example, why is it important to mention that a Likert scale was used 5 times? What is a substantial fraction (did you mean percent)? Why did you say, tilapia fish? The word ‘order’ is used twice in the same sentence; once as a noun and once as a verb.
- Terminology, in some cases, was inconsistent with traditional usage in the social sciences. For example, it was a convenience sample, not haphazard or opportunistic.
- Although the sampling period was identified (March and April, 2019), it did not include information on weekday vs. weekend data collection.
- The perception of marbled crayfish was measured by 6 items (not aspects), yet a Cronbach’s alpha (reliability coefficient) was not provided.
- The dependent variable (perception of impacts) was measured on a 5-point scale and referred to as ordinal data. Most social science research uses a 5-point scale and calls it interval data. Your choice of statistics hinges upon this decision. Please re-run your data using linear regression to see how the results compare with OR and OLS. Perhaps you can improve R^2 even more than it is already.
- I did not see a sample size of key informants, or a description on how you analyzed this data. I saw some direct quotes in the narrative, but it would be better if you had categorized the open-ended information so I could see it in a table.
