# Supplementary material for: Perceived socio-economic impacts of the marbled crayfish invasion in Madagascar
Source: PLoS One. 2020 Apr 15;15(4):e0231773. doi: 10.1371/journal.pone.0231773 (PMC7159205; doi:10.1371/journal.pone.0231773)
Supplement: Attachment — Submitted filename: PONE-D-19-30933_peer review.pdf [file pone.0231773.s003.pdf]

PLOS ONE

Socio-economic impacts of the marbled crayfish invasion in Madagascar

--Manuscript Draft--

**Manuscript Number:**	PONE-D-19-30933
**Article Type:**	Research Article
**Full Title:**	Socio-economic impacts of the marbled crayfish invasion in Madagascar
**Short Title:**	Marbled crayfish impact in Madagascar
**Corresponding Author:**	Frank Lyko<br>Deutsches Krebsforschungszentrum<br>Heidelberg, Heidelberg GERMANY
**Keywords:**	
**Abstract:**	The negative environmental and economic impacts of many invasive species are well known. However, given the increased homogenization of the global biota, and the difficulty of eradicating species once established, a balanced approach to considering the impacts of invasive species is needed. The marbled crayfish (Procambarus virginalis) is parthenogenetic freshwater crayfish that was first observed in Madagascar around 2005 and has spread rapidly. We present the results of a socio-economic survey (n=385) in three regions of Madagascar that vary in when marbled crayfish first arrived. Respondents generally considered marbled crayfish to have a negative impact on rice agriculture and fishing, however the animals were also seen as making a positive contribution to household economy and food security. Regression modeling shows that those in regions with longer experience with marbled crayfish have more positive perceptions. Unsurprisingly, considering the perception that crayfish negatively impact rice agriculture, farmers have more negative views towards the crayfish than those involved in crayfish trading. Food preference ranking and market surveys reveal the acceptance of marbled crayfish as a source of cheap animal protein; a clear positive in a country with widespread malnutrition. While data on the biodiversity impacts of the marbled crayfish invasion in Madagascar is still completely lacking, this study provides insight into the socio-economic impacts of the dramatic spread of this unique invasive species in Madagascar.
**Order of Authors:**	Ranja Andriantsoa
	Julia Jones
	Vlad Achimescu
	Heriniaina Randrianarison
	Miary Raselimanana
	Manjary Andriantsitohaina
	Jeanne Rasamy
	Frank Lyko
**Additional Information:**	
**Question**	**Response**
**Financial Disclosure**<br><br>Enter a financial disclosure statement that describes the sources of funding for the work included in this submission. Review the submission guidelines for detailed requirements. View published research articles from *PLOS ONE* for specific examples.	The author(s) received no specific funding for this work.

Powered by Editorial Manager® and ProduXion Manager® from Aries Systems Corporation

This statement is required for submission and **will appear in the published article** if the submission is accepted. Please make sure it is accurate.<br><br>**Unfunded studies**<br>Enter: *The author(s) received no specific funding for this work.*<br><br>**Funded studies**<br>Enter a statement with the following details:<br>• Initials of the authors who received each award<br>• Grant numbers awarded to each author<br>• The full name of each funder<br>• URL of each funder website<br>• Did the sponsors or funders play any role in the study design, data collection and analysis, decision to publish, or preparation of the manuscript?<br>• **NO** - Include this sentence at the end of your statement: *The funders had no role in study design, data collection and analysis, decision to publish, or preparation of the manuscript.*<br>• **YES** - Specify the role(s) played.<br><br>* typeset	
**Competing Interests**<br><br>Use the instructions below to enter a competing interest statement for this submission. On behalf of all authors, disclose any competing interests that could be perceived to bias this work—acknowledging all financial support and any other relevant financial or non-financial competing interests.<br><br>This statement **will appear in the published article** if the submission is accepted. Please make sure it is accurate. View published research articles from *PLOS ONE* for specific examples.	The authors have declared that no competing interests exist.

Powered by Editorial Manager® and ProduXion Manager® from Aries Systems Corporation

**NO authors have competing interests**<br><br>Enter: *The authors have declared that no competing interests exist.*<br><br>**Authors with competing interests**<br><br>Enter competing interest details beginning with this statement:<br><br>*I have read the journal's policy and the authors of this manuscript have the following competing interests: [insert competing interests here]*<br><br>* typeset	
**Ethics Statement**<br><br>Enter an ethics statement for this submission. This statement is required if the study involved:<br><br>• Human participants<br>• Human specimens or tissue<br>• Vertebrate animals or cephalopods<br>• Vertebrate embryos or tissues<br>• Field research<br><br>Write "N/A" if the submission does not require an ethics statement.<br><br>General guidance is provided below. Consult the submission guidelines for detailed instructions. **Make sure that all information entered here is included in the Methods section of the manuscript.**	Research involving human participants was approved by the Bangor University College of Environmental Sciences and Engineering research ethics committee (Approval Number: CoESE2019RAJPGJ01). Oral consent was obtained from each participant to voluntarily participate in the survey. Field research was approved by the Ministry of Environment and Forest of Antananarivo, Madagascar (research permits No. 58/19/MEDD/SG/DGF/DSAP/SCB.Re and No. 59/19/MEDD/SG/DGF/DSAP/SCB.Re).

Powered by Editorial Manager® and ProduXion Manager® from Aries Systems Corporation

**Format for specific study types**<br><br>**Human Subject Research (involving human participants and/or tissue)**<br>• Give the name of the institutional review board or ethics committee that approved the study<br>• Include the approval number and/or a statement indicating approval of this research<br>• Indicate the form of consent obtained (written/oral) or the reason that consent was not obtained (e.g. the data were analyzed anonymously)<br><br>**Animal Research (involving vertebrate animals, embryos or tissues)**<br>• Provide the name of the Institutional Animal Care and Use Committee (IACUC) or other relevant ethics board that reviewed the study protocol, and indicate whether they approved this research or granted a formal waiver of ethical approval<br>• Include an approval number if one was obtained<br>• If the study involved *non-human primates*, add *additional details* about animal welfare and steps taken to ameliorate suffering<br>• If anesthesia, euthanasia, or any kind of animal sacrifice is part of the study, include briefly which substances and/or methods were applied<br><br>**Field Research**<br><br>Include the following details if this study involves the collection of plant, animal, or other materials from a natural setting:<br>• Field permit number<br>• Name of the institution or relevant body that granted permission	
**Data Availability**<br><br>Authors are required to make all data underlying the findings described fully available, without restriction, and from the time of publication. PLOS allows rare exceptions to address legal and ethical concerns. See the PLOS Data Policy and FAQ for detailed information.	Yes - all data are fully available without restriction

Powered by Editorial Manager® and ProduXion Manager® from Aries Systems Corporation

A Data Availability Statement describing where the data can be found is required at submission. Your answers to this question constitute the Data Availability Statement and **will be published in the article**, if accepted.<br><br>**Important:** Stating 'data available on request from the author' is not sufficient. If your data are only available upon request, select 'No' for the first question and explain your exceptional situation in the text box.<br><br>Do the authors confirm that all data underlying the findings described in their manuscript are fully available without restriction?	
**Describe where the data may be found in full sentences. If you are copying our sample text, replace any instances of XXX with the appropriate details.**<br><br>• If the data are **held or will be held in a public repository**, include URLs, accession numbers or DOIs. If this information will only be available after acceptance, indicate this by ticking the box below. For example: *All XXX files are available from the XXX database (accession number(s) XXX, XXX.*).<br>• If the data are all contained **within the manuscript and/or Supporting Information files**, enter the following: *All relevant data are within the manuscript and its Supporting Information files.*<br>• If neither of these applies but you are able to provide **details of access elsewhere**, with or without limitations, please do so. For example:<br><br>*Data cannot be shared publicly because of [XXX]. Data are available from the XXX Institutional Data Access / Ethics Committee (contact via XXX) for researchers who meet the criteria for access to confidential data.*<br><br>*The data underlying the results presented in the study are available from (include the name of the third party*	All relevant data are within the manuscript and its Supporting Information files.

and contact information or URL).<br>• This text is appropriate if the data are owned by a third party and authors do not have permission to share the data.<br><br>* typeset	
Additional data availability information:	

Manuscript

Click here to access/download;Manuscript;ms_submitted_ed2.docx

Socio-economic impacts of the marbled crayfish invasion in Madagascar

Ranja Andriantsoa[1], Julia P.G. Jones[2], Vlad Achimescu[3], Heriniaina Randrianarison[4], Miary Raselimanana[4], Manjary Andriantsitohaina[4], Jeanne Rasamy[4], Frank Lyko[1]

[1]Division of Epigenetics, DKFZ-ZMBH Alliance, German Cancer Research Center (DKFZ), 69120 Heidelberg, Germany; [2]School of Environmental Sciences and Engineering, Bangor University, Bangor LL57 2UW, UK, [3]School of Social Science, Mannheim University, 68159 Mannheim, Germany; [4]Mention Zoologie et Biodiversité Animale, Université d'Antananarivo, BP906, 101 Antananarivo, Madagascar;

Corresponding author: Frank Lyko
Deutsches Krebsforschungszentrum
Im Neuenheimer Feld 580
69120 Heidelberg, Germany
phone: +49-6221-423800
fax: +49-6221-423802
email: f.lyko@dkfz.de

Short title: Marbled crayfish impact in Madagascar

Abstract

Negative impacts of invasive species often overshadow the positive values associated with their presence. However, management should consider the advantages and disadvantages of invasive species, including potential human benefits, before implementing any control strategies. ~~The negative environmental and economic impacts of many invasive species are well known. However, given the increased homogenization of the global biota, and the difficulty of eradicating species once established, a balanced approach to considering the impacts of invasive species is needed.~~ The marbled crayfish (*Procambarus virginalis*) is a parthenogenetic freshwater crayfish that was first observed in Madagascar around 2005 and is ~~has~~ spreading rapidly. We present the results of a social ~~o-economic~~ survey (n=385) in three regions of Madagascar that show various levels of infestation. ~~vary in when marbled crayfish first arrived.~~ Many r~~R~~espondents ~~generally~~ considered the marbled crayfish were seen as making a positive contribution ~~to~~ to the household economy and food security, especially those involved in crayfish trading. Unsurprisingly, farmers expressed more negative views toward the crayfish since they have can a harmful effect ~~negative impact~~ on rice agriculture and fishing~~, however the animals were also seen as making a positive contribution to household economy and food security.~~ . Regression modeling showed~~s~~ that individuals living ~~those~~ in regions having more ~~with longer~~ experience with marbled crayfish had~~ve~~ more positive perceptions of them. ~~Unsurprisingly, considering the perception that crayfish negatively impact rice agriculture, farmers have more negative views towards the crayfish than those involved in crayfish trading.~~ Food preference ranking and market surveys revealed the acceptance of marbled crayfish as a source of cheap animal protein; a clear benefit ~~positive~~ in a country with widespread malnutrition. While data on the biodiversity impacts of the marbled crayfish invasion in Madagascar is still ~~completely~~ lacking, this study provides insight into the socio-economic impacts of the dramatic spread of this ~~unique~~ invasive species across the country~~in Madagascar~~.

Commented [MM1]: As you can tell, I revised your first two sentences considerably. I thought your version was a bit too wordy for the abstract. If you like yours better than mine, then change it back. Or provide another example

"*Biby kely tsy fantam-piaviana, mahavelona fianakaviana*" (a small animal coming from who knows where which supports the needs of the family). Fokontany worker Analamanga, Madagascar.

Introduction

Invasive alien~~s~~ species can have substantial negative effects on the environment, the e~~E~~conomy, and ~~the~~ human health [1–3]. For example, changes in ~~Invasive species are major drivers of change in invaded ecosystems causing~~ habitat structure and function, and ~~modification, affecting~~ biodiversity, can ~~composition, and even sometimes~~ result ~~ing~~ in extinction of species through direct predation or competition for food and space with other species [4–6]. The damage caused by invasions and the management costs for ~~the~~ control can be a significant economic burden [7]. Also, invasive species can play important roles in disease transmission and spread [3]. ~~While r~~Research tends to ~~has traditionally~~ focus~~ed~~ on problems associated with invasive species ~~these negative impacts, potential benefits from biological invasions can be overlooked. This may be~~ because negative impacts are part of the definition ~~of invasive species~~ [8]. Yet, ~~but~~ there is an increasing awareness of their ~~the potential~~ positive contributions ~~made by invasive species~~ [9–11]. Few studies have explored how perceptions of costs and benefits from an invasion may vary among people living with an invasive species and how this may change after ~~over time following~~ an invasion occurs.

Invasive crayfish have been recognized as a major threat to freshwater ecosystems. Indeed, their position in the food web (most are omnivorous) and also their capacity to transmit diseases can ~~strongly~~ impact ~~an~~ ecosystem functions. The rusty crayfish (*Faxonius rusticus*), for example, can ~~significantly~~ reduce resource availability in aquatic communities leading to biodiversity loss, while the signal crayfish (*Pacifastacus leniusculus*) transmits the crayfish plague in Europe, ~~which has~~ resulting ~~ed~~ in extirpation of native crayfish from many locations [12,13]. Nonetheless, invasive crayfish species can also play an important ~~positive~~ socio-economic role. *Procambarus clarkii* supports important commercial fisheries in many countries where it has

been introduced [14,15], while the red claw crayfish (*Cherax quadricarinatus*) is an important

income source for artisanal fishermen in Jamaica [16]. Studies have also suggested that invasive crayfish in Kenya play a role in control of schistosomiasis through predation on the snail hosts of this human parasite [17].

The marbled crayfish (*Procambarus virginalis*) originated from the German aquarium trade in the early 2000s [18,19]. Its ability to reproduce without males, as well as its high fecundity and resistance to harsh environmental conditions, predisposes it to be an invasive species [20,21]. Indeed, marbled crayfish have formed stable wild colonies in several European countries [22–26]. In Madagascar, marbled crayfish were first observed by biologists in markets around 2005, but were not identified until 2007 [21,27]. Initially confined to a small region around Antananarivo, their distribution area has now increased about 100-fold, covering eight out of 15 regions studied [28]. Conservationists quickly raised concerns [19,21] about potential impacts that marbled crayfish may have on ~~endemic species, including~~ 7 species of endemic crayfish in the genus *Astacoides* [29,30], should the invasion ~~ve species~~ overlap into the range of native species~~ range~~. In addition, there were also concerns that marbled crayfish might ~~may~~ have a negative impact on rice agriculture and local ~~harvested~~ fisheries. ~~populations.~~ However, ~~from the start,~~ marbled crayfish were found in markets and farmers were observed transporting the species intentionally around the island as early as 2007, suggesting that they were considered valuable [21]. More recently they have been observed as being widely sold in markets across Madagascar and being harvested in rice fields [31].

Our study aims to update information on the distribution of marbled crayfish in Madagascar and to explore attitudes toward~~s~~ marbled crayfish in three regions varying in how recently they ~~marbled crayfish~~ arrived. Specifically, we explore perceptions of ~~the positive or negative~~ impacts on rice agriculture, fishing and its contribution to health, food security, and household income. We also studied ~~sought to understand more about~~ the markets for this species, including the relative acceptance of marbled crayfish as a source of dietary protein. While information on the ecological impacts of ~~the invasion of~~ marbled crayfish in Madagascar are ~~is still entirely~~ lacking, this paper provides some valuable information on their socio-economic impacts.

Materials and methods

Ethics statement

Research involving human participants was approved by the Bangor University College of Environmental Sciences and Engineering research ethics committee (Approval Number: CoESE2019RAJPGJ01). Oral consent was obtained from each participant to voluntarily participate in the survey. Field research was approved by the Ministry of Environment and Forest of Antananarivo, Madagascar (research permits No. 58/19/MEDD/SG/DGF/DSAP/SCB.Re and No. 59/19/MEDD/SG/DGF/DSAP/SCB.Re).

Update of marbled crayfish distribution

Field~~-~~work exploring the distribution of marbled crayfish distribution was conducted in March and April 2019 in three regions where marbled crayfish were not discovered in 2017 (Analanjirofo, Menabe, Atsimo Andrefana). For each region, we performed re-collection in each of our previous sampling stations [28] with additional new stations in the same area. We sampled the water bodies with traditional nets “tandroho” (50cm x 30 cm x 30cm) morning from 8:00 to 11:00 and from 15:00 to 18:00 for three days. Collections were performed by M.A.

Social survey details

The survey was conducted ~~carried out~~ in March and April 2019 in three regions where marbled crayfish were ~~are~~ present: Analamanga, Mahatsiatra Ambony and Ihorombe (see S1 Figure for details of interview locations in each region). ~~In each region w~~We first visited officials ~~the regional authorities~~in each region (Direction Regionale de l’Environnement, de l’Ecologie et des Forets, DREEF), as well as local authorities in each town or village where we worked (the commune or fokontany representative) to gain ~~local~~ permission~~ for the work~~.

We collected three ~~main~~ types of data: ~~(~~(1) At each location, enumerators ~~We~~ administered ~~carried out~~ a brief survey to ~~questionnaire survey targeted at~~ adults at markets, fields, streets and villages, or those working near water bodies ~~-using in the region designed to collect quantitative information. The questionnaire contains closed and Likert response questions.~~ convenience s~~S~~ampling. ~~was opportunistic and we did not use a sampling frame. In each location, enumerators approached adults in markets, in fields, streets and villages, or working in and around water bodies.~~ The questionnaire consisted of closed-ended items and took approximately 15 minutes to complete. ~~(~~(2) Additional open-ended questions were asked to individuals who were ~~For participants who were identified as~~ key informants (those involved in selling crayfish, crayfish harvesting, or having more knowledge on marbled crayfish)~~, we asked additional open-ended questions~~. These in-depth surveys lasted 30-45 minutes. ~~(~~(3) Finally, we measured the price per kilogram of processed marbled crayfish and other common sources of dietary protein at ~~also conducted a study of~~ 30 markets (containing more than 200 stalls) in Antananarivo.~~, where we recorded the price of processed marbled crayfish and other common sources of dietary protein as price per kilogram.~~

For each respondent, we ~~first clearly~~ introduced ourselves and ~~carefully~~ explained the aim of our study, ~~and~~ how the data would ~~will~~ be used, and provided assurances of confidentiality (~~. We explained that we are~~ not taking ~~any individually~~ identifiable ~~ying~~ information or recording voices). Due to the low level of literacy, oral consent was obtained for participation ~~from each respondent to voluntarily participate~~ in the survey, including permission ~~. Oral consent was also obtained for all~~ to photographs ~~taken~~. Interviews were conducted ~~carried out~~ by R.A., J.R., H.R. and M.R. with the help of trained enumerators. All interviewers were native speakers of Malagasy, comfortable in the dialects of the regions visited. Before starting an interview, each informant was shown a series of 16 photographs with crustacean and fish species to assess their knowledge of marbled crayfish (S2 Figure). Surveys were delivered using Open Data Kit (ODK)-Collect, an Android open source app for data collection. Data were submitted to ODK Aggregate when an internet signal was available.

We assessed the perception of marbled crayfish impacts by collecting information on the respondents' socio-economic status and their preferences. Next~~First~~, we ~~assessed the preference among the most common sources of protein in local diets. We~~ showed a series

Commented [MM2]: A similar statement is made later in the paper. Both should be combined and placed into the methods section.

of 16 photographs (S3 Figure) that illustrated~~ing~~ different sources of animal and plant-~~-~~based proteins ~~sources~~ and asked ~~the~~ respondents to rank order them by ~~in order of~~ preference, before recording the first five. We then collected information on their primary and secondary livelihoods (agriculture,

government position, private monthly waged work, daily waged labor, trader, wild product harvester, other). We also recorded whether people owned irrigated rice fields, and whether they fished commercially, for ~~either income or~~ subsistence, or ~~for~~ recreation. We investigated the perception of marbled crayfish impacts (the dependent variable) on six different aspects (rice farming, fishing, household economy, food security, animal feed, health) using a 5-point Likert scale illustrated by Emojis (very positive to very negative, coded 1-5, respectively). ~~bad, bad, neutral, good, very good).~~ The full survey (~~in~~ English and Malagasy) is available in the s~~S~~upporting i~~I~~nformation.

~~169~~

Data analysis

Data analysis was carried out using R3.4.3. Results from the closed-ended questions ~~Likert scales~~ were illustrated as diverging stacked bar charts using the *ggplot2* package. We also explored the relationships between marbled crayfish overall impact perception and potential predictors by fitting a multivariate ordinary least squares (OLS) regression. Our model investigated which factors predict whether the overall perception of marbled crayfish impact will tend to be more positive or negative. ~~The dependent variable (the overall perception of marbled crayfish impacts) is measured on a 5-point ordinal scale: 1 being very positive and 5 very negative.~~ Eight variables were considered as potential predictors: survey location, gender, age, livelihood, relationship to marbled crayfish (harvester, trader, buyer), and the use of marbled crayfish as animal feed. All variables except age were ~~are~~ categorical and were transformed into dummy variables before running the regression.

For sensitivity analysis, we also ran an ordinal regression (function *polr* from R package MASS) with the same predictors, given the level of measurement of the dependent variable, and found similar results to the OLS regression (S1 Table). We present the results of OLS regression for ease of interpretability of coefficient and model fit. Finally, we evaluated the prediction model accuracy by running a k-cross validation analysis (10-fold) followed by a repeated k-cross-validation, using the functions *cv* and *repeatedcv* from the R package CARET, with a seed set on 523 for the reproducibility of the analysis (S2 Table).

Commented [MM3]: Before I combined the sections, you said that the dependent variable was measured on a 5-point ordinal scale. How do you know it was ordinal, instead of interval? Did you test for homogeneity of variances? Your decision will influence the statistics.

Commented [MM4]: Not a true statement, see above.

189174 189

Results

Compared to a ~~previous~~ survey conducted in ~~from~~ 2017 [28] we did not observe ~~record~~ a range ~~n~~ expansion of the marbled crayfish ~~known~~ ~~range~~ in Madagascar after ~~during this survey (March and April 2019). R~~re-sampling at 18 previously negative sites in the Analanjirofo, Menabe and Vatovavy Fitovinany regions ~~revealed no marbled crayfish~~ (Fig. 1A). However, marbled crayfish were discovered at 15 additional sites in the Analamanga, Mahatsiatra Ambony and Ihorombe regions (i.e., within the previously identified range) during the present survey (Fig. 1A). The high density of marbled crayfish populations in these regions likely reflects the continuing anthropogenic distribution due to commercial activity~~exploitation~~ [31].

We conducted ~~carried out~~ social surveys in three regions (Fig. 1A): Analamanga (mostly in Antananarivo at~~in~~ 12 sampling locations~~points~~), Mahatsiatra Ambony (mostly in Fianarantsoa at~~in~~ 8 sampling locations~~points~~) and Ihorombe (mostly in Ihosy at ~~in~~ 3 sampling locations~~points~~). Using convenience ~~We used haphazard~~ sampling focused on ~~targeting~~ the adult population, we ~~and~~ approached 435 persons in total (Fig. 1B, S1 Figure). We obtained good response ~~participation~~ rates (>86%) in all three regions (Fig.1B), with sample sizes of 142, 143, and 100 ~~participants~~ in Analamanga, Mahatsiatra Ambony and Ihorombe, respectively. The average participant age of 39 years, and a sex ratio of 54% (male) versus 46% (female). The most commonly reported primary livelihood among respondents was farming~~er~~ (48%).

Commented [MM5]: Most, if not all, of this information can go under methods. It is acceptable to report some results there, such as response rate.

Fig. 1. Distribution of marbled crayfish in Madagascar. (A) Map of Madagascar. Light green dots indicate sites with known marbled crayfish populations from 2017 [28]. Dark green dots indicate additional sites where marbled crayfish were detected during this study (in 2019), light blue dots indicate 3 re-sampled negative sites from 2017 with no marbled crayfish. The three regions where the social surveys presented in this paper were carried out are highlighted by color. (B) Participant response rates and numbers of survey respondents per

region. (C) Earliest observation of marbled crayfish in the three regions. Numbers indicate % respondents.

A subset of respondents (n=275) were asked when they first observed marbled crayfish in their local area. In the Analamanga region, a substantial fraction of respondents indicated that they became first aware of marbled crayfish before 2005 and the animals became well-known by 2010 (Fig. 1C). In contrast, marbled crayfish were mostly unknown in Mahatsiatra Ambony and completely unknown in Ihorombe before 2011 (Fig. 1C). This is consistent with a spread of the animals from the center to the south of Madagascar.

> **Commented [MM6]:** Contradiction. Maybe percent is a better word.

~~To explore the perception of marbled crayfish impacts, we used a Likert scale to investigate six variables.~~

Perceptions of marbled crayfish impacts

~~The results show that attitudes towards marbled crayfish~~ varied~~y~~ considerably ~~substantially~~ by region. Generally speaking, r~~ with r~~espondents in the two southern regions (Mahasiatra Ambony and Ihorombe) ha~~dving~~ ~~generally~~ more negative attitudes toward~~s~~ the crayfish, while those in Antananarivo were ~~generally~~ more positive (Fig. 2). However, in all three regions, respondents reported both ~~some~~ positive and negative impacts of the marbled crayfish ~~and some negative impacts~~. Negative impacts on rice farming and fishing were reported at all sites (and by nearly 100% of respondents in Ihorombe), while contribution to food security and household economy was generally seen as positive.

Fig. 2. Perceived socio-economic impacts of marbled crayfish in 3 regions of Madagascar. Bar plots show overall impacts (Likert scales) in the three regions.

~~The r~~Responses to the open ~~-~~ended questions by ~~in the~~ key informants ~~interviews~~ provided some useful ~~qualitative~~ information to complement ~~support and explain~~ the quantitative data. The reasons for widely reported negative impacts of marbled crayfish on rice fields was due to burrowing activities which evaporate ~~dry~~ the rice fields and require the farmers to ~~regularly~~ repair their banks and irrigation canals regularly. This results in more work for the farmer and can lead to disagreements between rice field owners. For example: "the marbled crayfish destroy the mud walls we build around our

rice fields" (S4 Table, Q1-3). The occasional positive comments about the impacts of marbled crayfish on rice farming came comes from the notion perception that marbled crayfish burrowing can improve rice production by loosening and aerating the soil (S4 Table, Q4). The perceived impacts of marbled crayfish on fishing were overwhelmingly negative, because the crayfish are known to the predate on young fish, though not larger onesfish (S4 Table, Q5). Importantly, some impacts may not be directly observed after an initial marbled crayfish colonization: "the impacts on fishing can't really be seen since the marbled crayfish are present for only two years" (S4 Table, Q6). Many people mentioned positive impacts from crayfish harvesting on local livelihoods from catching and selling crayfish, or using them as a cheap source of household protein or animal feed). A Fokontany worker in a village with several households depending on marbled crayfish described marbled crayfish as "a small animal coming from who knows where which supports the needs of the family" (S4 Table, Q7). Similarly, a farmer reported that marbled crayfish had given him a good living since they arrived in the area about 10 years ago (S4 Table, Q8). Marbled crayfish were often mentioned as a good and cheap/free replacement for meat, and a source of energy and strength (S4 Table, Q9). However, some individuals reported respondents have also observed allergic reactions, and stomach pain due to the consumption of marbled crayfish (S4 Table, Q10).

In order tTo explore link the overall perception of marbled crayfish impact to socio-economic characteristics of respondents, we selected eight variables as predictors in a multivariate linear model. Our model explores the relationship between these eight variables and overall perception towards marbled crayfish (Fig. 3, S3 Table) showed a good overall fit, explaining : the model successfully explains 44% of the variance tion of the dependent variable (adjusted R--squared of 0.438). Additionally, the 10--fold cross -validation of our regression analysis revealed a good performance with an R-squared of 0.42. Although the A positive overall attitude towards the marbled crayfish was positive, it was a bi-modal relationship. Individuals is associated with study site, as participants in Analamanga showed a significant positive association (+0.95 scale points) whereas people in and Ihosy had a significant negative association (-0.44 scale points) as compared to those in Fianarantsoa (controlling for gender, age and livelihood). In other words, tThose involved in crayfish harvesting were are more positive towards marbled crayfish than non-

harvesters (+0.67 scale points). Also, tThe same scenario picture was observed for crayfish customers and those using the marbled crayfish as animal feed with respectively (0.46 and 0.27 scale points more positive, as compared to those who do not purchase buy or and use marbled crayfish). Finally, livelihoods are is significantly associated with the overall perception with traders (and our grouping of 'other' livelihoods) having a more positive average scores as compared to farmers. This is perhaps unsurprising as fFarmers will suffer from the negative impacts associated with the damage that marbled crayfish inflicts do to on rice fields, while traders are more likely to profit benefit from selling crayfish.

Fig. 3. Coefficient plot of the model exploring the relationship between overall perception of crayfish and 8 socio-economic predictors. Blue bars represent the coefficients for locations while yellow bars shows the coefficients for livelihoods, black bars illustrate the coefficients crayfish-related activities and green bars the coefficient for demographic controls. Error bars represent 95% confidence intervals. Gender: male and aAge were included as controls. The intercept was defined by the following parameters: location: Mahatsiatra Ambony; livelihood: farmer, not harvesting crayfish, not selling crayfish, not buying crayfish, not using crayfish as animal feed.

Respondents were asked to identify and rank 16 common examples of proteins to determine the relative position of To assess the popularity of marbled crayfish in the diet of our survey participants, we asked them to identify and rank their five most preferred sources of nutritional proteins among 16 photographs of dietary protein sources provided in their diet (S3 Figure). The rResults showed that tilapia fish and chicken (sold live) were represent the most preferred sources of dietary protein (Fig. 4), which is consistent with previous findings [32,33]. Marbled crayfish meat was less preferred and grouped together with shrimps, factory produced chicken, meat and beef liver (Fig. 4).

Other Several other sources of dietary proteins sources, such as tripe, beans and crabs, were less preferred than marbled crayfish.

Fig. 4. Preferences and relative price for kilo of a selection of common sources of dietary protein. Photographs and descriptions are provided in S2 Figure. Left panel: results of preference ranking for sources of dietary protein. Right panel: mean of market prices per kilo for each dietary protein source (n=number of stalls where prices were recorded in the 30 Antananarivo markets visited). Error bars represent 95% confidence intervals. For comparison, the price of live marbled crayfish from vendors in Ihorombe (1,200 MGA/kg) is also indicated.

In Antananarivo (Analamanga region), marbled crayfish were ~~mainly~~ sold mainly in markets (Fig. 5A) and their leftover carapaces were used as animal feed (Fig. 5B). We found marbled crayfish in 33 stalls in 11 markets (out of 30 markets investigated). They were mainly sold as boiled tail meat, the average price per kg was 8,600 MGA (approximately 2 EUR, also see Fig. 3). This strongly contrasts with Ihosy (Ihorombe region), where live marbled crayfish were~~are~~ ~~predominantly~~ sold ~~live~~ in large bags for widespread distribution (Fig. 5C). The marbled crayfish business is structured differently in Ihosy, as compared to Antananarivo: orders were ~~are~~ received from different locations and regions. Traders then purchased live marbled crayfish from local collectors in~~to~~ respon~~sed~~ to ~~the~~ market demand. Up to 20 bags of 35 to 80 kg with live marbled crayfish were ~~are~~ transported daily using ~~with short and long distance~~ "taxi-brousse" ~~for a fixed fee per bag~~ during the rainy season (November to March) for a fixed fee, per bag. The price of live marbled crayfish was~~is~~ much lower with an average of 1,200 MGA/kg (approximately 0.3 EUR).

Fig. 5. Commercial sales and distribution of marbled crayfish. (A) Marbled crayfish meat sold with freshwater fish. (B) Leftover shells from marbled crayfish used as animal feed. (C) Bag of live marbled crayfish for distribution. (D) Live and boiled marbled crayfish sold on the street. (E) Boiled and salted marbled crayfish sold in the market. (F) Ready-to-eat marbled crayfish sold in street food markets.

Interestingly, Fianarantsoa (Mahatsiatra Ambony region) was mentioned as one of the regions ordering from Ihosy. This was confirmed by interviews of marbled crayfish vendors in Fianarantsoa's evening market who indicated that they order marbled crayfish from Ihosy, and usually sell their complete order within a few hours (Fig. 5D). In Fianarantsoa, marbled crayfish were usually sold as processed food (Fig. 5E). Marbled crayfish beignets and meals with marbled crayfish in tomato and green onion sauce (Fig. 5F) were offered in several street food locations and local ~~street market~~ restaurants. ~~Interestingly, d~~Despite a~~n official~~ prohibition from the Ministry of Agriculture, Livestock and Fisheries (effective since 2009) that forbids the transport of live marbled crayfish, the transport of marbled crayfish (for example by taxi brousse), is supported by regular permits and fees. We interviewed taxi-brousse drivers from different transport companies in Ihorombe, who stated that those who transport marbled crayfish pay for a yearly permit, in the same way they need a permit to transport other freshwater products.

Discussion

~~It is now indisputable that w~~We live in the Anthropocene; a geological age defined by human~~ity's~~ impact on the environment [34]. Given ~~In~~ this situation, some ecologists are rethinking their response to invasive species, increasingly recognizing their ecological role, ~~that non-native species are a~~both now and into ~~part of the ecology of~~ the future [35,36]. While the strongly negative impacts of some invasive species render this view as controversial ~~view~~ [37], those involved in managing invasive species have pointed out that benefits from such species are often overlooked or under-reported [36]. Our results, which provide the first information on the socio-economic impact of a parthenogenetic decapod described as 'the perfect invader' [21], offers a compelling ~~provide a useful case~~ rationale for ~~study in how people~~ makinge use of an invasive ~~new~~ species ~~in their environment~~. It also provides information useful for predicting the likely future spread of this invasive crayfish in a country with freshwater biodiversity of global value [38].

Commented [MM7]: regions cannot order, only people in regions.

Commented [MM8]: It's not a good idea to use 'order' as a noun and verb in the same sentence.

Madagascar is one of the poorest countries in the world, with 75% of the population living on less than $1.90 per day [39,40]. Poverty manifests itself in limited intake of dietary protein leading to stunting [41] which affects 1 in 2 Malagasy children [42]. In fact, many people rarely consume animal protein [32,33]. Given the lack of sufficient protein for human consumption; livestock are mostly fed rice bran, roots and tubers [43], providing insufficient protein for production. New and cheap sources of animal protein therefore have the potential to have a significant positive impact. Positive perceptions of marbled crayfish (especially prevalent in the Analamanga region) are strongly associated with harvesting, buying or using marbled crayfish for animal feeds. The fact that the crayfish can be ~~quite easily~~ collected easily from public lakes, rivers, canals and rice fields also means it can be done ~~they can be collected at very low cost~~cheaply.

Live animals are sold at low prices (~~live~~ marbled crayfish are among the cheapest sources of animal protein available), which makes them affordable for poorer people. Leftover parts from processed marbled crayfish are fed to animals (mainly chicken and pigs); providing an additional source of income from those involved in the crayfish trade and providing a very effective, and relatively cheap, protein-rich livestock feed. In Antananarivo, where the species has been common for at least 10 years [21], the market has evolved and mostly peeled tail meat is sold. This value-added product (similar to processed fish and pre-cut vegetables) increases the profit available from the crayfish. A different market exists in Ihorombe, where the animals appeared considerably later and most ~~trade is of~~ live crayfish trade is done in bulk. While marbled crayfish are seldom among respondent's most preferred food, they are ranked similarly to shrimp~~s~~ and factory processed chicken, ~~and~~ thus representing an acceptable source of dietary protein.

The most significant negative impacts reported were on rice farming and fishing. A related species of crayfish (*Procambarus clarkii*) is known to ~~negatively~~ disturb ~~affect~~ irrigated rice production in the ~~Iberian peninsula~~Iberian Peninsula, since ~~where~~ it damages banks and irrigation canals by burrowing [44,45]. However, the extent to which rice production is impacted by marbled crayfish in Madagascar remains poorly understood and our ~~survey~~ results, ~~and associated~~

qualitative information, provided yielded somewhat contradictory evidence. InMost of the participants in - Analamanga were neutral about the invasive crayfish (where

they ~~the crayfish~~ have been present the longest), ~~the largest proportion of respondents reported no impact, while~~ but others ~~some~~ reported a positive impact, perhaps due ~~because of the impact of~~ the benefit of ~~burrowing crayfish on~~ soil aeration. It is possible that ~~reported~~ the negative impacts of crayfish stem from ~~the~~ extensive media reporting of their harmful effects ~~possible negative impacts~~ on rice, rather than firsthand experience. ~~The a~~Available evidence suggests negative impacts on fishing, but further research is ~~urgently~~ needed to understand the effects ~~impacts~~ on fish~~ing~~ and freshwater ecosystems.

The overall perception of marbled crayfish impacts ~~was significantly~~ differed significantly ~~nt~~ across ~~in~~ the three regions that were surveyed. Participants in the Analamanga region perceived crayfish more favorably than participants in the Mahatsiatra Ambony and Ihorombe regions. This may be due to differences in livelihoods. ~~of our sample population in the three regions.~~ Those interviewed in the Mahatsiatra Ambony and Ihorombe regions reliedy more on farming, especially ~~particularly~~ rice production. ~~farming, and perceived n~~Negative impacts on rice farming are likely to ~~strongly~~ influence ~~the~~ overall ~~negative~~ perception. Another possible explanation is that people in Analamanga ~~(where marbled crayfish have been established the longest; since about 2005)~~ ~~people~~ have adapted ~~had more time to get used~~ to the species, due to 'its relative longevity. ~~presence and adapt to the opportunities it presents.~~

The geographical range of many invasive species is driven by socio-economic factors, such as trade, travel and the movement of goods [46,47]. Marbled crayfish were already sold in markets in Madagascar soon after they appeared. This raised the possibility that their potential economic importance could promote their spread [21]. Our work clearly confirms that the market demand for the crayfish is likely to encourage spread into new areas. Even in Ihorombe, where the overall perception of the crayfish is negative, the strong market demand and widespread distribution of live animals are likely to favor ~~further~~ spread in the future.

Some ~~It is also important to point out the~~ limitations of our analysis include: ~~. The opportunistic~~ the sampling approach used~~,~~ and the relatively small numbers of interviews. This ~~,~~ means that our sample is not fully representative of the population in each region. Given the resources and

time available, probabilistic sampling was not possible. However, we sampled several locations ~~points~~

in each region (see maps in S1 Figure) and the high response rate means that the responses are not likely to be biased. Furthermore, we controlled for differences in age and gender.

The spread of the marbled crayfish in Madagascar over the last decade has been nothing short of dramatic. Given the practical challenges to eradication of invasive species, a balanced approach to considering the costs and of benefits arising from invasiveness is needed [36]. There are differing overall perceptions towards marbled crayfish both between regions and between people with different socio-economic characteristics. However, it is clear that the market for marbled crayfish, and its widespread consumption, is likely to result in further spread. It is difficult to imagine any inhabitable parts of Madagascar that will remain uninvaded over the next decade. What this will mean for Madagascar's freshwater biodiversity is still completely unknown. This study has attempted to provide some insight into what impact the invasion has on local people's livelihoods. An important area of uncertainty remains the impact of marbled crayfish on rice agriculture. Given the importance of irrigated rice production to the economy of Madagascar, this is certainly worthy of further investigation. What this study has clearly demonstrated is that, since their appearance in Madagascar in the mid 2000s, marbled crayfish have rapidly become a widely consumed, cheap source of animal protein in local diets. Given the desperate need for protein in the diets of many Malagasy people, this can be considered a positive development.

Acknowledgements

We thank Maevatiana N. Ratsimbazafindranahaka and Luticia V. Raharimalala for their valuable support of our survey, and Amy Lewis for help with coding the survey in Open Data Kit. We also thank Soatiana Daniel and Laza S. Andriantsoa for their help with field trip management.

References

1. McNeely JA, Mooney HA, Neville LE, Schei PJ W. A Global Strategy on Invasive Alien Species. IUCN, Gland, Switzerland and Cambridge, UK.; 2001.
2. Pyšek P, Richardson DM. Invasive Species, Environmental Change and Management, and Health. Annu Rev Environ Resour. 2010;35: 25–55. doi:10.1146/annurev-environ-033009-095548
3. Giuseppe M, Elena T. Invasive species and human health. Invasive species and human health. CAB International, Wallingford, UK.; 2018. doi:10.1079/9781786390981.0000
4. Hejda M, Pyšek P, Jarošík V. Impact of invasive plants on the species richness, diversity and composition of invaded communities. J Ecol. 2009;97: 393–403. doi:10.1111/j.1365-2745.2009.01480.x
5. Seeney A, Eastwood S, Pattison Z, Willby NJ, Bull CD. All change at the water's edge: invasion by non-native riparian plants negatively impacts terrestrial invertebrates. Biol Invasions. 2019;21: 1933–1946. doi:10.1007/s10530-019-01947-5
6. Clavero M, García-Berthou E. Invasive species are a leading cause of animal extinctions. Trends Ecol Evol. 2005;20: 110. doi:10.1016/j.tree.2005.01.003
7. Hoffmann BD, Broadhurst LM. The economic cost of managing invasive species in Australia. NeoBiota. 2016;31: 1–18. doi:10.3897/neobiota.31.6960
8. Bellard C, Genovesi P, Jeschke JM. Global patterns in threats to vertebrates by biological invasions. Proc R Soc B Biol Sci. 2016;283: 1–9. doi:10.1098/rspb.2015.2454
9. Rogers P, Nunan F, Fentie AA. Reimagining invasions: The social and cultural impacts of Prosopis on pastoralists in southern Afar, Ethiopia. Pastoralism. 2017;7: 1–13. doi:10.1186/s13570-017-0094-0
10. Ngorima A, Shackleton CM. Livelihood benefits and costs from an invasive alien tree (Acacia dealbata) to rural communities in the Eastern Cape, South Africa. J Environ Manage. 2019;229: 158–165. doi:10.1016/j.jenvman.2018.05.077

11. Shackleton RT, Shackleton CM, Kull CA. The role of invasive alien species in shaping local livelihoods and human well-being: A review. J Environ Manage. 2019;229: 145–157. doi:10.1016/j.jenvman.2018.05.007

12. Bobeldyk AM, Lamberti GA. A Decade after Invasion: Evaluating the Continuing Effects of Rusty Crayfish on a Michigan River. J Great Lakes Res. 2008;34: 265–275. doi:10.3394/0380-1330(2008)34[265:ADAIET]2.0.CO;2

13. Jernelöv A. Crayfish Plague in Europe. The Long-Term fate of Invasive Species. Springer International Publishing; 2017. pp. 91–103. doi:10.1007/978-3-319-55396-2_7

14. Food and Agriculture Organisation of the United Nations (FAO). Cultured aquatic fact sheets: Procambarus clarkii. 2009 [cited 6 Nov 2019]. Available: http://www.fao.org/fishery/culturedspecies/Procambarus_clarkii/en

15. Wang Q, Ding H, Tao Z, Ma D. Crayfish (Procambarus clarkii) Cultivation in China: A Decade of Unprecedented Development. Aquaculture in China: Success Stories and Modern Trends. John Wiley & Sons Limited; 2018. pp. 363–377. doi:10.1002/9781119120759.ch4_1

16. Pienkowski T, Williams S, McLaren K, Wilson B, Hockley N. Alien invasions and livelihoods: Economic benefits of invasive Australian Red Claw crayfish in Jamaica. Ecol Econ. 2015;112: 68–77. doi:10.1016/J.ECOLECON.2015.02.012

17. Mkoji GM, Hofkin B V., Kuris AM, Stewart-Oaten A, Mungai BN, Kihara JH, et al. Impact of the crayfish Procambarus clarkii on Schistosoma haematobium transmission in Kenya. Am J Trop Med Hyg. 1999;61: 751–759. doi:10.4269/ajtmh.1999.61.751

18. Lyko F. The marbled crayfish (Decapoda: Cambaridae) represents an independent new species. Zootaxa. 2017;4363: 544–552. doi:10.11646/zootaxa.4363.4.6

19. Scholtz G, Braband A, Tolley L, Reimann A, Mittmann B, Lukhaup C, et al. Parthenogenesis in an outsider crayfish. Nature. 2003;421: 806. doi:10.1038/421806a

20. Feria TP, Faulkes Z. Forecasting the distribution of Marmorkrebs, a parthenogenetic

crayfish with high invasive potential, in Madagascar, Europe, and North America. Aquat Invasions. 2011;6: 55–67. doi:10.3391/ai.2011.6.1.07

21. Jones JPG, Rasamy JR, Harvey A, Toon A, Oidtmann B, Randrianarison MH, et al. The perfect invader: A parthenogenic crayfish poses a new threat to Madagascar's freshwater biodiversity. Biol Invasions. 2009;11: 1475–1482. doi:10.1007/s10530-008-9334-y

22. Lokkös A, Müller T, Kovács K, Várkonyi L, Specziár A, Martin P. The alien, parthenogenetic marbled crayfish (Decapoda: Cambaridae) is entering Kis-Balaton (Hungary), one of Europe's most important wetland biotopes. Knowl Manag Aquat Ecosyst. 2016;16: 1–9. doi:10.1051/kmae/2016003

23. Patoka J, Buřič M, Kolář V, Bláha M, Petrtýl M, Franta P, et al. Predictions of marbled crayfish establishment in conurbations fulfilled: Evidences from the Czech Republic. Biologia (Bratisl). 2016;71: 1380–1385. doi:10.1515/biolog-2016-0164

24. Pârvulescu L, Togor A, Lele S-F, Scheu S, Șinca D, Panteleit J. First established population of marbled crayfish Procambarus fallax (Hagen, 1870) f. virginalis (Decapoda, Cambaridae) in Romania. BioInvasions Rec. 2017;6: 357–362. doi:10.3391/bir.2017.6.4.09

25. Deidun A, Sciberras A, Formosa J, Zava B, Insacco G, Corsini-Foka M, et al. Invasion by non-indigenous freshwater decapods of Malta and Sicily, central Mediterranean Sea. J Crustac Biol. 2018;076: 1–6. doi:10.1093/jcbiol/ruy076

26. Lipták B, Mrugała A, Pekárik L, Mutkovič A, Grul'a D, Petrusek A, et al. Expansion of the marbled crayfish in Slovakia: Beginning of an invasion in the Danube catchment? J Limnol. 2016;75: 305–312. doi:10.4081/jlimnol.2016.1313

27. Kawai T, Scholtz G, Morioka S, Ramanamandimby F, Lukhaup C, Hanamura Y. Parthenogenetic Alien Crayfish (Decapoda: Cambaridae) Spreading in Madagascar. J Crustac Biol. 2009;29: 562–567. doi:10.1651/08-3125.1

28. Gutekunst J, Andriantsoa R, Falckenhayn C, Hanna K, Stein W, Rasamy J, et al. Clonal genome evolution and rapid invasive spread of the marbled crayfish. Nat Ecol

Evol. 2018;2: 567–573. doi:10.1038/s41559-018-0467-9

29. Jones JPG, Andriahajaina FB, Hockley NJ, Crandall KA, Ravoahangimalala OR. The ecology and conservation status of Madagascar's endemic freshwater crayfish (Parastacidae; Astacoides). Freshw Biol. 2007;52: 1820–1833. doi:10.1111/j.1365-2427.2007.01766.x

30. Boyko CB, Ravoahangimalala OR, Randriamasimanana D, Razafindrazaka TH. Astacoides hobbsi, a new crayfish (Crustacea: Decapoda: Parastacidae) from Madagascar. Zootaxa. 2005; 41–51. doi:10.11646/zootaxa.1091.1.3

31. Andriantsoa R, Tönges S, Panteleit J, Theissinger K, Carneiro VC, Rasamy J, et al. Ecological plasticity and commercial impact of invasive marbled crayfish populations in Madagascar. BMC Ecol. 2019;19: 1–10. doi:10.1186/s12898-019-0224-1

32. Jenkins RKB, Keane A, Rakotoarivelo AR, Rakotomboavonjy V, Randrianandrianina FH, Razafimanahaka HJ, et al. Analysis of patterns of bushmeat consumption reveals extensive exploitation of protected species in eastern madagascar. PLoS One. 2011;6: 12p. doi:10.1371/journal.pone.0027570

33. Kurien J, López Ríos J. Flavouring Fish into Food Security. FAO SmartFish Publ. 2013.

34. Lewis SL, Maslin MA. Defining the Anthropocene. Nature. 2015;519: 171–180. doi:10.1038/nature14258

35. Carroll SP. Conciliation biology: The eco-evolutionary management of permanently invaded biotic systems. Evol Appl. 2011;4: 184–199. doi:10.1111/j.1752-4571.2010.00180.x

36. Bonanno G. Alien species: to remove or not to remove? That is the question. Environ Sci Policy. 2016;59: 67–73. doi:https://doi.org/10.1016/j.envsci.2016.02.011

37. Russell JC, Blackburn TM. The Rise of Invasive Species Denialism. Trends Ecol Evol. 2017;32: 3–6. doi:https://doi.org/10.1016/j.tree.2016.10.012

38. Benstead JP, Rham PH De, Gattolliat J, Loiselle P V, Sartori M, Sparks JS, et al. Conserving Madagascar's Freshwater Biodiversity. Sci York. 2009;53: 1101–1111.

doi:10.1641/0006-3568(2003)053[1101:CMFB]2.0.CO;2

39. World Bank; Macro Povery Outlook. Madagascar. 2016. Available: http://pubdocs.worldbank.org/en/615781477329260085/mpo-am16-mdg.pdf
40. World Bank. Madagascar Country Overview. 2019 [cited 18 Sep 2019]. Available: https://www.worldbank.org/en/country/madagascar/overview
41. Semba RD. The rise and fall of protein malnutrition in global health. Ann Nutr Metab. 2016;69: 79–88. doi:10.1159/000449175
42. International Food Policy Research Centre. Global Hunger Index: Madagascar. 2018. Available: https://www.globalhungerindex.org/madagascar.html
43. Food and Agriculture Organisation of the United Nations (FAO) and Livestock Information Sector Analysis and Policy Branch (AGAL). Madagascar Livestock sector brief. FAO. 2005.
44. Anastácio PM, Marques JC. Crayfish, Procambarus clarkii, effects on initial stages of rice growth in the lower Mondego River valley (Portugal). Freshw Crayfish. 1997;11: 608–617.
45. Anastácio PM, Soares M, Correia AM. Crayfish (Procambarus clarkii) consumption of wet-seeded rice plants (Oryza sativa): modifications throughout the rice growing period . SIL Proceedings, 1922-2010. 2005;29: 849–851. doi:10.1080/03680770.2005.11902800
46. Tabak MA, Piaggio AJ, Miller RS, Sweitzer RA, Ernest HB. Anthropogenic factors predict movement of an invasive species. Ecosphere. 2017;8: 1–13. doi:10.1002/ecs2.1844
47. Hulme PE. Invasion pathways at a crossroad: Policy and research challenges for managing alien species introductions. J Appl Ecol. 2015;52: 1418–1424. doi:10.1111/1365-2664.12470

Supporting information captions

S1 Figure. Map of survey locations.

S2 Figure. Pictures of crustaceans and fish for marbled crayfish identification.

S3 Figure. Pictures representing sources of dietary protein.

S1 Table. Statistical modelling of marbled crayfish perception.

S2 Table. Comparative analysis of linear and ordinal regression.

S3 Table. k-cross-validation analysis.

S4 Table. Quotes in English and in Malagasy languages.

Figure 1

Click here to access/download;Figure;Figure 1.tif

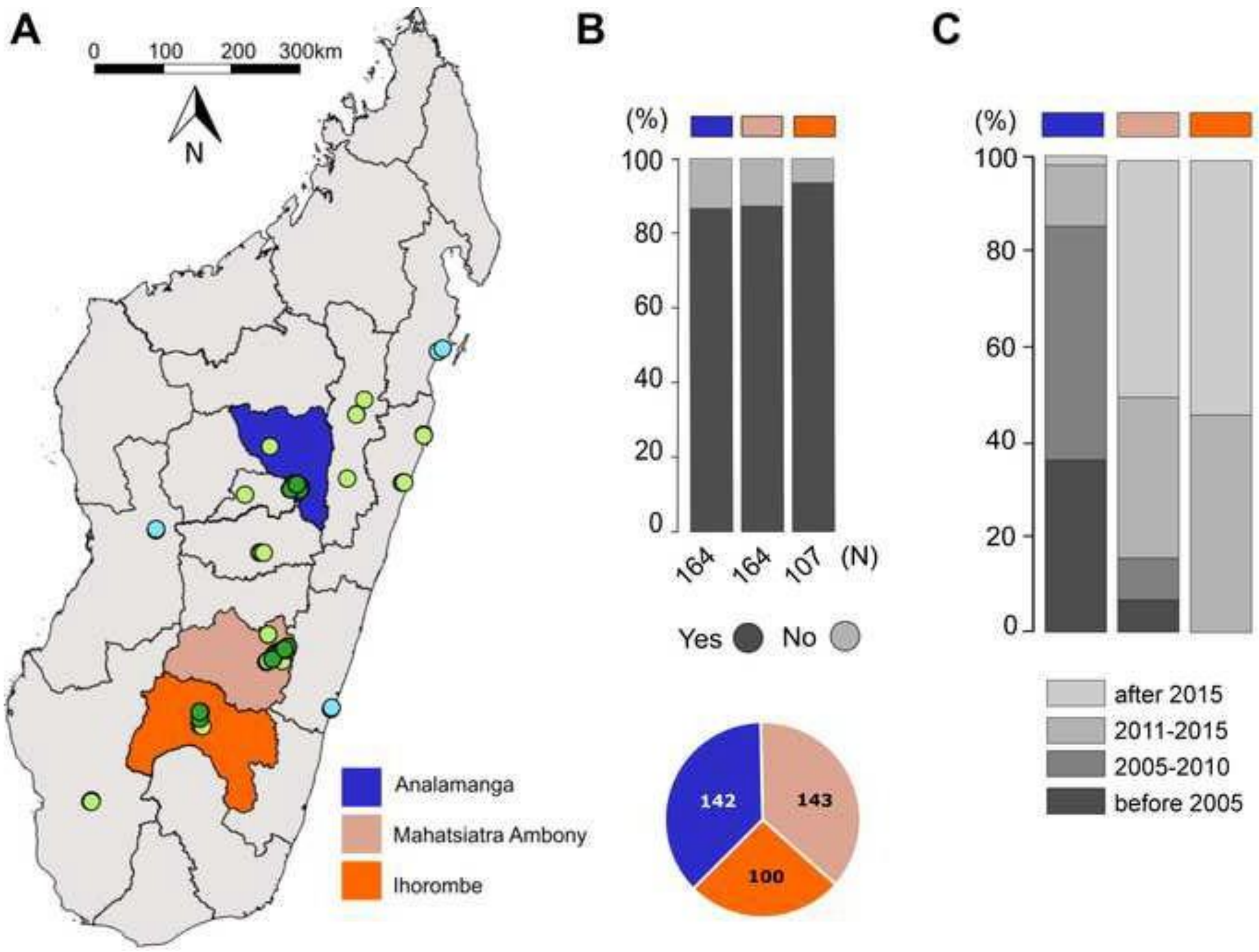

Figure 2

Click here to access/download;Figure;Figure 2.tif

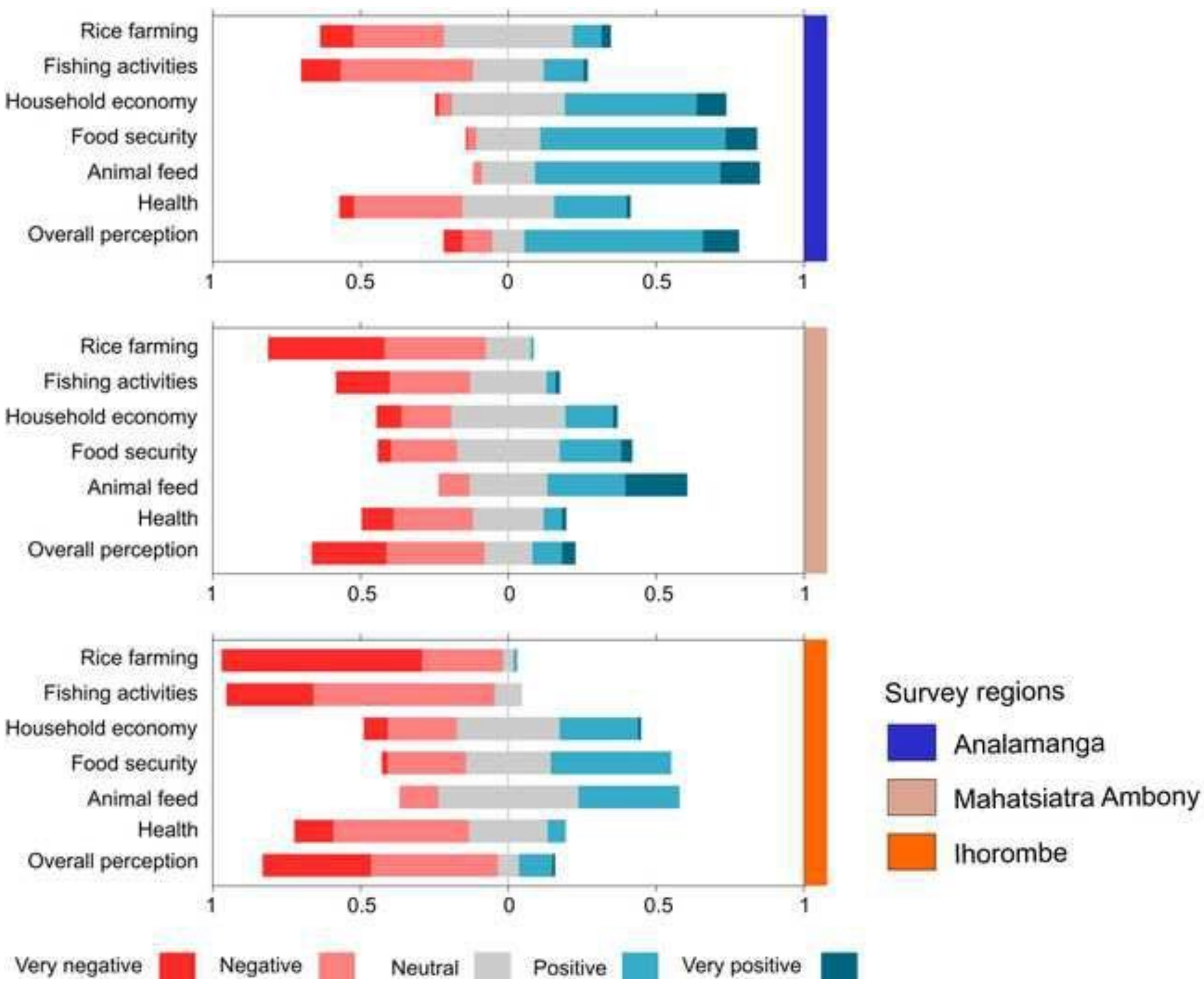

Figure 3

Click here to access/download;Figure;Figure 3.tif

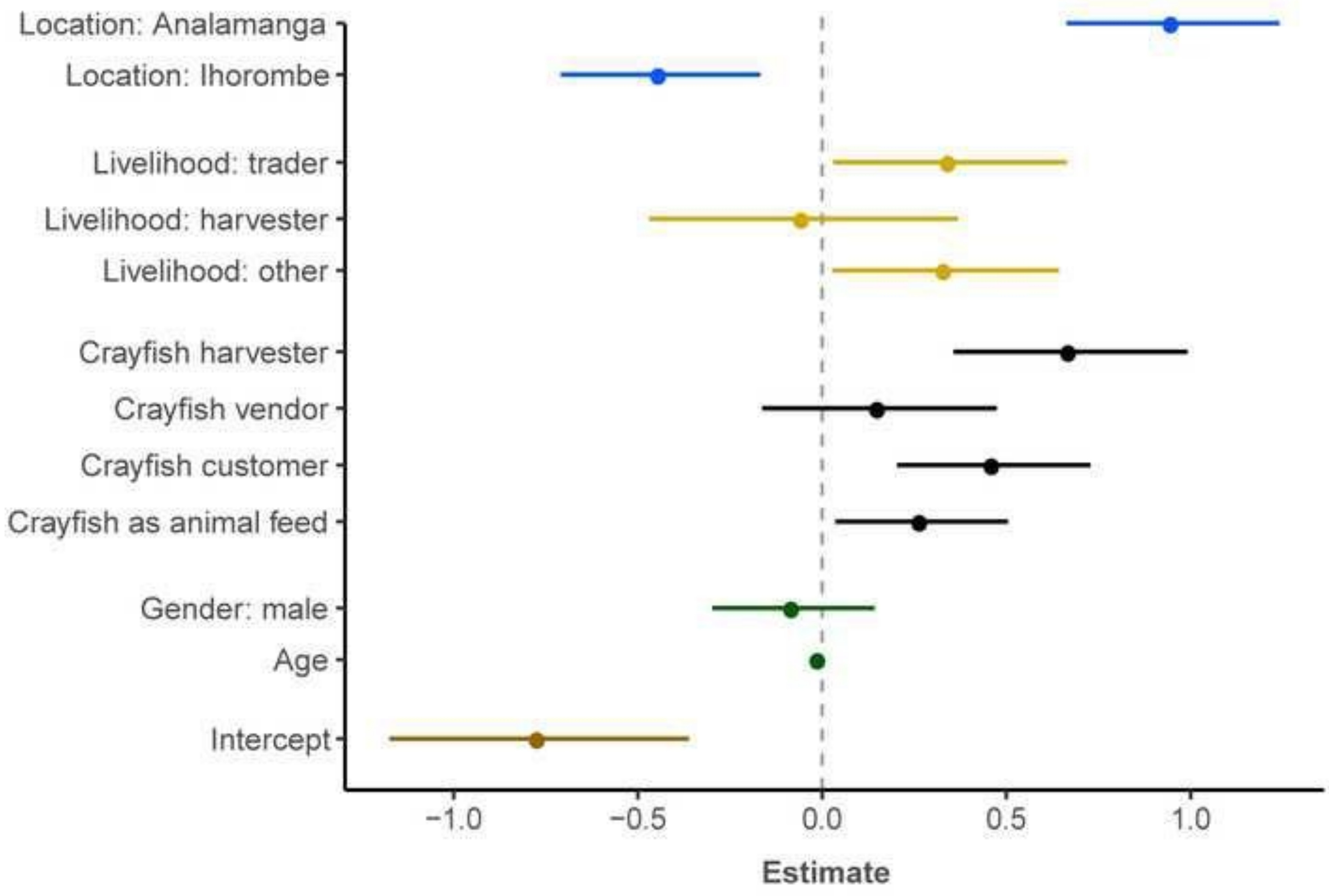

Figure 4

Click here to access/download;Figure;Figure 4.tif

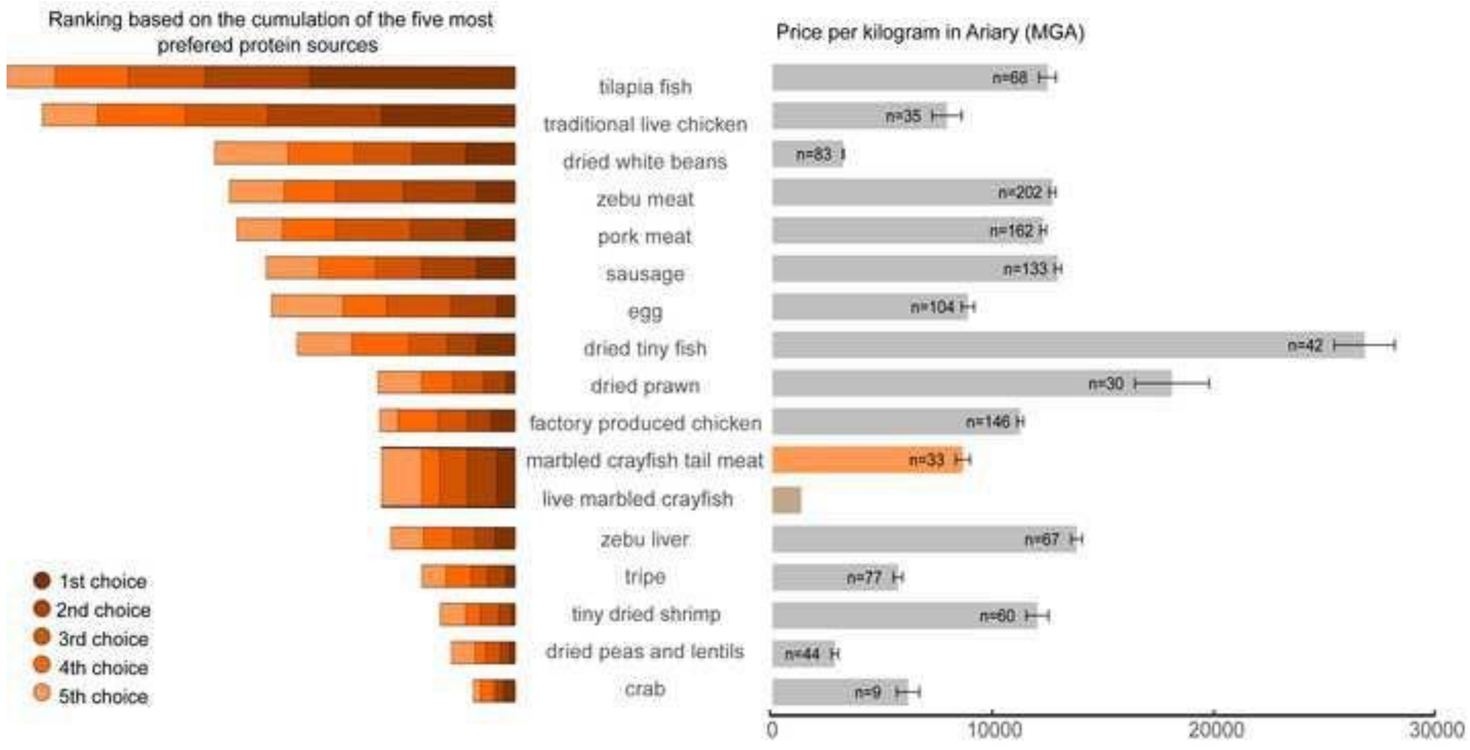

Figure 5

Click here to access/download;Figure;Figure 5.tif

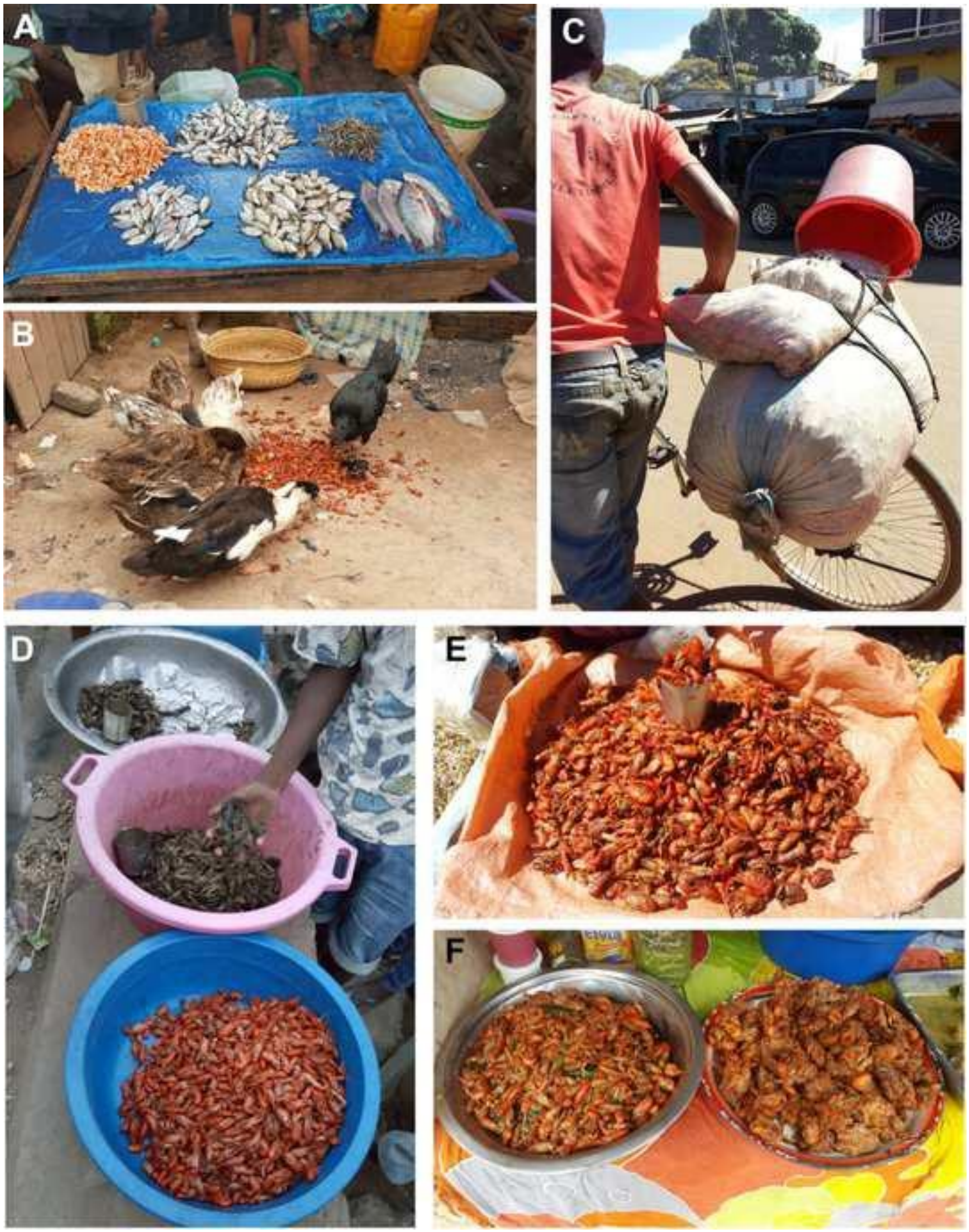

complete Supporting Information file

Click here to access/download
Supporting Information
SI_submitted.docx
