# Supplementary material for: Perceived socio-economic impacts of the marbled crayfish invasion in Madagascar
Source: PLoS One. 2020 Apr 15;15(4):e0231773. doi: 10.1371/journal.pone.0231773 (PMC7159205; doi:10.1371/journal.pone.0231773)
Supplement: Attachment — Submitted filename: PONE-D-19-30933_R1_edits.pdf [file pone.0231773.s006.pdf]

Manuscript Click here to access/download;Manuscript;ms_revised.docx



# Socio-economic impacts of the marbled crayfish invasion in Madagascar

Ranja Andriantsoa[1], Julia P.G. Jones[2], Vlad Achimescu[3], Heriniaina Randrianarison[4], Miary Raselimanana[4], Manjary Andriatsitohaina[4], Jeanne Rasamy[4], Frank Lyko[1*]

[1]Division of Epigenetics, DKFZ-ZMBH Alliance German Cancer Research Center (DKFZ) Heidelberg, Germany

[2]School of Environmental Sciences and Engineering, Bangor University, Bangor, United Kingdom

[3]School of Social Science, Mannheim University, Mannheim, Germany

[4]Mention Zoologie et Biodiversité Animale, Université d'Antananarivo,Antananarivo, Madagascar

*Corresponding author

Email: f.lyko@dkfz.de (FL)

Short title: Marbled crayfish impact in Madagascar

# Abstract

The negative environmental and economic impacts of many invasive species are well known. However, given the increased homogenization of ~~the~~ global biota, and the difficulty of eradicating species once established, a balanced approach to considering the impacts of invasive species is needed. The marbled crayfish (*Procambarus virginalis*) is parthenogenetic freshwater crayfish that was first observed in Madagascar around 2005 and has spread rapidly. We present the results of a socio-economic survey (n=385) in three regions of Madagascar that vary, based on ~~in~~ when the marbled crayfish ~~first~~ arrived. Respondents generally considered marbled crayfish to have a negative impact on rice agriculture and fishing, however the animals were also seen as making a positive contribution to household economy and food security. Regression modeling showed~~s~~ that respondents in regions with longer experience of marbled crayfish have more positive perceptions. Unsurprisingly, considering the perception that crayfish negatively impact rice agriculture, farmers have more negative views towards the crayfish than those involved in crayfish trading. Food preference ranking and market surveys revealed the acceptance of marbled crayfish as a cheap source of ~~cheap~~ animal protein; a clear positive in a country with widespread malnutrition. While data on ~~the~~ biodiversity impacts of the marbled crayfish invasion in Madagascar is ~~still completely~~ lacking, this study provides some insight into the socio-economic impacts of the dramatic spread of this unique invasive species~~ in Madagascar~~.

“*Biby kely tsy fantam-piaviana, mahavelona fianakaviana*” (a small animal coming from who knows where which supports the needs of the family). Fokontany worker Analamanga, Madagascar.

# Introduction

Invasive alien species can have substantial negative effects on the environment, the economy and human health [1–3]. Invasive species are major drivers of change in invaded ecosystems causing changes in habitat structure, function, and biodiversity, and can result in the extinction of species through direct predation or competition for food and space [4–6]. ~~The d~~Damage caused by invasions and the management costs for their control can be a significant economic burden [7]. Also, invasive species can play important roles in disease transmission and spread [3]. While previous research has ~~traditionally~~ focused on ~~these~~ negative impacts, the potential benefits from biological invasions can be overlooked. This may be because negative impacts are part of the definition of invasive species [8]; however, ~~but~~ there is an increasing awareness of their ~~potential~~ positive contributions [9–11]. Few studies have explored how perceptions of costs and benefits from an invasion may vary among people living with an invasive species and how this may change over time following an invasion.

Invasive crayfish have been recognized as a major threat to freshwater ecosystems. Indeed, their position in the food web (most are omnivorous) and their capacity to transmit diseases can strongly impact ecosystem functions. The rusty crayfish (*Faxionus rusticus*), for example, can significantly reduce resource availability in aquatic communities leading to biodiversity loss, while the signal crayfish (*Pacifastacus leniusculus*) transmits the crayfish plague in Europe, which has resulted in extirpation of native crayfish from many locations [12,13]. Nonetheless, invasive crayfish species can also play a positive socio-economic role. The red swamp crayfish (*Procambarus clarkii)* supports important commercial fisheries in many countries where it has been introduced [14,15], while the red claw crayfish (*Cherax*

*quadricarinatus*) is an important source of income for artisanal fishermen in Jamaica [16]. Other studies have ~~also~~ suggested that invasive crayfish in Kenya play a role in control of schistosomiasis through predation on the snail hosts of this human parasite [17].

The marbled crayfish (*Procambarus virginalis*) originated from the German aquarium trade in the early 2000s [18,19]. Marbled crayfish are descendants of the slough crayfish (*Procambarus fallax*), a sexually reproducing freshwater crayfish from Florida [20]. The ability of the marbled crayfish to reproduce without males, as well as its high fecundity and resistance to harsh environmental conditions, predisposes it to be a~~n~~ perfect invader~~sive species~~ [21,22]. Indeed, marbled crayfish have formed stable wild colonies in several European countries [23–27]. In Madagascar, marbled crayfish were first observed by biologists in markets around 2005 but were not identified until 2007 [22,28]. Initially confined to a small region around Antananarivo, their distribution area has now increased about 100-fold, covering eight out of 15 regions studied in 2017 [29]. Conservationists quickly raised concerns [19,22] about potential impacts that marbled crayfish may have on endemic freshwater biodiver~~v~~sity, including the seven species of endemic crayfish in the genus *Astacoides* [30,31]. In addition, there were also concerns that marbled crayfish may have a negative impact on rice agriculture and local freshwater fisheries. However, farmers were~~from the start,~~ ~~marbled crayfish were found in markets and farmers were~~ observed transporting marbled crayfish ~~the species~~intentionally around the island as early as 2007, ~~intentionally around the island as early as 2007,~~ suggesting that they were valuable~~considered~~ [22]. ~~valuable [22].~~ While the Ministry of Agriculture, Livestock and Fisheries initially issued legislation to prohibit the transportation of live marbled crayfish in 2009, the animals have ~~recently~~ been observed as being widely sold in markets across Madagascar, without sanctions [32].

Our study aims to update information on the distribution of marbled crayfish in Madagascar and to explore attitudes towards marbled crayfish in three regions ~~varying in~~based on how recently the marbled crayfish arrived. Specifically, we explore perceptions of the positive or negative impacts on rice agriculture, fishing and its contribution to health, food security and household income. We also seek to understand more about the markets for this

**Commented [MM1]:** Do you mean a sexually reproducing crayfish or asexually reproducing crayfish…?

species, including the relative acceptance of marbled crayfish as a source of dietary protein. While information on the ecological impacts of the invasion of marbled crayfish in Madagascar are ~~still entirely lacking,~~lacking; this paper provides valuable information on their socio-economic impacts.

# Materials and methods

## Ethics statement

Research involving human participants was approved by the Bangor University College of Environmental Sciences and Engineering research ethics committee (Approval Number: CoESE2019RAJPGJ01). Oral consent was obtained from each participant to voluntarily participate in the survey. Field research was approved by the Ministry of Environment and Sustainable Development of Antananarivo (MEDD), Madagascar (research permits No. 58/19/MEDD/SG/DGF/DSAP/SCB.Re and No. 59/19/MEDD/SG/DGF/DSAP/SCB.Re).

## Update of marbled crayfish distribution

To update information on the distribution of marbled crayfish in Madagascar, we re-visited 18 locations and 14 additional locations (see S1 Table for details) across three regions where a survey in 2017 [29] did not reveal marbled crayfish (Analanjirofo, Menabe, Vatovavy Fitovinany). We sampled the water bodies (rice fields, channels, ponds and marshes) with traditional “tandroho” nets (50cm x 30 cm x 30cm, S1 Fig.) from 8:00 to 11:00 and from 15:00 to 18:00 for three days in each location, as described previously [33]. Collections were performed by M.A. Additionally, we recorded the geographic coordinates of new locations where we observed marbled crayfish within the survey regions: Analamanga, Mahatsiatra Ambony and Ihorombe (S2 Table).

## Social survey details

The survey was carried out in March and April 2019 in three regions where marbled crayfish are present: Analamanga, Mahatsiatra Ambony and Ihorombe (see S2 Fig. and S3 Table for details). In each region, we first visited the regional authorities (Direction Regionale de l'Environnement, de l'Ecologie et des Forets, DREEF), as well as local authorities in each town or village where we worked (the commune or fokontany representative) to gain local permission for the work. The survey was conducted on weekdays and weekends.

We collected three main types of data: (1) We carried out a brief questionnairesurvey that targeted adults in the region, survey targeted at adults in the region and designed to collect quantitative information. The questionnaire contained closed-ended items and Likert response questions. We did not have a sampling frame and used convenience purposive sampling. At In each location, enumerators approached adults in markets, fields, streets and villages, or those working in / and around near water bodies. The questionnaire took approximately 15 minutes to complete. (2) For participants kwho were identified as key informants (those involved in selling crayfish, crayfish harvesting or having more knowledge on marbled crayfish), were asked some additional open-ended questions. These in-depth surveys lasted 30-45 minutes. (3) Lastly, Finally, we recorded the price of also conducted a study of 30processed marbled crayfish and other common sources of dietary protein as price per kilogram at 30 markets (containing more than 200 stalls) in Antananarivo, where we. recorded the price of processed marbled crayfish and other common sources of dietary protein as price per kilogram.

We introduced ourselves to For each respondent , we first clearly introduced ourselves and carefully explained the aim of our study and how data would be used. We provided assurance of confidentiality by explaining that we were are not taking any individually identifying information or recording voices. Due to the low level of literacy, oral consent was obtained from each respondent to voluntarily participate in the survey. Oral consent was also obtained for all photographs taken. Interviews were carried out by R.A., J.R., H.R. and M.R. with the help of trained enumerators. All interviewers were native speakers of Malagasy, comfortable in the dialects of the regions visited. Before starting an interview, each informant was shown a series of 16

**Formatted:** Indent: Hanging: 0.49", Space Before: 0.3 pt

**Formatted:** List Paragraph, Indent: Hanging: 0.49", Numbered + Level: 1 + Numbering Style: 1, 2, 3, … + Start at: 127 + Alignment: Left + Aligned at: 0.11" + Indent at: 0.6", Tab stops: 0.6", Left + 0.6", Left

**Formatted:** Indent: Hanging: 0.49", Space Before: 0.35 pt

**Formatted:** List Paragraph, Indent: Hanging: 0.49", Numbered + Level: 1 + Numbering Style: 1, 2, 3, … + Start at: 127 + Alignment: Left + Aligned at: 0.11" + Indent at: 0.6", Tab stops: 0.6", Left + 0.6", Left

**Formatted:** Indent: Hanging: 0.49", Space Before: 0.35 pt

**Formatted:** List Paragraph, Indent: Hanging: 0.49", Numbered + Level: 1 + Numbering Style: 1, 2, 3, … + Start at: 127 + Alignment: Left + Aligned at: 0.11" + Indent at: 0.6", Tab stops: 0.6", Left + 0.6", Left

photographs with crustacean and fish species to assess their knowledge of marbled crayfish

(S3 Fig.). Surveys were delivered using Open Data Kit (ODK)-Collect, an Android open source app for data collection. Data were submitted to ODK Aggregate when an internet signal was available.

We assessed the perception of marbled crayfish impacts by collecting information on the respondents' socio-economic status and their preferences. First, we showed a series ~~assessed the preference among the most common sources of protein in local diets. We showed a series~~ of 16 photographs (S4 Fig.) that illustrated~~ing~~ local sources of dietary protein ~~different animal and plant-based protein sources~~ and asked the respondents to rank them in order of preference, before recording the first five. Next, ~~We then~~ we collected information on their primary and secondary livelihood (agriculture, government position, private monthly waged work, daily waged labor, trader, wild product harvester, other). We also recorded whether people owned irrigated rice fields, and ~~whether~~if they fished commercially, for subsistence or recreation. We investigated the perception of marbled crayfish impacts on six different items (rice farming, fishing, household economy, food security, animal feed, health) using a 5-point Likert scale illustrated by Emojis (very positive to very negative, coded 1 to 5 respectively). The full survey (in English and Malagasy) is available in the Supporting Information.

## Data analysis

Data analysis was carried out using R3.4.3. Results from Likert scales were illustrated as diverging stacked bar charts using the *ggplot2* package. We ~~also~~ explored the relationships between marbled crayfish overall impact perception and potential predictors by fitting a multivariate ordinary least squares (OLS) regression. Our model investigated which factors predicted ~~whether~~ if the overall perception of marbled crayfish impact was ~~will tend to be~~ more positive or negative. The dependent variable (the overall perception of marbled crayfish impacts) was measured on a 5-point ordinal scale: 1 being very positive and 5 very negative. Eight variables were considered as potential predictors: survey location, gender, age, livelihood, relationship to marbled crayfish (harvester, trader, buyer), and the use of marbled crayfish as animal feed. All variables except age ~~are~~ were categorical and ~~were~~ transformed into

**Formatted:** Indent: Hanging: 0.49", Space Before: 0.4 pt

**Formatted:** List Paragraph, Indent: Hanging: 0.49", Numbered + Level: 1 + Numbering Style: 1, 2, 3, … + Start at: 127 + Alignment: Left + Aligned at: 0.11" + Indent at: 0.6", Tab stops: 0.6", Left + 0.6", Left

**Commented [MM2]:** Global scores are poor indicators.

dummy variables before running the regression analysis. To validate our regression model, we assessed the assumption of normality. ~~by determining whether the residuals have a normal distribution.~~ We first assessed the residuals with histogram and scatter plots. Then we performed a Shapiro-Wilk normality test on the residuals. We also checked for heteroskedasticity using the Breusch-Pagan test.

For sensitivity analysis, we also ran an ordinal regression (proportional odds logistic regression function *polr* from the R package MASS) [34,35] with the same predictors, given the level of measurement of the dependent variable, and found similar results to the OLS regression (S4 Table). We present the results of OLS regression for ease of interpretability of coefficient and model fit. Finally, we evaluated the prediction model accuracy by running a k-cross validation analysis (10-fold) followed by a repeated k-cross-validation, using the functions *cv* and *repeatedcv* from the R package CARET, with a seed set on 523 for the reproducibility of the analysis (S5 Table).

# Results

Compared to a previous survey ~~from~~ in 2017 [29] we did not record an expansion of the known range of the marbled crayfish in Madagascar during this survey (March and April 2019). Re-sampling at 18 previously negative sites, and 14 additional locations distributed in the Analanjirofo, Menabe and Vatovavy Fitovinany regions revealed no marbled crayfish (Fig. 1A, S1 Table). However, marbled crayfish were discovered at 15 additional sites in the Analamanga, Mahatsiatra Ambony and Ihorombe regions (i.e., within the previously identified range) during the present survey (Fig. 1A, S2 Table). The increasing number of populations discovered in these regions, either in private ponds, in rice fields or in public freshwater ecosystems, likely reflects the continuing anthropogenic distribution due to commercial exploitation [32].

**Commented [MM3]:** Best suited for discussion, not results.

We conducted ~~carried out~~ social surveys in three regions (Fig. 1A, S3 Table): Analamanga (mostly in Antananarivo at 12 sampling locations), Mahatsiatra Ambony (mostly in Fianarantsoa at 8 sampling locations) and Ihorombe (mostly in Ihosy at 3 sampling locations). Using ~~We used convenience~~ purposive sampling, we ~~targeting the adult population, and~~ approached a total of 435 adults ~~435 persons in total~~ (Fig. 1B) and ~~. We~~ obtained good participation rates (>86%) in all three regions (Fig.1B). The sample consisted of ~~, with~~ 142, 143 and 100 participants in Analamanga, Mahatsiatra Ambony and Ihorombe, respectively. The average participant age was 39 years, with 54% male and 46% female participants. Farming was t~~T~~he most commonly reported ~~primary~~ job ~~livelihood~~ among respondents ~~was farming~~ (48%).

**Commented [MM4]:** This is a value judgment. Please allow the read to make this observation if they choose to do so.

**Fig. 1. Distribution of marbled crayfish in Madagascar.** (A) Map of Madagascar. Light green dots indicate sites with known marbled crayfish populations from 2017 [29]. Dark green dots indicate additional sites where marbled crayfish were detected during this study (in 2019), light blue dots indicate 3 re-sampled negative regions from 2017 with no marbled crayfish. The three regions where the social surveys presented in this paper were carried out are highlighted by color. (B) Participant response rates and numbers of survey respondents per region. (C) Earliest observation of marbled crayfish in the three regions. Numbers indicate % respondents.

A subset of respondents (n=275) were asked when they first observed marbled crayfish in their ~~local~~ area. In the Analamanga region, a substantial fraction of respondents indicated that they became first aware of marbled crayfish before 2005 and the animals became well-known by 2010 (Fig. 1C). In contrast, marbled crayfish were mostly unknown in Mahatsiatra Ambony and completely unknown in Ihorombe before 2011 (Fig. 1C). This is consistent with a spread of the animals from the center to the south of Madagascar.

To explore the perception of marbled crayfish impacts, we used a Likert scale to investigate six variables. ~~The r~~Results showed that attitudes towards marbled crayfish varied~~y~~ substantially by region. In general, ~~with~~ respondents in the two southern regions (Mahasiatra Ambony

and Ihorombe) had~~ving generally~~ more negative attitudes towards the crayfish, while those in Antananarivo were ~~generally~~ more positive (Fig. 2). However, in all three regions, respondents reported both positive and negative impacts of marbled crayfish. Negative impacts on rice farming and fishing were reported at all sites (and by nearly 100% of respondents in Ihorombe), while contribution to food security and household economy, in general, ~~were~~ were ~~generally~~ seen as positive.

**Fig. 2. Perceived socio-economic impacts of marbled crayfish in 3 regions of Madagascar.** Bar plots show overall impacts (Likert scales) in the three regions.

~~The r~~Responses to the open-ended questions by ~~the~~ key informants (52 out of 351 participants) provided some useful ~~qualitative~~ information to support and explain the quantitative data. The reasons for widely reported negative impacts of marbled crayfish on rice fields was due to burrowing activities, which dry up the rice fields and require the farmers to regularly repair their banks and irrigation canals. For example: "the marbled crayfish destroy the mud walls we build around our rice fields" (S6 Table, Q1-3). This can also lead to arguments between rice field owners as they would accuse each other to have dried the rice field by digging burrows.

The occasional positive comments about the impacts of marbled crayfish on rice farming ca~~o~~me from the perception that marbled crayfish burrowing activities could~~an~~ improve rice production by loosening and aerating the soil (S6 Table, Q4). ~~The~~ P~~p~~erceived impacts of marbled crayfish on fishing were overwhelmingly negative~~,~~ because ~~the~~ crayfish are thought ~~belived~~ to predate on young fish (though not larger fish; S6 Table, Q5). Importantly, some impacts may not be directly observed after an initial marbled crayfish colonization: "the impacts on fishing can~~-~~not really be seen since the marbled crayfish are present for only two years" (S6 Table, Q6). Many people mentioned positive impacts from crayfish harvesting on local livelihoods from catching and selling crayfish, or using them as a cheap source of household protein or animal feed. A Fokontany worker in a village with several households who depend~~ings~~ on marbled crayfish described ~~marbled crayfish~~ it as “a small

**Formatted:** Indent: Left: 0.11", Hanging: 0.49", No bullets or numbering, Tab stops: Not at 0.6" + 0.6"

animal coming from who knows where which supports the needs of the family" (S6 Table, Q7). Similarly, a farmer reported that marbled crayfish had given him a good living for about 10 years ~~since~~ after they first arrived in the area (S6 Table, Q8). Marbled crayfish were often mentioned as a good and cheap replacement for meat, and a source of energy and strength (S6 Table, Q9). However, some respondents ~~have also observed~~ experienced allergic reactions, and stomach pain due to consumption of marbled crayfish (S6 Table, Q10).

**Commented [MM5]:** Marbled crayfish is also meat, don't you mean cheap replacement for protein?

In order to link ~~the~~ overall perception of marbled crayfish impacts to socio-economic characteristics of respondents, we selected eight variables as predictors in a multivariate linear model. Our linear regression model (OLS) explored~~s~~ the relationship between these variables, and overall perceptions towards marbled crayfish (Fig. 3, Table 1) show a good overall fit: the model successfully explains 44% of the variation of the dependent variable (adjusted R squared of 0.438). The residuals satisfy the assumption of normality (Shapiro-Wilk normality test on the residuals: p=0.603), confirmed visually by a Q-Q plot (S5C Fig.). There is no observed heteroskedasticity in the data (Breusch-Pagan test chi square statistic = 0.020, p = 0.886). Additionally, 10-fold cross-validation of our regression analysis revealed a good performance with an R-squared of 0.42, ~~thus~~ providing additional confidence in the results of the regression analysis.

A positive overall attitude towards the marbled crayfish is associated with study site, as participants in Analamanga showed a significant positive association (+0.95 scale points) and Ihosy a significant negative association (-0.44 scale points) compared to Fianarantsoa (controlling for gender, age and livelihood). Those involved in crayfish harvesting ~~are~~ were more positive towards marbled crayfish than non-harvesters (+0.67 scale points). A ~~lso, the same~~similar scenario ~~picture~~ was observed for crayfish customers and those who us~~ing~~ed marbled crayfish as animal feed ~~with~~ (respectively 0.46 and 0.27 scale points more positive compared to those who do not buy and use marbled crayfish). Finally, livelihood was~~is~~ significantly associated with ~~the~~ overall perception, with traders (and our grouping of 'other' livelihoods) having a more positive average scores compared to farmers. This is ~~perhaps un~~finding is not surprising, ~~as~~ since farmers ~~will~~often suffer negative impacts incurred ~~associated with the damage exerted~~ by marbled crayfish on rice fields, while traders tend ~~are more likely~~ to profit from selling them~~crayfish~~.

**Fig. 3. Coefficient plot of the model exploring the relationship between overall perception of marbled crayfish and 8 socio-economic predictors.** Blue bars represent the coefficients for locations while yellow bars show the coefficients for livelihoods, black bars illustrate the coefficients for crayfish-related activities and green bars the coefficient for demographic controls. Error bars represent 95% confidence intervals. Gender: male, and age were included as controls. The intercept was defined by the following parameters: location: Mahatsiatra Ambony; livelihood: farmer, not harvesting crayfish, not selling crayfish, not buying crayfish, not using crayfish as animal feed. P-values of each predictor are provided in Table 1.

**Table 1. Regression table. The dependent variable is defined by the overall perception of marbled crayfish.**

| **Predictors** | | **Estimate** | **Std. Error** | **t-value** | **p-value** |
|---|---|---|---|---|---|
| Location | Antananarivo | 0.952 | 0.147 | 6.465 | 0.001*** |
| | Ihosy | -0.439 | 0.138 | -3.170 | 0.002 ** |
| Livelihood | Trader | 0.347 | 0.162 | 2.145 | 0.033* |
| | Wild product harvester | -0.051 | 0.214 | -0.237 | 0.813 |
| | Other | 0.335 | 0.157 | 2.135 | 0.033* |
| Crayfish harvester | Yes | 0.674 | 0.162 | 4.162 | 0.001*** |
| Crayfish vendor | Yes | 0.156 | 0.162 | 0.959 | 0.338 |
| Crayfish customer | Yes | 0.466 | 0.134 | 3.483 | 0.001*** |
| Crayfish as animal feed | Yes | 0.270 | 0.119 | 2.262 | 0.024* |
| Gender | Male | 0.078 | 0.112 | 0.693 | 0.489 |
| Age | Continuous | -0.007 | 0.004 | -1.672 | 0.095 |
| Intercept[1] | | -0.846 | 0.199 | -4.242 | 0.001*** |
| Observations | | 351 | | | |
| $R^2$ | | 0.456 | | | |
| **Adjusted $R^2$** | | **0.438** | | | |
| Residual Std. Error | | 0.971 (df=339) | | | |
| F Statistic | | 25.778 (df=11; 339) | | | |

Note: *significant at <0.05, **significant at <0.01, ***significant at <0.001
[1]Location: Fianarantsoa, Livelihood: Farmer, Not harvesting crayfish, Not selling crayfish, Not buying crayfish, Not using crayfish as animal feed and Gender: Female.

To assess the popularity of marbled crayfish in the diet of our ~~survey~~ participants, we asked them to identify and rank their five most preferred sources of nutritional protein~~s~~ among those illustrated in 16 photographs ~~of dietary protein sources provided~~ (S4 Fig.). ~~The r~~Results showed that tilapia and traditionally reared chicken were~~are~~ the most preferred dietary protein (Fig. 4), which is consistent with previous findings [36,37]. Marbled crayfish meat was less preferred and grouped together with shrimp~~s~~, factory-produced chicken meat and beef liver (Fig. 4). Several other dietary protein sources, such as tripe, beans and crabs were less preferred than marbled crayfish.

**Fig. 4. Preferences and relative prices per kilogram for a selection of common sources of dietary protein.** Photographs and descriptions are provided in S4 Fig. Left panel: results of preference ranking for sources of dietary protein. Right panel: mean of market prices per kilogram for each dietary protein source (n=number of stalls where prices were recorded in the 30 Antananarivo markets visited). Error bars represent 95% confidence intervals. For comparison, the price of live marbled crayfish from vendors in Ihorombe (1,200 MGA/kg, approximately 0.3 EUR) is also indicated.

In Antananarivo (Analamanga region), marbled crayfish were mainly sold in markets (Fig. 5A) and their leftover carapaces were used as animal feed (Fig. 5B). We found marbled crayfish in 33 stalls in 11 markets (out of 30 markets investigated). They were mostly ~~ainly~~ sold as boiled tail meat, with an average price per kg of 8,600 MGA (approximately 2 EUR, also see Fig. 4). This strongly contrasts with Ihosy (Ihorombe region), where marbled crayfish were ~~predominantly~~ sold live in large bags for widespread distribution (Fig. 5C). The marbled crayfish business is structured differently in Ihosy compared to Antananarivo: orders are received from different locations and regions. Traders then purchase live marbled crayfish from local collectors in response to ~~the~~ market demand. Up to 20 bags of 35 to 80 kg with live marbled crayfish are transported daily with short and long-distance "taxi-brousse" for a

fixed fee per bag during the rainy season (November to March). The price of live marbled crayfish is much lower with an average of 1,200 MGA/kg (approximately 0.3 EUR).

**Fig. 5. Commercial sales and distribution of marbled crayfish.** (A) Marbled crayfish meat sold with freshwater fish. (B) Leftover shells from marbled crayfish used as animal feed. (C) Bag of live marbled crayfish for distribution. (D) Live and boiled marbled crayfish sold on the street. (E) Boiled and salted marbled crayfish sold in the market. (F) Ready-to-eat marbled crayfish sold in street food markets.

Interestingly, Fianarantsoa (Mahatsiatra Ambony region) was mentioned as one of the cities where people bought~~uy~~ marbled crayfish from Ihosy. This was confirmed by interviews of marbled crayfish vendors in Fianarantsoa's evening market who indicated that they bought~~buy~~ marbled crayfish from Ihosy, and usually sold~~ell~~ their entire ~~complete purchase~~ amount within a few hours (Fig. 5D).~~ In Fianarantsoa ,~~ Mostly, marbled crayfish were ~~usually~~ sold as processed food in Fianarantsoa (Fig. 5E). Marbled crayfish beignets and meals with marbled crayfish in tomato and green onion sauce (Fig. 5F) were offered in several street food locations and street market restaurants. D~~Interestingly, d~~espite an official prohibition from the Ministry of Agriculture, Livestock and Fisheries (effective since 2009) that forbids the transport of live marbled crayfish, the transport of marbled crayfish (for example by taxi-brousse), is supported by regular permits and fees. We interviewed taxi-brousse drivers from different transport companies in Ihorombe, who stated that those who transport marbled crayfish pay for a yearly permit, similar to transport permits for other freshwater products.

# Discussion

~~It is now indisputable that we live in t~~The Anthropocene is ~~; ~~a proposed geologicalal epoch ~~age~~ defined by humanity's impact on the environment [38]. ~~In t~~This situation~~,~~ has caused some ecologists to~~are rethinking~~ rethink their position ~~response to~~ on invasive species~~ and increasingly~~, one that ~~recognize~~recognizes ~~that~~ non-natives~~ species~~ as~~re~~ a ~~part of~~part ~~the~~of future ~~ecology~~ecology ~~of the future~~ [39,40]. Although ~~While~~ the ~~strongly~~ negative impacts of ~~some~~ invasive species ~~render this~~ are controversial ~~view~~ [41], some of those involved in managementing ~~invasive species~~ have pointed out that their benefits ~~from such species~~ are often overlooked or under-reported [40]. Our results, which provide the first information on the socio-economic impacts of a parthenogenetic decapod described as 'the perfect invader' [22], provide an interesting ~~useful~~ case study of ~~in~~ how people make use of a new species in their environment. It also provides information useful for predicting the likely ~~future~~ spread of marbled ~~this invasive~~ crayfish in a country having ~~with~~ freshwater biodiversity of global value [42].

Madagascar is one of the poorest countries in the world, with 75% of the population living on less than $1.90 per day [43,44]. Poverty manifests itself in limited intake of dietary protein leading to stunting [45] which affects 1 in 2 Malagasy children [46]. In fact, many people rarely consume animal protein [36,37]. Given the lack of sufficient protein for human consumption; livestock are mostly fed rice bran, roots and tubers [47], providing insufficient protein for production. New and cheap sources of animal protein ~~therefore have the potential~~can create ~~to have~~ a significant ~~positive~~ impact on humans. Positive perceptions of marbled crayfish (especially prevalent in the Analamanga region) are ~~strongly~~ associated with harvesting, buying or using marbled crayfish for animal feed~~s~~. The fact that the crayfish can be ~~quite easily~~ collected easily from public lakes, rivers, canals and rice fields also means that ~~they~~ it can be done ~~collected~~ cheaply~~at very low cost~~. Live animals are sold at low prices (live marbled crayfish rank among the cheapest sources of animal protein available), which makes them affordable for poorer people. Leftover parts

**Commented [MM6]:** What does this mean?

from processed marbled crayfish are fed to animals (mainly chicken and pigs); providing an additional source of income for those involved in the crayfish trade and providing an effective, cheap and protein-rich livestock feed.

In Antananarivo, where the species has been common for at least 10 years [22], the market has evolved and mostly peeled tail meat is sold. This value-added product (similar to

**Commented [MM7]:** Needs re-wording

processed fish and pre-cut vegetables) increases the profit margin of ~~for the~~ crayfish. A different market exists in Ihorombe, where the animals appeared considerably later and most trade is for live crayfish, in bulk. While marbled crayfish are seldom among respondent's most preferred food, they are ranked similarly to shrimp~~s~~ and factory processed chicken. The low price, the ease of availability, and the development of new culinary ~~recipies~~recipes for marbled crayfish could be drivers of their increasing acceptance.

The most significant negative impacts reported were on rice farming and fishing. A related species of crayfish (*Procambarus clarkii*) is known to negatively affect irrigated rice production in the Iberian Peninsula, where it damages banks and irrigation canals by burrowing [48,49]. However, the extent to which rice production is impacted by marbled crayfish in Madagascar remains poorly understood and our survey results, associated qualitative information, provided some ~~what~~ contradictory evidence. In Analamanga (where the crayfish have been present over the longest period of time), the largest proportion of respondents reported no impact, while some reported a positive impact, perhaps because of the impact of the crayfish on soil aeration. It is possible that reported negative impacts of crayfish stem from ~~the~~ extensive media reporting of possible negative impacts on rice, rather than firsthand experience. Indeed, national daily newspapers such as Midi Madagasikara, L'Express de Madagascar or local newspapers, such as Tia Tanindrazana started to describe the marbled crayfish as a threat to rice farming and as a national threat from 2007 [50–53]. The available evidence suggests negative impacts on fishing, but further research is ~~urgently~~ needed to ~~better~~ understand the impacts on fish and freshwater ecosystems.

The overall perception of marbled crayfish impacts ~~was significantly~~ differed among ~~nt in~~ participants in the three regions that were surveyed. ~~Participants~~ Individuals in ~~the~~ Analamanga ~~region~~ perceived crayfish more favorably than ~~participants~~ those in the Mahatsiatra Ambony and Ihorombe regions. This may be due to differences in livelihoods of our sample ~~population~~ in the three regions. Those interviewed in the Mahatsiatra Ambony and Ihorombe regions rel~~y~~ied more on farming, particularly rice farming. ~~, and p~~Perceived negative impacts on rice farming are likely to ~~strongly~~ influence the overall negative perception. Another possible explanation is that people in

**Commented [MM8]:** There's no such thing as a sample population.

Analamanga (where marbled crayfish have been established the longest; since about 2005) people have had greater more exposure time to get used to the species, thus more time ' presence and to adapt to the opportunities it presents..

Acceptability The finding that of the marbled crayfish, are widely accepted, especially around Anranarivo, is noticeable given the negative cultural associations which have developed since it the crayfish first appearedoccured. The local name for the crayfish which was coined in Malagasy for the crayfish ("foza orana"; literally, crab-like crayfish) quickly took on the meaning of something which is poor in quality and very abundant. For example, tThe meaning of the term has evolved over the years and is been also used toapplied to many things such as describe low-quality cell phones, and low-class prostitutes [54].

The geographical range of many invasive species is driven by socio-economic factors, such as trade, travel and the movement of goods [55,56]. Marbled crayfish were already sold in markets in Madagascar soon after they first appeared. This raised the possibility that their potential economic importance could promote their spread invasiveness [22]. Our work clearly confirms that the market demand for the crayfish is likely to encourage spread into new areas. Even in Ihorombe, where the overall perception of the crayfish is negative, the strong market demand and widespread distribution of live animals are likely to favor further spread.

LastlyFinally, it is also important to point out some the limitations of our analysis. The sampling approach used, and the relatively small numbers of interviews, means that our sample is not fully representative of the population in each region. However, Given the resources and time available, probabilistic sampling was not possible, given our limited resources. However, we Our sampled included several points in each region (see maps in S2 Fig.) and the high response rate means that the responses are not likely to be biased. Furthermore, we controlled for differences in age and gender.

**Formatted:** Indent: Hanging: 0.49", Space Before: 0.4 pt

**Formatted:** List Paragraph, Indent: Hanging: 0.49", Numbered + Level: 1 + Numbering Style: 1, 2, 3, … + Start at: 339 + Alignment: Left + Aligned at: 0.11" + Indent at: 0.6", Tab stops: 0.6", Left + 0.6", Left

# Conclusion

The spread of the marbled crayfish in Madagascar over the last decade has been nothing short of dramatic. Given the practical challenges to ~~the~~ eradicate this ~~ion of~~ invasive species, a balanced approach for ~~to~~ considering the costs of benefits arising from invasiveness is needed [40]. There are differing ~~overall~~ perceptions towards marbled crayfish; ~~both~~ between regions and between people with different socio-economic characteristics. However, it is clear that the market for marbled crayfish, and their widespread consumption, are likely to result in further spread. It is difficult to imagine any inhabitable parts of Madagascar that will remain uninvaded over the next decade. What this will mean for Madagascar’s freshwater biodiversity is ~~still completely~~ unknown. This study has attempted to provide some insight into what impact the invasion has on local people’s livelihoods. An important area of uncertainty remains the impact of marbled crayfish on rice agriculture. Given the importance of irrigated rice production to the economy of Madagascar, this is ~~certainly~~certainly, worthy of further investigation. Our study ~~clearly~~ demonstrated~~s~~ that marbled crayfish have ~~rapidly~~ become a widely consumed and ~~,~~ cheap source of animal protein in local diets. Given the desperate need for protein in the diets of many Malagasy people, this can be considered a positive development.

# Acknowledgements

We thank Maevatiana N. Ratsimbazafindranahaka and Luticia V. Raharimalala for their valuable support of our survey, and Amy Lewis for help with coding the survey in Open Data Kit. We also thank Soatiana Daniel and Laza S. Andriantsoa for their help with fieldtrip management.

# References


1. McNeely, J.A., H.A. Mooney, L.E. Neville, P. Schei and JKW. A Global Strategy on Invasive Alien Species. 2001.
2. Pyšek P, Richardson DM. Invasive Species, Environmental Change and Management, and Health. Annu Rev Environ Resour. 2010;35: 25–55. doi:10.1146/annurev-environ-033009-095548
3. Giuseppe M, Elena T. Invasive species and human health. Ethol Ecol Evol. 2018. doi:10.1079/9781786390981.0000
4. Hejda M, Pyšek P, Jarošík V. Impact of invasive plants on the species richness, diversity and composition of invaded communities. J Ecol. 2009;97: 393–403. doi:10.1111/j.1365-2745.2009.01480.x
5. Seeney A, Eastwood S, Pattison Z, Willby NJ, Bull CD. All change at the water's edge: invasion by non-native riparian plants negatively impacts terrestrial invertebrates. Biol Invasions. 2019;21: 1933–1946. doi:10.1007/s10530-019-01947-5
6. Clavero M, García-Berthou E. Invasive species are a leading cause of animal extinctions. Trends Ecol Evol. 2005;20: 110. doi:10.1016/j.tree.2005.01.003
7. Hoffmann BD, Broadhurst LM. The economic cost of managing invasive species in Australia. NeoBiota. 2016;31: 1–18. doi:10.3897/neobiota.31.6960
8. Bellard C, Genovesi P, Jeschke JM. Global patterns in threats to vertebrates by biological invasions. Proc R Soc B Biol Sci. 2016;283. doi:10.1098/rspb.2015.2454
9. Rogers P, Nunan F, Fentie AA. Reimagining invasions: The social and cultural impacts of Prosopis on pastoralists in southern Afar, Ethiopia. Pastoralism. 2017;7: 1–13. doi:10.1186/s13570-017-0094-0
10. Ngorima A, Shackleton CM. Livelihood benefits and costs from an invasive alien tree (Acacia dealbata) to rural communities in the Eastern Cape, South Africa. J Environ Manage. 2019;229: 158–165. doi:10.1016/j.jenvman.2018.05.077
11. Shackleton RT, Shackleton CM, Kull CA. The role of invasive alien species in shaping

local livelihoods and human well-being: A review. J Environ Manage. 2019;229: 145–157. doi:10.1016/j.jenvman.2018.05.007

12. Bobeldyk AM, Lamberti GA. A Decade after Invasion: Evaluating the Continuing Effects of Rusty Crayfish on a Michigan River. J Great Lakes Res. 2008;34: 265–275. doi:10.3394/0380-1330(2008)34[265:ADAIET]2.0.CO;2

13. Jernelöv A. Crayfish Plague in Europe. 2017. pp. 91–103. doi:10.1007/978-3-319-55396-2_7

14. FAO. Cultured aquatic fact sheets: Procambarus clarkii. 2009 [cited 17 Oct 2019]. Available: www.fao.org/fishery/culturedspecies/Procambarus_clarkii/en

15. Wang Q, Ding H, Tao Z, Ma D. Crayfish (Procambarus clarkii) Cultivation in China: A Decade of Unprecedented Development. Aquaculture in China: Success Stories and Modern Trends. 2018. pp. 363–377. doi:10.1002/9781119120759.ch4_1

16. Pienkowski T, Williams S, McLaren K, Wilson B, Hockley N. Alien invasions and livelihoods: Economic benefits of invasive Australian Red Claw crayfish in Jamaica. Ecol Econ. 2015;112: 68–77. doi:10.1016/J.ECOLECON.2015.02.012

17. Mkoji GM, Hofkin B V., Kuris AM, Stewart-Oaten A, Mungai BN, Kihara JH, et al. Impact of the crayfish Procambarus clarkii on Schistosoma haematobium transmission in Kenya. Am J Trop Med Hyg. 1999;61: 751–759. doi:10.4269/ajtmh.1999.61.751

18. Lyko F. The marbled crayfish (Decapoda: Cambaridae) represents an independent new species. Zootaxa. 2017;4363: 544–552. doi:10.11646/zootaxa.4363.4.6

19. Scholtz G, Braband A, Tolley L, Reimann A, Mittmann B, Lukhaup C, et al. Parthenogenesis in an outsider crayfish. Nature. 2003;421: 806. doi:10.1038/421806a

20. Martin P, Dorn NJ, Kawai T, van der Heiden C, Scholtz G. The enigmatic Marmorkrebs (marbled crayfish) is the parthenogenetic form of Procambarus fallax (Hagen, 1870). Contrib to Zool. 2010;79: 107–118. doi:10.1163/18759866-07903003

21. Feria TP, Faulkes Z. Forecasting the distribution of Marmorkrebs, a parthenogenetic crayfish with high invasive potential, in Madagascar, Europe, and North America.

Aquat Invasions. 2011;6: 55–67. doi:10.3391/ai.2011.6.1.07

22. Jones JPG, Rasamy JR, Harvey A, Toon A, Oidtmann B, Randrianarison MH, et al. The perfect invader: A parthenogenic crayfish poses a new threat to Madagascar's freshwater biodiversity. Biol Invasions. 2009;11: 1475–1482. doi:10.1007/s10530-008-9334-y

23. Lokkös A, Müller T, Kovács K, Várkonyi L, Specziár A, Martin P. The alien, parthenogenetic marbled crayfish (Decapoda: Cambaridae) is entering Kis-Balaton (Hungary), one of Europe's most important wetland biotopes. Knowl Manag Aquat Ecosyst. 2016;6. doi:10.1051/kmae/2016003

24. Patoka J, Buřič M, Kolář V, Bláha M, Petrtýl M, Franta P, et al. Predictions of marbled crayfish establishment in conurbations fulfilled: Evidences from the Czech Republic. Biologia (Bratisl). 2016;71: 1380–1385. doi:10.1515/biolog-2016-0164

25. Pârvulescu L, Togor A, Lele SF, Scheu S, Șinca D, Panteleit J. First established population of marbled crayfish Procambarus fallax (Hagen, 1870) f. virginalis (Decapoda, Cambaridae) in Romania. BioInvasions Rec. 2017;6: 357–362. doi:10.3391/bir.2017.6.4.09

26. Deidun A, Sciberras A, Formosa J, Zava B, Insacco G, Corsini-Foka M, et al. Invasion by non-indigenous freshwater decapods of Malta and Sicily, central Mediterranean Sea. J Crustac Biol. 2018;076: 1–6. doi:10.1093/jcbiol/ruy076

27. Lipták B, Mrugała A, Pekárik L, Mutkovič A, Grul'a D, Petrusek A, et al. Expansion of the marbled crayfish in Slovakia: Beginning of an invasion in the Danube catchment? J Limnol. 2016;75: 305–312. doi:10.4081/jlimnol.2016.1313

28. Kawai T, Scholtz G, Morioka S, Ramanamandimby F, Lukhaup C, Hanamura Y. Parthenogenetic Alien Crayfish (Decapoda: Cambaridae) Spreading in Madagascar. J Crustac Biol. 2009;29: 562–567. doi:10.1651/08-3125.1

29. Gutekunst J, Andriantsoa R, Falckenhayn C, Hanna K, Stein W, Rasamy J, et al. Clonal genome evolution and rapid invasive spread of the marbled crayfish. Nat Ecol Evol. 2018;2: 567–573. doi:10.1038/s41559-018-0467-9

30. Jones JPG, Andriahajaina FB, Hockley NJ, Crandall KA, Ravohangimalala OR. The ecology and conservation status of Madagascar's endemic freshwater crayfish (Parastacidae; Astacoides). Freshw Biol. 2007;52: 1820–1833. doi:10.1111/j.1365-2427.2007.01766.x

31. Boyko CB, Ravoahangimalala OR, Randriamasimanana D, Razafindrazaka TH. Astacoides hobbsi, a new crayfish (Crustacea: Decapoda: Parastacidae) from Madagascar. Zootaxa. 2005; 41–51. doi:10.11646/zootaxa.1091.1.3

32. Andriantsoa R, Tönges S, Panteleit J, Theissinger K, Carneiro VC, Rasamy J, et al. Ecological plasticity and commercial impact of invasive marbled crayfish populations in Madagascar. BMC Ecol. 2019;19: 1–10. doi:10.1186/s12898-019-0224-1

33. Andriantsoa R, Tönges S, Panteleit J, Theissinger K, Carneiro VC, Rasamy J, et al. Ecological plasticity and commercial impact of invasive marbled crayfish populations in Madagascar. BMC Ecol. 2019;19: 8. doi:10.1186/s12898-019-0224-1

34. Agresti A. Logistic Regression. Second Ed. Categorical Data analysis. Second Ed. John Wiley & Sons, Inc.; 2002. doi:10.1002/0471249688.ch5

35. Venables W, Ripley B. Modern Applied Statistics with S. Fourth Ed. Srpinger-Verlag New York; 2002. doi:10.1007/978-0-387-21706-2

36. Jenkins RKB, Keane A, Rakotoarivelo AR, Rakotomboavonjy V, Randrianandrianina FH, Razafimanahaka HJ, et al. Analysis of patterns of bushmeat consumption reveals extensive exploitation of protected species in eastern madagascar. PLoS One. 2011;6. doi:10.1371/journal.pone.0027570

37. Kurien J, López Ríos J. Flavouring Fish into Food Security. Smart Fish Publ. 2013; 173. Available: http://tonypiccolo.wix.com/smartfish2#!about1/c15k8%5Cnhttp://www.commissionoceanindien.org/accueil/%5Cnhttp://www.fao.org/documents/card/en/c/4fda7ab4-eab5-4d9d-87f9-247625d0a424/

38. Lewis SL, Maslin MA. Defining the Anthropocene. Nature. 2015;519: 171. Available: https://doi.org/10.1038/nature14258

39. Carroll SP. Conciliation biology: The eco-evolutionary management of permanently invaded biotic systems. Evol Appl. 2011;4: 184–199. doi:10.1111/j.1752-4571.2010.00180.x
40. Bonanno G. Alien species: to remove or not to remove? That is the question. Environ Sci Policy. 2016;59: 67–73. doi:https://doi.org/10.1016/j.envsci.2016.02.011
41. Russell JC, Blackburn TM. The Rise of Invasive Species Denialism. Trends Ecol Evol. 2017;32: 3–6. doi:https://doi.org/10.1016/j.tree.2016.10.012
42. Benstead JP, Rham PH De, Gattolliat J, Loiselle P V, Sartori M, Sparks JS, et al. Conserving Madagascar ' s Freshwater Biodiversity. Sci York. 2009;53: 1101–1111.
43. World Bank; Macro Povery Outlook. Madagascar. 2016.
44. World Bank. Madagascar Country Overview. 2019.
45. Semba RD. The rise and fall of protein malnutrition in global health. Ann Nutr Metab. 2016;69: 79–88. doi:10.1159/000449175
46. International Food Policy Research Centre. Global Hunger Index: Madagascar. 2018.
47. FAO. Madagascar Livestock sector brief. Fao. 2005; 20. doi:10.1111/j.1728-4465.2005.00065.x
48. Anastácio PM, Marques JC. Crayfish, Procambarus clarkii, effects on initial stages of rice growth in the lower Mondego River valley (Portugal). Freshw Crayfish. 1997;11: 608–617.
49. Anastácio PM, Soares M, Correia AM. Crayfish ( Procambarus clarkii ) consumption of wet-seeded rice plants ( Oryza sativa ): modifications throughout the rice growing period . SIL Proceedings, 1922-2010. 2005;29: 849–851. doi:10.1080/03680770.2005.11902800
50. V A. Madagascar: Ecrevisse mabrée - Danger pour la riziculture et la faune aquatique. Midi Madagasikara. Nov 2007.
51. R N. Madagascar: “Foza orana” - Cette espèce est un danger national. Midi Madagasikara. Aug 2010.
52. Andriamanoro T. Bemiray - Histoire - Le rouge et la rose: Predateurs - Haro sur les

"foza orana." L'Express de Madagascar. Jul 2018.

53. Patti. Zava-misy: Mirongatra ny foza orana. Tia Tanindrazana. Mar 2012.

54. Stoebenau K, Nixon SA, Rubincam C, Willan S, Zembe YZN, Tsikoane T, et al. More than just talk: The framing of transactional sex and its implications for vulnerability to HIV in Lesotho, Madagascar and South Africa. Global Health. 2011;7: 34. doi:10.1186/1744-8603-7-34

55. Tabak MA, Piaggio AJ, Miller RS, Sweitzer RA, Ernest HB. Anthropogenic factors predict movement of an invasive species. Ecosphere. 2017;8. doi:10.1002/ecs2.1844

56. Hulme PE. Invasion pathways at a crossroad: Policy and research challenges for managing alien species introductions. J Appl Ecol. 2015;52: 1418–1424. doi:10.1111/1365-2664.12470

# Supporting information captions

**S1 Fig. Marbled crayfish collection method with the traditional fishing tool "tandroho".**

**S2 Fig. Map of survey locations.**

**S3 Fig. Pictures of crustaceans and fish for marbled crayfish identification.**

**S4 Fig. Pictures representing sources of dietary proteins.**

**S5 Fig. Histograms and scatter plot of data and residuals.**

**S1 Table. 2019 status of the marbled crayfish presence and absence in the three regions sampled and with no marbled crayfish discovery in 2017.**

**S2 Table. Updated discovery stations of marbled crayfish in discovery regions previously described in 2017.**

**S3 Table. Geographic coordinates of survey locations.**

**S4 Table. Comparative analysis of OLS and ordinal regression.**

**S5 Table. K-cross-validation analysis.**

**S6 Table. Quotes in English and in Malagasy.**

Figure 1

Click here to access/download;Figure;Figure 1.tif

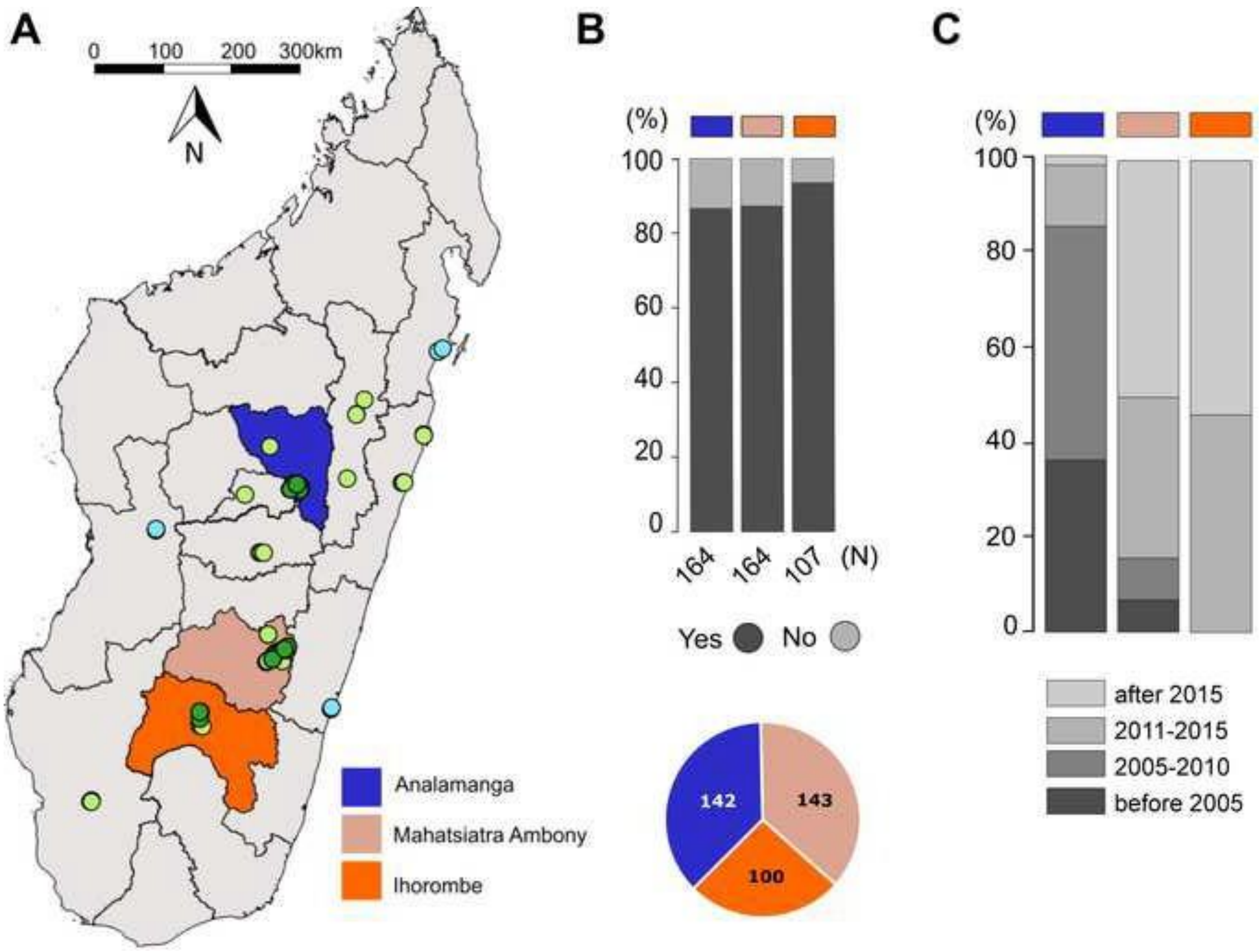

Figure 2

Click here to access/download;Figure;Figure 2.tif

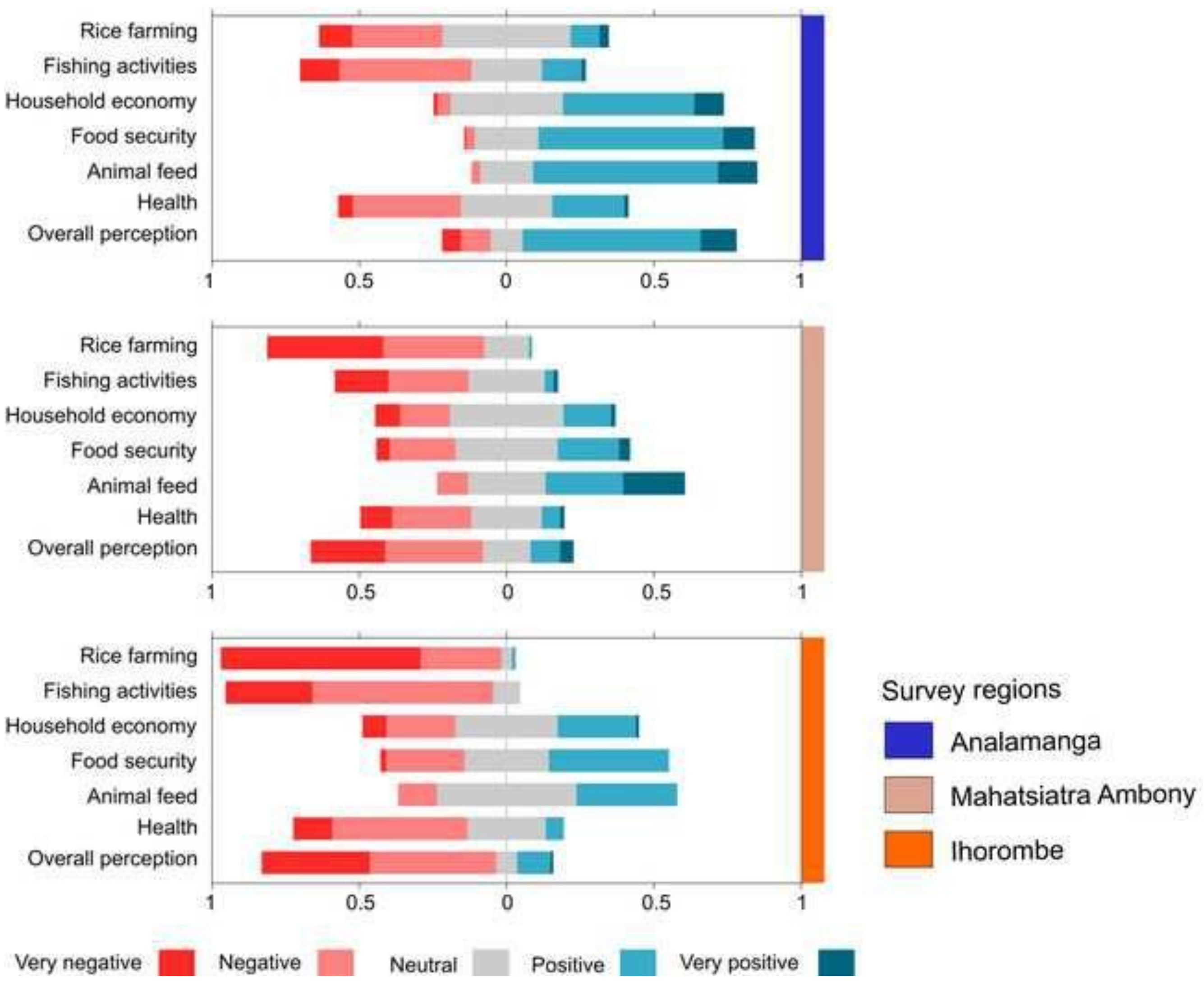

Figure 3

Click here to access/download;Figure;Figure 3.tif

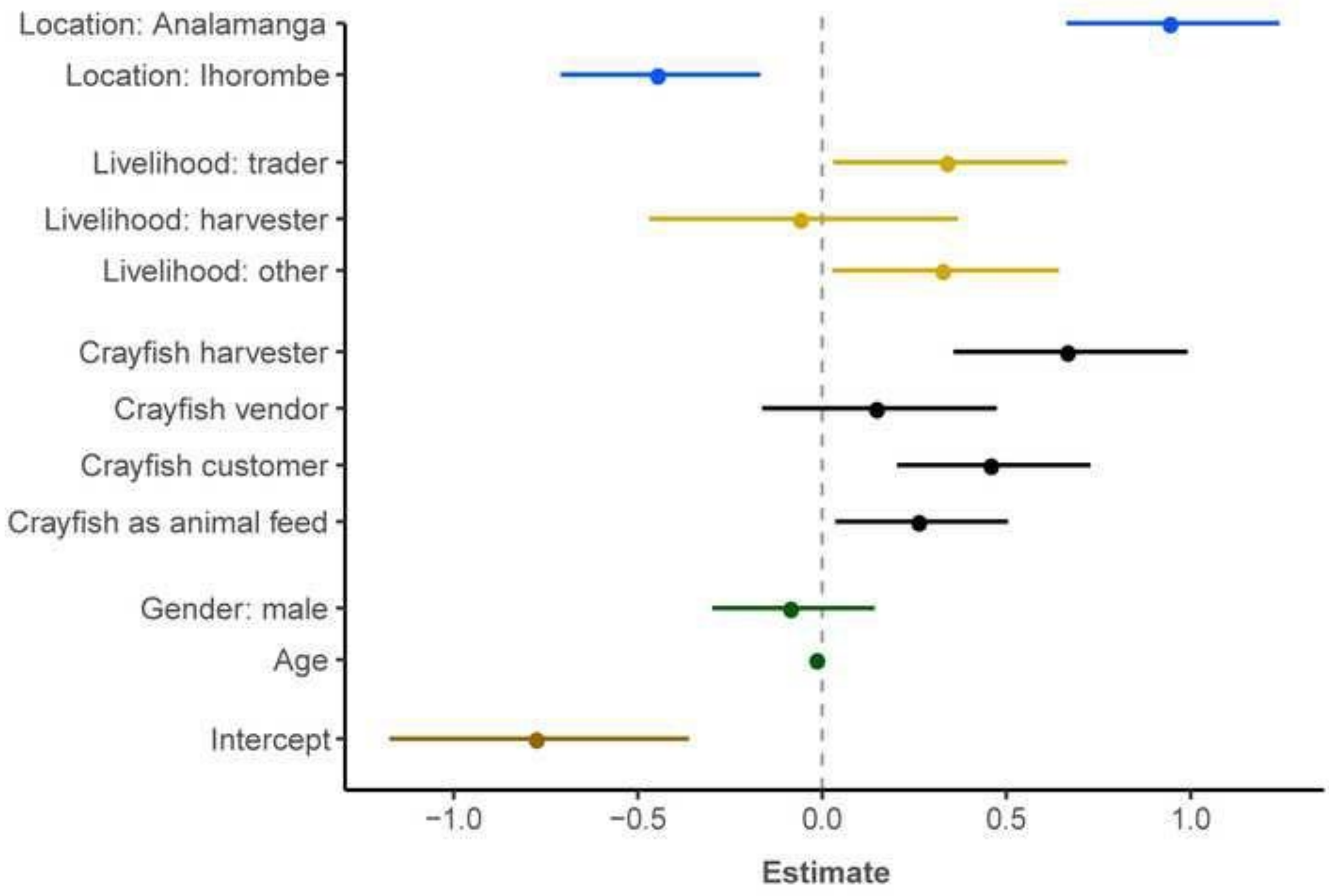

Figure 4

Click here to access/download;Figure;Figure 4.tif

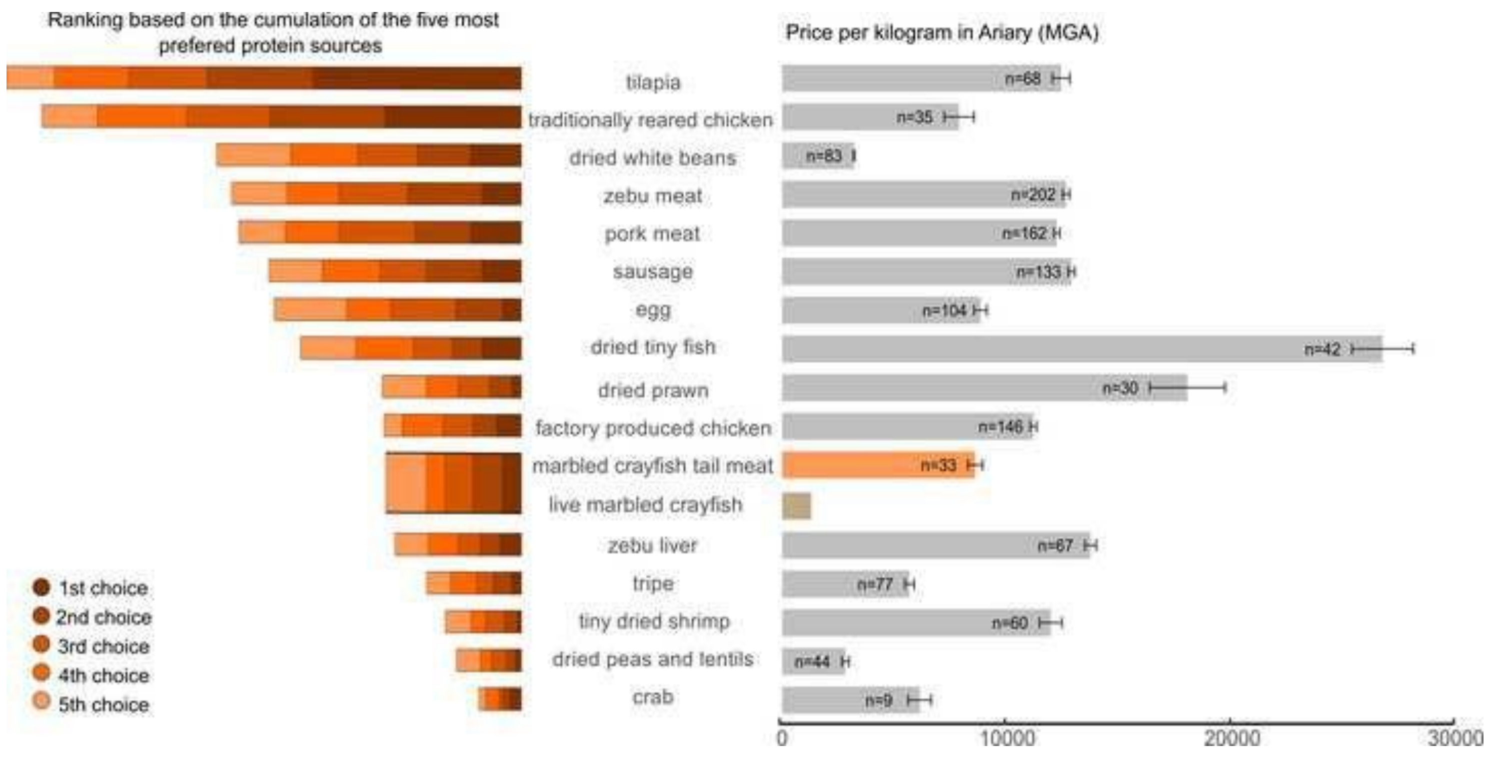

Figure 5

Click here to access/download;Figure;Figure 5.tif

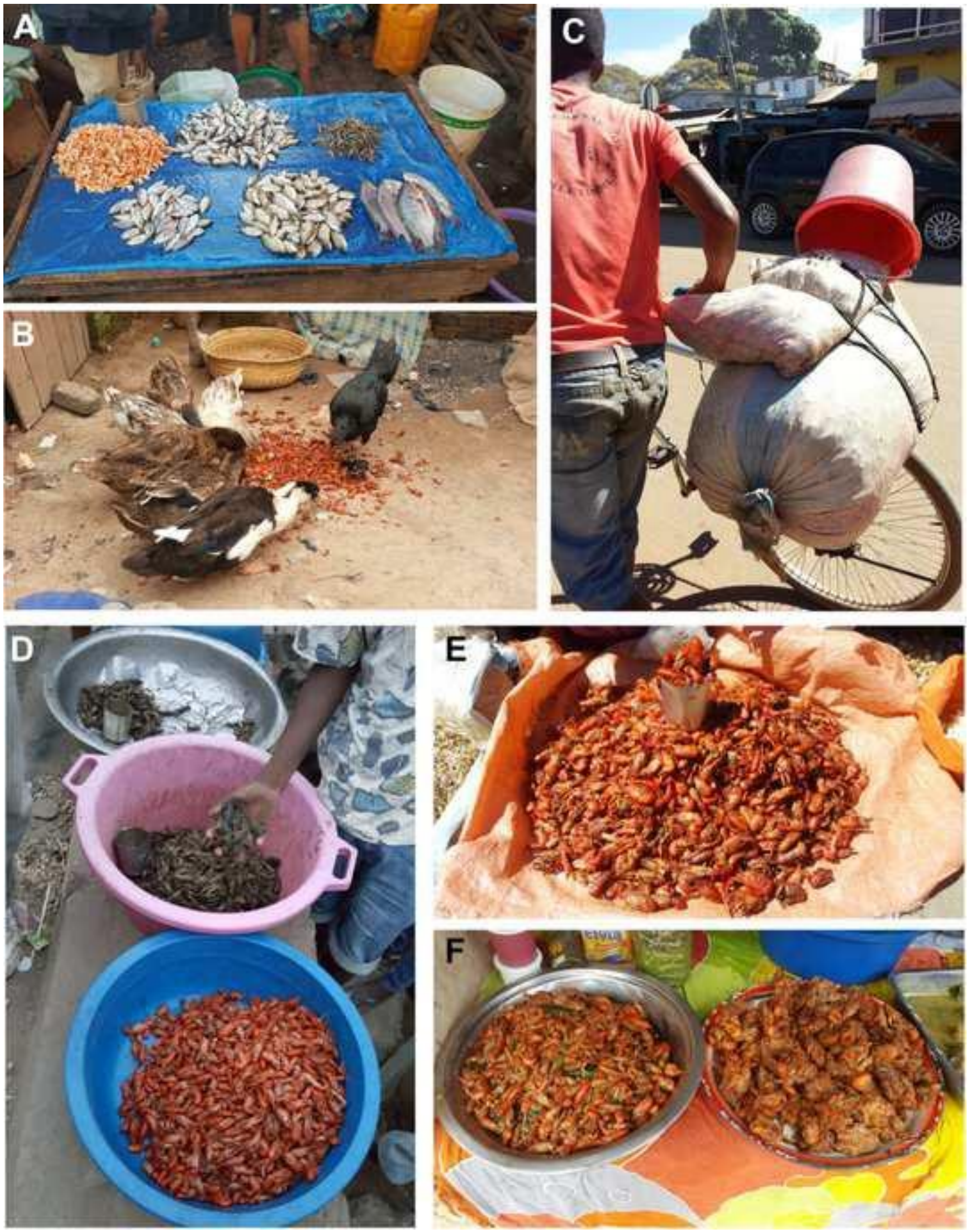

**Reviewer 6**
