# Supplementary material for: Perceived socio-economic impacts of the marbled crayfish invasion in Madagascar
Source: PLoS One. 2020 Apr 15;15(4):e0231773. doi: 10.1371/journal.pone.0231773 (PMC7159205; doi:10.1371/journal.pone.0231773)
Supplement: Attachment — Submitted filename: ms_submitted_ed2_comment_reviewers.docx [file pone.0231773.s004.docx]

**Socio-economic impacts of the marbled crayfish invasion in Madagascar**

Ranja Andriantsoa^1^, Julia P.G. Jones^2^, Vlad Achimescu^3^, Heriniaina Randrianarison^4^, Miary Raselimanana^4^, Manjary Andriantsitohaina^4^, Jeanne Rasamy^4^, Frank Lyko^1^

^1^Division of Epigenetics, DKFZ-ZMBH Alliance, German Cancer Research Center (DKFZ), 69120 Heidelberg, Germany; ^2^School of Environmental Sciences and Engineering, Bangor University, Bangor LL57 2UW, UK, ^3^School of Social Science, Mannheim University, 68159 Mannheim, Germany; ^4^Mention Zoologie et Biodiversité Animale, Université d’Antananarivo, BP906, 101 Antananarivo, Madagascar;

Corresponding author:	Frank Lyko

Deutsches Krebsforschungszentrum

Im Neuenheimer Feld 580

69120 Heidelberg, Germany

phone: +49-6221-423800

fax: +49-6221-423802

email: f.lyko@dkfz.de

Short title: Marbled crayfish impact in Madagascar

**Abstract**

**The negative environmental and economic impacts of many invasive species are well known. However, given the increased homogenization of the global biota, and the difficulty of eradicating species once established, a balanced approach to considering the impacts of invasive species is needed. The marbled crayfish (*Procambarus virginalis*) is parthenogenetic freshwater crayfish that was first observed in Madagascar around 2005 and has spread rapidly. We present the results of a socio-economic survey (n=385) in three regions of Madagascar that vary in when marbled crayfish first arrived. Respondents generally considered marbled crayfish to have a negative impact on rice agriculture and fishing, however the animals were also seen as making a positive contribution to household economy and food security. Regression modeling shows that those in regions with longer experience with marbled crayfish have more positive perceptions. Unsurprisingly, considering the perception that crayfish negatively impact rice agriculture, farmers have more negative views towards the crayfish than those involved in crayfish trading. Food preference ranking and market surveys reveal the acceptance of marbled crayfish as a source of cheap animal protein; a clear positive in a country with widespread malnutrition. While data on the biodiversity impacts of the marbled crayfish invasion in Madagascar is still completely lacking, this study provides insight into the socio-economic impacts of the dramatic spread of this unique invasive species in Madagascar.**

**“***Biby kely tsy fantam-piaviana, mahavelona fianakaviana*” (a small animal coming from who knows where which supports the needs of the family). Fokontany worker Analamanga, Madagascar.

**Introduction**

Invasive aliens species can have substantial negative effects on the environment, the economy and the human health [1–3]. Invasive species are major drivers of change in invaded ecosystems causing habitat modification, affecting biodiversity composition, and even sometimes resulting in extinction of species through direct predation or competition for food and space [4–6]. The damage caused by invasions and the management costs for the control can be a significant economic burden [7]. Also, invasive species can play important roles in disease transmission and spread [3]. While research has traditionally focused on these negative impacts, potential benefits from biological invasions can be overlooked. This may be because negative impacts are part of the definition of invasive species [8], but there is increasing awareness of the potential positive contributions made by invasive species [9–11]. Few studies have explored how perceptions of costs and benefits from an invasion may vary among people living with an invasive species and how this may change over time following an invasion.

Invasive crayfish have been recognized as a major threat to freshwater ecosystems. Indeed, their position in the food web (most are omnivorous) and also their capacity to transmit diseases can strongly impact an ecosystem. The rusty crayfish (*Faxionus rusticus*), for example, can significantly reduce resource availability in aquatic communities leading to biodiversity loss, while the signal crayfish (*Pacifastacus leniusculus*) transmits the crayfish plague in Europe which has resulted in extirpation of native crayfish from many locations [12,13]. Nonetheless, invasive crayfish species can also play a positive socio-economic role. *Procambarus clarkii* supports important commercial fisheries in many countries where it has been introduced [14,15], while the red claw crayfish (*Cherax quadricarinatus*) is an important income source for artisanal fishermen in Jamaica [16]. Studies have also suggested that invasive crayfish in Kenya play a role in control of schistosomiasis through predation on the snail hosts of this human parasite [17].

The marbled crayfish (*Procambarus virginalis*) originated from the German aquarium trade in the early 2000s [18,19]. Its ability to reproduce without males, as well as its high fecundity and resistance to harsh environmental conditions, predisposes it to be an invasive species [20,21]. Indeed, marbled crayfish have formed stable wild colonies in several European countries [22–26]. In Madagascar, marbled crayfish were first observed by biologists in markets around 2005 but were not identified until 2007 [21,27]. Initially confined to a small region around Antananarivo, their distribution area has now increased about 100-fold, covering eight out of 15 regions studied [28]. Conservationists quickly raised concerns [19,21] about potential impacts that marbled crayfish may have on endemic species, including 7 species of endemic crayfish in the genus *Astacoides* [29,30], should the invasive species overlap the native species range. In addition, there were also concerns that marbled crayfish may have a negative impact on rice agriculture and harvested fish populations. However from the start, marbled crayfish were found in markets and farmers were observed transporting the species intentionally around the island as early as 2007, suggesting that they were considered valuable [21]. More recently they have been observed as being widely sold in markets across Madagascar and being harvested in rice fields [31].

Our study aims to update information on the distribution of marbled crayfish in Madagascar and to explore attitudes towards marbled crayfish in three regions varying in how recently the marbled crayfish arrived. Specifically we explore perceptions of the positive or negative impacts on rice agriculture, fishing and its contribution to health, food security and household income. We also sought to understand more about the markets for this species, including the relative acceptance of marbled crayfish as a source of dietary protein. While information on the ecological impacts of the invasion of marbled crayfish in Madagascar is still entirely lacking, this paper provides valuable information on their socio-economic impacts.

**Materials and methods**

**Ethics statement**

Research involving human participants was approved by the Bangor University College of Environmental Sciences and Engineering research ethics committee (Approval Number: CoESE2019RAJPGJ01). Oral consent was obtained from each participant to voluntarily participate in the survey. Field research was approved by the Ministry of Environment and Forest of Antananarivo, Madagascar (research permits No. 58/19/MEDD/SG/DGF/DSAP/SCB.Re and No. 59/19/MEDD/SG/DGF/DSAP/SCB.Re).

**Update of marbled crayfish distribution**

Field work exploring the distribution of marbled crayfish distribution was conducted in March and April 2019 in three regions where marbled crayfish were not discovered in 2017 (Analanjirofo, Menabe, Atsimo Andrefana). For each region, we performed re-collection in each of our previous sampling stations [28] with additional new stations in the same area. We sampled the water bodies with traditional nets “tandroho” (50cm x 30 cm x 30cm) morning from 8:00 to 11:00 and from 15:00 to 18:00 for three days. Collections were performed by M.A.

**Social survey details**

The survey was carried out in March and April 2019 in three regions where marbled crayfish are present: Analamanga, Mahatsiatra Ambony and Ihorombe (see S1 Figure for details of interview locations in each region). In each region we first visited the regional authorities (Direction Regionale de l’Environnement, de l’Ecologie et des Forets, DREEF), as well as local authorities in each town or village where we worked (the commune or fokontany representative) to gain local permission for the work.

We collected three main types of data: (1) We carried out a brief questionnaire survey targeted at adults in the region designed to collect quantitative information. The questionnaire contains closed and Likert response questions. Sampling was opportunistic and we did not use a sampling frame. In each location, enumerators approached adults in markets, in fields, streets and villages, or working in and around water bodies. The questionnaire took approximately 15 minutes. (2) For participants who were identified as key informants (involved in selling crayfish, crayfish harvesting or having more knowledge on marbled crayfish), we asked additional open-ended questions. These in-depth surveys lasted 30-45 minutes. (3) Finally we also conducted a study of 30 markets (containing more than 200 stalls) in Antananarivo, where we recorded the price of processed marbled crayfish and other common sources of dietary protein as price per kilogram. XXX

For each respondent, we first clearly introduced ourselves and carefully explained the aim of our study and how data will be used. We explained that we are not taking any individually identifying information or recording voices. Due to the low level of literacy, oral consent was obtained from each respondent to voluntarily participate in the survey. Oral consent was also obtained for all photographs taken. Interviews were carried out by R.A., J.R., H.R. and M.R. with the help of trained enumerators. All interviewers were native speakers of Malagasy, comfortable in the dialects of the regions visited. Before starting an interview, each informant was shown a series of 16 photographs with crustacean and fish species to assess their knowledge of marbled crayfish (S2 Figure). Surveys were delivered using Open Data Kit (ODK)-Collect, an Android open source app for data collection. Data were submitted to ODK Aggregate when an internet signal was available.

We assessed the perception of marbled crayfish impacts by collecting information on the respondents' socio-economic status and their preferences. First, we assessed the preference among the most common sources of protein in local diets. We showed a series of 16 photographs (S3 Figure) illustrating different animal and plant based protein sources and asked the respondents to rank them in order of preference, before recording the first five. We then collected information on their primary and secondary livelihood (agriculture, government position, private monthly waged work, daily waged labor, trader, wild product harvester, other). We also recorded whether people own irrigated rice fields, and whether they fished for either income or subsistence or for recreation. We investigated the perception of marbled crayfish impacts on six different aspects (rice farming, fishing, household economy, food security, animal feed, health) using a Likert scale illustrated by Emojis (very bad, bad, neutral, good, very good). The full survey (in English and Malagasy) is available in the Supporting Information.

**Data analysis**

Data analysis was carried out using R3.4.3. Results from Likert scales were illustrated as diverging stacked bar charts using the *ggplot2* package. We also explored the relationships between marbled crayfish overall impact perception and potential predictors by fitting a multivariate ordinary least squares (OLS) regression. Our model investigated which factors predict whether the overall perception of marbled crayfish impact will tend to be more positive or negative. The dependent variable (the overall perception of marbled crayfish impacts) is measured on a 5-point ordinal scale: 1 being very positive and 5 very negative. Eight variables were considered as potential predictors: survey location, gender, age, livelihood, relationship to marbled crayfish (harvester, trader, buyer), and the use of marbled crayfish as animal feed. All variables except age are categorical and were transformed into dummy variables before running the regression.

For sensitivity analysis, we also ran an ordinal regression (function *polr* from R package MASS) with the same predictors, given the level of measurement of the dependent variable, and found similar results to the OLS regression (S1 Table). We present the results of OLS regression for ease of interpretability of coefficient and model fit. Finally, we evaluated the prediction model accuracy by running a k-cross validation analysis (10-fold) followed by a repeated k-cross-validation, using the functions *cv* and *repeatedcv* from the R package CARET, with a seed set on 523 for the reproducibility of the analysis (S2 Table).

**Results**

Compared to a previous survey from 2017 [28] we did not record an expansion of the known range in Madagascar during this survey (March and April 2019). Re-sampling at 18 previously negative sites in the Analanjirofo, Menabe and Vatovavy Fitovinany regions revealed no marbled crayfish (Fig. 1A). However, marbled crayfish were discovered at 15 additional sites in the Analamanga, Mahatsiatra Ambony and Ihorombe regions (i.e., within the previously identified range) during the present survey (Fig. 1A). The high density of marbled crayfish populations in these regions likely reflects the continuing anthropogenic distribution due to commercial exploitation [31].

We carried out social surveys in three regions (Fig. 1A): Analamanga (mostly in Antananarivo in 12 sampling points), Mahatsiatra Ambony (mostly in Fianarantsoa in 8 sampling points) and Ihorombe (mostly in Ihosy in 3 sampling points). We used haphazard sampling targeting the adult population, and approached 435 persons in total (Fig. 1B, S1 Figure). We obtained good participation rates (>86%) in all three regions (Fig.1B), with 142, 143 and 100 participants in Analamanga, Mahatsiatra Ambony and Ihorombe, respectively. The average participant age of 39 years, and a sex ratio of 54% (male) versus 46% (female). The most commonly reported primary livelihood among respondents was farmer (48%).

Fig. 1. Distribution of marbled crayfish in Madagascar. (A) Map of Madagascar. Light green dots indicate sites with known marbled crayfish populations from 2017 [28]. Dark green dots indicate additional sites where marbled crayfish were detected during this study (in 2019), light blue dots indicate 3 re-sampled negative sites from 2017 with no marbled crayfish. The three regions where the social surveys presented in this paper were carried out are highlighted by color. (B) Participant response rates and numbers of survey respondents per region. (C) Earliest observation of marbled crayfish in the three regions. Numbers indicate % respondents.

A subset of respondents (n=275) were asked when they first observed marbled crayfish in their local area. In the Analamanga region, a substantial fraction of respondents indicated that they became first aware of marbled crayfish before 2005 and the animals became well-known by 2010 (Fig. 1C). In contrast, marbled crayfish were mostly unknown in Mahatsiatra Ambony and completely unknown in Ihorombe before 2011 (Fig. 1C). This is consistent with a spread of the animals from the center to the south of Madagascar.

To explore the perception of marbled crayfish impacts, we used a Likert scale to investigate six variables. The results show that attitudes towards marbled crayfish vary substantially by region with respondents in the two southern regions (Mahasiatra Ambony and Ihorombe) having generally more negative attitudes towards the crayfish, while those in Antananarivo were generally more positive (Fig. 2). However, in all three regions, respondents reported some positive impacts of marbled crayfish and some negative impacts. Negative impacts on rice farming and fishing were reported at all sites (and by nearly 100% of respondents in Ihorombe), while contribution to food security and household economy was generally seen as positive.

Fig. 2. Perceived socio-economic impacts of marbled crayfish in 3 regions of Madagascar. Bar plots show overall impacts (Likert scales) in the three regions.

The responses to the open ended questions in the key informant interviews provided useful qualitative information to support and explain the quantitative data. The reasons for widely reported negative impacts of marbled crayfish on rice fields was due to burrowing activities which dry the rice fields and require the farmers to regularly repair their banks and irrigation canals. This results in more work for the farmer and can lead to disagreements between rice field owners. For example: "the marbled crayfish destroy the mud walls we build around our rice fields" (S4 Table, Q1-3). The occasional positive comments about the impacts of marbled crayfish on rice farming comes from the perception that marbled crayfish burrowing can improve rice production by loosening and aerating the soil (S4 Table, Q4). The perceived impacts of marbled crayfish on fishing were overwhelmingly negative, because the crayfish are known the predate on young fish, though not larger fish (S4 Table, Q5). Importantly, some impacts may not be directly observed after an initial marbled crayfish colonization: "the impacts on fishing can’t really be seen since the marbled crayfish are present for only two years" (S4 Table, Q6). Many people mentioned positive impacts from crayfish harvesting on local livelihoods from catching and selling crayfish, or using them as a cheap source of household protein or animal feed). A Fokontany worker in a village with several households depending on marbled crayfish described marbled crayfish as “a small animal coming from who knows where which supports the needs of the family” (S4 Table, Q7). Similarly, a farmer reported that marbled crayfish had given him a good living since they arrived in the area 10 years ago (S4 Table, Q8). Marbled crayfish were often mentioned as a good and cheap/free replacement for meat, and a source of energy and strength (S4 Table, Q9). However, some respondents have also observed allergic reactions, and stomach pain due to consumption of marbled crayfish (S4 Table, Q10).

In order to link the overall perception of marbled crayfish impact to socio-economic characteristics of respondents, we selected eight variables as predictors in a multivariate linear model. Our model explores the relationship between these eight variables and overall perception towards marbled crayfish (Fig. 3, S3 Table) show a good overall fit: the model successfully explains 44% of the variation of the dependent variable (adjusted R squared of 0.438). Additionally, the 10 fold cross-validation of our regression analysis revealed a good performance with an R-squared of 0.42. A positive overall attitude towards the marbled crayfish is associated with study site, as participants in Analamanga show a significant positive association (+0.95 scale points) and Ihosy a significant negative association (-0.44 scale points) compared to Fianarantsoa (controlling for gender, age and livelihood). Those involved in crayfish harvesting are more positive towards marbled crayfish than non-harvesters (+0.67 scale points). Also, the same picture was observed for crayfish customers and those using marbled crayfish as animal feed with respectively 0.46 and 0.27 scale points more positive compared to those who do not buy and use marbled crayfish. Finally, livelihood is significantly associated with the overall perception with traders (and our grouping of ‘other’ livelihoods) having a more positive average scores compared to farmers. This is perhaps unsurprising as farmers will suffer negative impacts associated with the damage marbled crayfish do to rice fields, while traders are more likely to benefit from selling crayfish.

Fig. 3. Coefficient plot of the model exploring the relationship between overall perception of crayfish and 8 socio-economic predictors. Blue bars represent the coefficients for locations while yellow bars shows the coefficients for livelihoods, black bars illustrate the coefficients crayfish-related activities and green bars the coefficient for demographic controls. Error bars represent 95% confidence intervals. Gender: male and Age were included as controls. The intercept was defined by the following parameters: location: Mahatsiatra Ambony; livelihood: farmer, not harvesting crayfish, not selling crayfish, not buying crayfish, not using crayfish as animal feed.

To assess the popularity of marbled crayfish in the diet of our survey participants, we asked them to identify and rank their five most preferred sources of nutritional proteins among 16 photographs of dietary protein sources provided (S3 Figure). The results showed that tilapia fish and chicken (sold live) represent the most preferred dietary protein (Fig. 4), which is consistent with previous findings [32,33]. Marbled crayfish meat was less preferred and grouped together with shrimps, factory produced chicken meat and beef liver (Fig. 4). Several other dietary protein sources, such as tripe, beans and crabs were less preferred than marbled crayfish.

Fig. 4. Preferences and relative price for kilo of a selection of common sources of dietary protein. Photographs and descriptions are provided in S2 Figure. Left panel: results of preference ranking for sources of dietary protein. Right panel: mean of market prices per kilo for each dietary protein source (n=number of stalls where prices were recorded in the 30 Antananarivo markets visited). Error bars represent 95% confidence intervals. For comparison, the price of live marbled crayfish from vendors in Ihorombe (1,200 MGA/kg) is also indicated.

In Antananarivo (Analamanga region), marbled crayfish were mainly sold in markets (Fig. 5A) and their leftover carapaces were used as animal feed (Fig. 5B). We found marbled crayfish in 33 stalls in 11 markets (out of 30 markets investigated). They were mainly sold as boiled tail meat, the average price per kg was 8,600 MGA (approximately 2 EUR, also see Fig. 3). This strongly contrasts with Ihosy (Ihorombe region), where marbled crayfish are predominantly sold live in large bags for widespread distribution (Fig. 5C). The marbled crayfish business is structured differently in Ihosy compared to Antananarivo: orders are received from different locations and regions. Traders then purchase live marbled crayfish from local collectors to respond to the market demand. Up to 20 bags of 35 to 80 kg with live marbled crayfish are transported daily with short and long distance "taxi-brousse" for a fixed fee per bag during the rainy season (November to March). The price of live marbled crayfish is much lower with an average of 1,200 MGA/kg (approximately 0.3 EUR).

Fig. 5. Commercial sales and distribution of marbled crayfish. (A) Marbled crayfish meat sold with freshwater fish. (B) Leftover shells from marbled crayfish used as animal feed. (C) Bag of live marbled crayfish for distribution. (D) Live and boiled marbled crayfish sold on the street. (E) Boiled and salted marbled crayfish sold in the market. (F) Ready-to-eat marbled crayfish sold in street food markets.

Interestingly, Fianarantsoa (Mahatsiatra Ambony region) was mentioned as one of the regions ordering from Ihosy. This was confirmed by interviews of marbled crayfish vendors in Fianarantsoa's evening market who indicated that they order marbled crayfish from Ihosy, and usually sell their complete order within a few hours (Fig. 5D). In Fianarantsoa, marbled crayfish were usually sold as processed food (Fig. 5E). Marbled crayfish beignets and meals with marbled crayfish in tomato and green onion sauce (Fig. 5F) were offered in several street food locations and street market restaurants. Interestingly, despite an official prohibition from the Ministry of Agriculture, Livestock and Fisheries (effective since 2009) that forbids the transport of live marbled crayfish, the transport of marbled crayfish (for example by taxi brousse), is supported by regular permits and fees. We interviewed taxi-brousse drivers from different transport companies in Ihorombe, who stated that those who transport marbled crayfish pay for a yearly permit, in the same way they need a permit to transport other freshwater products.

**Discussion**

It is now indisputable that we live in the Anthropocene; a geological age defined by humanity’s impact on the environment [34]. In this situation, some ecologists are rethinking their response to invasive species, increasingly recognizing that non-native species are a part of the ecology of the future [35,36]. While the strongly negative impacts of some invasive species render this a controversial view [37], those involved in managing invasive species have pointed out that benefits from such species are often overlooked or under-reported [36]. Our results, which provide the first information on the socio-economic impact of a parthenogenetic decapod described as ‘the perfect invader’ [21], provide a useful case study in how people make use of a new species in their environment. It also provides information useful for predicting the likely future spread of this invasive crayfish in a country with freshwater biodiversity of global value [38].

Madagascar is one of the poorest countries in the world, with 75% of the population living on less than $1.90 per day [39,40]. Poverty manifests itself in limited intake of dietary protein leading to stunting [41] which affects 1 in 2 Malagasy children [42]. In fact, many people rarely consume animal protein [32,33]. Given the lack of sufficient protein for human consumption; livestock are mostly fed rice bran, roots and tubers [43], providing insufficient protein for production. New and cheap sources of animal protein therefore have the potential to have a significant positive impact. Positive perceptions of marbled crayfish (especially prevalent in the Analamanga region) are strongly associated with harvesting, buying or using marbled crayfish for animal feeds. XXX The fact that the crayfish can be quite easily collected from public lakes, rivers, canals and rice fields means they can be collected at very low cost. Live animals are sold at low prices (live marbled crayfish are among the cheapest sources of animal protein available), which makes them affordable for poorer people. Leftover parts from processed marbled crayfish are fed to animals (mainly chicken and pigs); providing an additional source of income from those involved in the crayfish trade and providing a very effective, and relatively cheap, protein-rich livestock feed. In Antananarivo, where the species has been common for at least 10 years [21], the market has evolved and mostly peeled tail meat is sold. This value-added product (similar to processed fish and pre-cut vegetables) increases the profit available from the crayfish. A different market exists in Ihorombe, where the animals appeared considerably later and most trade is of live crayfish in bulk. While marbled crayfish are seldom among respondent’s most preferred food, they are ranked similarly to shrimps and factory processed chicken and thus represent an acceptable source of dietary protein.

The most significant negative impacts reported were on rice farming and fishing. A related species of crayfish (*Procambarus clarkii*) is known to negatively affect irrigated rice production in the Iberian peninsula, where it damages banks and irrigation canals by burrowing [44,45]. However, the extent to which rice production is impacted by marbled crayfish in Madagascar remains poorly understood and our survey results, and associated qualitative information, provided somewhat contradictory evidence. In Analamanga (where the crayfish have been present longest), the largest proportion of respondents reported no impact, while some reported a positive impact, perhaps because of the impact of the burrowing crayfish on soil aeration. It is possible that reported negative impacts of crayfish stem from the extensive media reporting of possible negative impacts on rice, rather than firsthand experience. The available evidence suggests negative impacts on fishing but further research is urgently needed to understand the impacts on fishing and freshwater ecosystems.

The overall perception of marbled crayfish impacts was significantly different in the three regions that were surveyed. Participants in the Analamanga region perceived crayfish more favorably than participants in the Mahatsiatra Ambony and Ihorombe regions. This may be due to differences in livelihoods of our sample population in the three regions. Those interviewed in the Mahatsiatra Ambony and Ihorombe regions rely more on farming, particularly rice farming, and perceived negative impacts on rice farming are likely to strongly influence the overall negative perception. Another possible explanation is that in Analamanga (where marbled crayfish have been established the longest; since about 2005) people have had more time to get used to the species’ presence and adapt to the opportunities it presents.

The geographical range of many invasive species is driven by socio-economic factors, such as trade, travel and the movement of goods [46,47]. Marbled crayfish were already sold in markets in Madagascar soon after they appeared. This raised the possibility that their potential economic importance could promote their spread [21]. Our work clearly confirms that the market demand for the crayfish is likely to encourage spread into new areas. Even in Ihorombe, where the overall perception of the crayfish is negative, the strong market demand and widespread distribution of live animals are likely to favor further spread.

It is also important to point out the limitations of our analysis. The opportunistic sampling approach used, and the relatively small numbers of interviews, means that our sample is not fully representative of the population in each region. Given the resources and time available, probabilistic sampling was not possible. However, we sampled several points in each region (see maps in S1 Figure) and the high response rate means that the responses are not likely to be biased. Furthermore, we controlled for differences in age and gender.

The spread of the marbled crayfish in Madagascar over the last decade has been nothing short of dramatic. Given the practical challenges to eradication of invasive species, a balanced approach to considering the costs of benefits arising from invasiveness is needed [36]. There are differing overall perceptions towards marbled crayfish both between regions and between people with different socio-economic characteristics. However it is clear that the market for marbled crayfish, and its widespread consumption, is likely to result in further spread. It is difficult to imagine any inhabitable parts of Madagascar that will remain uninvaded over the next decade. What this will mean for Madagascar’s freshwater biodiversity is still completely unknown. This study has attempted to provide some insight into what impact the invasion has on local people’s livelihoods. An important area of uncertainty remains the impact of marbled crayfish on rice agriculture. Given the importance of irrigated rice production to the economy of Madagascar, this is certainly worthy of further investigation. What this study has clearly demonstrated is that, since their appearance in Madagascar in the mid 2000s, marbled crayfish have rapidly become a widely consumed, cheap source of animal protein in local diets. Given the desperate need for protein in the diets of many Malagasy people, this can be considered a positive development.

**Acknowledgements**

We thank Maevatiana N. Ratsimbazafindranahaka and Luticia V. Raharimalala for their valuable support of our survey, and Amy Lewis for help with coding the survey in Open Data Kit. We also thank Soatiana Daniel and Laza S. Andriantsoa for their help with field trip management.

**References**

1.	McNeely JA, Mooney HA, Neville LE, Schei PJ W. A Global Strategy on Invasive Alien Species. IUCN, Gland, Switzerland and Cambridge, UK.; 2001.

2.	Pyšek P, Richardson DM. Invasive Species, Environmental Change and Management, and Health. Annu Rev Environ Resour. 2010;35: 25–55. doi:10.1146/annurev-environ-033009-095548

3.	Giuseppe M, Elena T. Invasive species and human health. Invasive species and human health. CAB International, Wallingford, UK.; 2018. doi:10.1079/9781786390981.0000

4.	Hejda M, Pyšek P, Jarošík V. Impact of invasive plants on the species richness, diversity and composition of invaded communities. J Ecol. 2009;97: 393–403. doi:10.1111/j.1365-2745.2009.01480.x

5.	Seeney A, Eastwood S, Pattison Z, Willby NJ, Bull CD. All change at the water’s edge: invasion by non-native riparian plants negatively impacts terrestrial invertebrates. Biol Invasions. 2019;21: 1933–1946. doi:10.1007/s10530-019-01947-5

6.	Clavero M, García-Berthou E. Invasive species are a leading cause of animal extinctions. Trends Ecol Evol. 2005;20: 110. doi:10.1016/j.tree.2005.01.003

7.	Hoffmann BD, Broadhurst LM. The economic cost of managing invasive species in Australia. NeoBiota. 2016;31: 1–18. doi:10.3897/neobiota.31.6960

8.	Bellard C, Genovesi P, Jeschke JM. Global patterns in threats to vertebrates by biological invasions. Proc R Soc B Biol Sci. 2016;283: 1–9. doi:10.1098/rspb.2015.2454

9.	Rogers P, Nunan F, Fentie AA. Reimagining invasions: The social and cultural impacts of Prosopis on pastoralists in southern Afar, Ethiopia. Pastoralism. 2017;7: 1–13. doi:10.1186/s13570-017-0094-0

10.	Ngorima A, Shackleton CM. Livelihood benefits and costs from an invasive alien tree (Acacia dealbata) to rural communities in the Eastern Cape, South Africa. J Environ Manage. 2019;229: 158–165. doi:10.1016/j.jenvman.2018.05.077

11.	Shackleton RT, Shackleton CM, Kull CA. The role of invasive alien species in shaping local livelihoods and human well-being: A review. J Environ Manage. 2019;229: 145–157. doi:10.1016/j.jenvman.2018.05.007

12.	Bobeldyk AM, Lamberti GA. A Decade after Invasion: Evaluating the Continuing Effects of Rusty Crayfish on a Michigan River. J Great Lakes Res. 2008;34: 265–275. doi:10.3394/0380-1330(2008)34[265:ADAIET]2.0.CO;2

13.	Jernelöv A. Crayfish Plague in Europe. The Long-Term fate of Invasive Species. Springer International Publishing; 2017. pp. 91–103. doi:10.1007/978-3-319-55396-2_7

14.	Food and Agriculture Organisation of the United Nations (FAO). Cultured aquatic fact sheets: Procambarus clarkii. 2009 [cited 6 Nov 2019]. Available: http://www.fao.org/fishery/culturedspecies/Procambarus_clarkii/en

15.	Wang Q, Ding H, Tao Z, Ma D. Crayfish (Procambarus clarkii) Cultivation in China: A Decade of Unprecedented Development. Aquaculture in China: Success Stories and Modern Trends. John Wiley & Sons Limited; 2018. pp. 363–377. doi:10.1002/9781119120759.ch4_1

16.	Pienkowski T, Williams S, McLaren K, Wilson B, Hockley N. Alien invasions and livelihoods: Economic benefits of invasive Australian Red Claw crayfish in Jamaica. Ecol Econ. 2015;112: 68–77. doi:10.1016/J.ECOLECON.2015.02.012

17.	Mkoji GM, Hofkin B V., Kuris AM, Stewart-Oaten A, Mungai BN, Kihara JH, et al. Impact of the crayfish Procambarus clarkii on Schistosoma haematobium transmission in Kenya. Am J Trop Med Hyg. 1999;61: 751–759. doi:10.4269/ajtmh.1999.61.751

18.	Lyko F. The marbled crayfish (Decapoda: Cambaridae) represents an independent new species. Zootaxa. 2017;4363: 544–552. doi:10.11646/zootaxa.4363.4.6

19.	Scholtz G, Braband A, Tolley L, Reimann A, Mittmann B, Lukhaup C, et al. Parthenogenesis in an outsider crayfish. Nature. 2003;421: 806. doi:10.1038/421806a

20.	Feria TP, Faulkes Z. Forecasting the distribution of Marmorkrebs, a parthenogenetic crayfish with high invasive potential, in Madagascar, Europe, and North America. Aquat Invasions. 2011;6: 55–67. doi:10.3391/ai.2011.6.1.07

21.	Jones JPG, Rasamy JR, Harvey A, Toon A, Oidtmann B, Randrianarison MH, et al. The perfect invader: A parthenogenic crayfish poses a new threat to Madagascar’s freshwater biodiversity. Biol Invasions. 2009;11: 1475–1482. doi:10.1007/s10530-008-9334-y

22.	Lokkös A, Müller T, Kovács K, Várkonyi L, Specziár A, Martin P. The alien, parthenogenetic marbled crayfish (Decapoda: Cambaridae) is entering Kis-Balaton (Hungary), one of Europe’s most important wetland biotopes. Knowl Manag Aquat Ecosyst. 2016;16: 1–9. doi:10.1051/kmae/2016003

23.	Patoka J, Buřič M, Kolář V, Bláha M, Petrtýl M, Franta P, et al. Predictions of marbled crayfish establishment in conurbations fulfilled: Evidences from the Czech Republic. Biologia (Bratisl). 2016;71: 1380–1385. doi:10.1515/biolog-2016-0164

24.	Pârvulescu L, Togor A, Lele S-F, Scheu S, Șinca D, Panteleit J. First established population of marbled crayfish Procambarus fallax (Hagen, 1870) f. virginalis (Decapoda, Cambaridae) in Romania. BioInvasions Rec. 2017;6: 357–362. doi:10.3391/bir.2017.6.4.09

25.	Deidun A, Sciberras A, Formosa J, Zava B, Insacco G, Corsini-Foka M, et al. Invasion by non-indigenous freshwater decapods of Malta and Sicily, central Mediterranean Sea. J Crustac Biol. 2018;076: 1–6. doi:10.1093/jcbiol/ruy076

26.	Lipták B, Mrugała A, Pekárik L, Mutkovič A, Grul’a D, Petrusek A, et al. Expansion of the marbled crayfish in Slovakia: Beginning of an invasion in the Danube catchment? J Limnol. 2016;75: 305–312. doi:10.4081/jlimnol.2016.1313

27.	Kawai T, Scholtz G, Morioka S, Ramanamandimby F, Lukhaup C, Hanamura Y. Parthenogenetic Alien Crayfish (Decapoda: Cambaridae) Spreading in Madagascar. J Crustac Biol. 2009;29: 562–567. doi:10.1651/08-3125.1

28.	Gutekunst J, Andriantsoa R, Falckenhayn C, Hanna K, Stein W, Rasamy J, et al. Clonal genome evolution and rapid invasive spread of the marbled crayfish. Nat Ecol Evol. 2018;2: 567–573. doi:10.1038/s41559-018-0467-9

29.	Jones JPG, Andriahajaina FB, Hockley NJ, Crandall KA, Ravoahangimalala OR. The ecology and conservation status of Madagascar’s endemic freshwater crayfish (Parastacidae; Astacoides). Freshw Biol. 2007;52: 1820–1833. doi:10.1111/j.1365-2427.2007.01766.x

30.	Boyko CB, Ravoahangimalala OR, Randriamasimanana D, Razafindrazaka TH. Astacoides hobbsi, a new crayfish (Crustacea: Decapoda: Parastacidae) from Madagascar. Zootaxa. 2005; 41–51. doi:10.11646/zootaxa.1091.1.3

31.	Andriantsoa R, Tönges S, Panteleit J, Theissinger K, Carneiro VC, Rasamy J, et al. Ecological plasticity and commercial impact of invasive marbled crayfish populations in Madagascar. BMC Ecol. 2019;19: 1–10. doi:10.1186/s12898-019-0224-1

32.	Jenkins RKB, Keane A, Rakotoarivelo AR, Rakotomboavonjy V, Randrianandrianina FH, Razafimanahaka HJ, et al. Analysis of patterns of bushmeat consumption reveals extensive exploitation of protected species in eastern madagascar. PLoS One. 2011;6: 12p. doi:10.1371/journal.pone.0027570

33.	Kurien J, López Ríos J. Flavouring Fish into Food Security. FAO SmartFish Publ. 2013.

34.	Lewis SL, Maslin MA. Defining the Anthropocene. Nature. 2015;519: 171–180. doi:10.1038/nature14258

35.	Carroll SP. Conciliation biology: The eco-evolutionary management of permanently invaded biotic systems. Evol Appl. 2011;4: 184–199. doi:10.1111/j.1752-4571.2010.00180.x

36.	Bonanno G. Alien species: to remove or not to remove? That is the question. Environ Sci Policy. 2016;59: 67–73. doi:https://doi.org/10.1016/j.envsci.2016.02.011

37.	Russell JC, Blackburn TM. The Rise of Invasive Species Denialism. Trends Ecol Evol. 2017;32: 3–6. doi:https://doi.org/10.1016/j.tree.2016.10.012

38.	Benstead JP, Rham PH De, Gattolliat J, Loiselle P V, Sartori M, Sparks JS, et al. Conserving Madagascar’s Freshwater Biodiversity. Sci York. 2009;53: 1101–1111. doi:10.1641/0006-3568(2003)053[1101:CMFB]2.0.CO;2

39.	World Bank; Macro Povery Outlook. Madagascar. 2016. Available: http://pubdocs.worldbank.org/en/615781477329260085/mpo-am16-mdg.pdf

40.	World Bank. Madagascar Country Overview. 2019 [cited 18 Sep 2019]. Available: https://www.worldbank.org/en/country/madagascar/overview

41.	Semba RD. The rise and fall of protein malnutrition in global health. Ann Nutr Metab. 2016;69: 79–88. doi:10.1159/000449175

42.	International Food Policy Research Centre. Global Hunger Index: Madagascar. 2018. Available: https://www.globalhungerindex.org/madagascar.html

43.	Food and Agriculture Organisation of the United Nations (FAO) and Livestock Information Sector Analysis and Policy Branch (AGAL). Madagascar Livestock sector brief. FAO. 2005.

44.	Anastácio PM, Marques JC. Crayfish, Procambarus clarkii, effects on initial stages of rice growth in the lower Mondego River valley (Portugal). Freshw Crayfish. 1997;11: 608–617.

45.	Anastácio PM, Soares M, Correia AM. Crayfish ( Procambarus clarkii ) consumption of wet-seeded rice plants ( Oryza sativa ): modifications throughout the rice growing period . SIL Proceedings, 1922-2010. 2005;29: 849–851. doi:10.1080/03680770.2005.11902800

46.	Tabak MA, Piaggio AJ, Miller RS, Sweitzer RA, Ernest HB. Anthropogenic factors predict movement of an invasive species. Ecosphere. 2017;8: 1–13. doi:10.1002/ecs2.1844

47.	Hulme PE. Invasion pathways at a crossroad: Policy and research challenges for managing alien species introductions. J Appl Ecol. 2015;52: 1418–1424. doi:10.1111/1365-2664.12470

**Supporting information captions**

S1 Figure. Map of survey locations.

S2 Figure. Pictures of crustaceans and fish for marbled crayfish identification.

S3 Figure. Pictures representing sources of dietary protein.

S1 Table. Statistical modelling of marbled crayfish perception.

S2 Table. Comparative analysis of linear and ordinal regression.

S3 Table. k-cross-validation analysis.

S4 Table. Quotes in English and in Malagasy languages.
