# Supplementary material for: Perceived socio-economic impacts of the marbled crayfish invasion in Madagascar
Source: PLoS One. 2020 Apr 15;15(4):e0231773. doi: 10.1371/journal.pone.0231773 (PMC7159205; doi:10.1371/journal.pone.0231773)
Supplement: Attachment — Submitted filename: Response to Reviewers.docx [file pone.0231773.s005.docx]

**Journal Requirements**

1. Please ensure that your manuscript meets PLOS ONE's style requirements, including those for file naming.

>> The manuscript was edited in order to meet the PLOS ONE’s style requirements.

2. In your Methods section, please provide additional location information of the sampling sites, including geographic coordinates for the data set if available.

>> Geographical coordinates are now provided in the tables S1-3.

**Additional Editor Comments**

1. Authors should pay particular attention to the detailed review submitted by Reviewer 1. They will significantly improve the structure and readability of the manuscript.

>> Reviewer 1 provided edits on the manuscript itself. We have read all of these suggested edits and thought about them carefully (including considering why the reviewer may have felt our wording could be better expressed). We have made some edits in response. However many we have rejected as we preferred our own way of expressing these points (or we have edited and found a clearer way in our own words).

2. Offer a test-statistic that can validate the use of OLS regression regarding the normal distribution (or not) of their dependent variable. If normality it rejected the results of their regression will be highly questionable.

>> In order to validate the use of OLS, we assessed the normality of the residuals. While the dependent variable shows a bimodal distribution (Fig. S5A), the distribution of the residuals is approximately normal (Fig. S5B). We also used a Q-Q plot in order to check the assumption. The linearity of the points satisfies the assumption of normal distribution (Fig. S5C). Finally, we performed a Shapiro-Wilk normality test on the residuals which revealed a p-value=0.603 (>0.05); therefore the null-hypothesis of normal distribution cannot be rejected. These results have now been integrated into the manuscript.

3. The authors should also include p-values from t-tests regarding whether mean values and coefficients, respectively, are different from 0. These should be added to Figures 2 and 3.

>> Results in Figure 2 are descriptive. Therefore, no statistical test was performed on the data set. For Figure 3, the p-values are provided in Table 1. This is now clarified in the figure legend.

4. Details should be disclosed on their type of ordinal regression (e.g. ordinal logit/probit, tobit, other?). These model specifications all offer p-values for each coefficient. None are presented in the Appendix.

>> The ordinal regression we used is a proportional odds logistic regression (Agresti et al., 2002; Venables et al., 2002). This information was now added to the method section. The p-values for each coefficient for the ordinal regression are now provided in Table S4.

5. Results from their main regression model should be reported within the main body of the paper. This is a major result that should not be placed within Appendices.

>> The regression table has been moved to the main body of the paper.

6. Typo in S3 table (repeated) should be corrected.

>> The typo in Table S3 (now Table S5) was corrected.

**Reviewer 1**

1. The manuscript is plagued with improper English and needs considerable attention to detail before it is acceptable for publication (i.e., redundancies, contradictions, verb tense, splitting verbs, misspellings, and run-on sentences). I made many edits throughout the paper (see PDF) for consideration in the next round. For example, why is it important to mention that a Likert scale was used 5 times? What is a substantial fraction (did you mean percent)? Why did you say, tilapia fish? The word ‘order’ is used twice in the same sentence; once as a noun and once as a verb.

>>The manuscript was carefully edited after reading the comments and suggestions. However we have not accepted the edits of our text when we felt these changed the style or voice in ways we didn’t feel comfortable with.

2. Terminology, in some cases, was inconsistent with traditional usage in the social sciences. For example, it was a convenience sample, not haphazard or opportunistic.

>> We have changed terminology in some places in response to these comments.

3. Although the sampling period was identified (March and April 2019), it did not include information on weekday vs. weekend data collection.

>> Data was not exclusively collected on weekend of weekdays We have clarified this point.

4. The perception of marbled crayfish was measured by 6 items (not aspects), yet a Cronbach’s alpha (reliability coefficient) was not provided.

>> While performing the data analysis, we did not create a sum score index and only considered the items separately; therefore, a Cronbach’s was not appropriate. For this reason, this information was not added to the manuscript.

5. The dependent variable (perception of impacts) was measured on a 5-point scale and referred to as ordinal data. Most social science research uses a 5-point scale and calls it interval data. Your choice of statistics hinges upon this decision. Please re-run your data using linear regression to see how the results compare with OR and OLS. Perhaps you can improve R2 even more than it is already.

>> In our analysis, we treated the 5 point-scale as interval data, and we checked for the residuals which are normally distributed. We used ordinal regression for sensitivity analysis. The results of the Ordinal Regression (OR) and Linear Regression (Ordinary Least Square) models are also comparable: coefficients that are positive in OLS are also positive in OR, same for the negative coefficients, and those not significantly different from 0 (p>0.05). This is described in Table S4.

6. I did not see a sample size of key informants, or a description on how you analyzed this data. I saw some direct quotes in the narrative, but it would be better if you had categorized the open-ended information so I could see it in a table.

> We treated the whole data set together, as the same closed-ended questionnaire was used for all participants (see appendices which provides copies of the survey instruments). The only difference with the key informants is that they were asked additional open-ended questions. We used the quotes from these open-ended questions (52 participants out of 351) to complement our quantitative findings. We did not perform a separate analysis for the open-ended questions as the data set was small, particularly when split per region. We have added information on sample size to the results section.

**Reviewer 2**

We thank the reviewer for his/her attention to detail. The manuscript was carefully edited after reading the comments and suggestions. We provide three new tables to the supplementary information S1-3 in order to clarify the study locations and sampling details as suggested by this reviewer. Most comments were made on the manuscript. We respond to these below:

The reviewer didn’t like the use of the quote to open the manuscript.

>> This is commonly done in social sciences papers. Also, all authors really like the use of this special quote which captures the essence of the paper and places local people’s voices at the heart of the paper. The other reviewers and editor didn’t ask us to remove it and we would very much prefer to keep it.

“What about people who did not like it? - you seem only get information for those who like the marbled crayfish but no mention of why others hate to eat it ?? so probably best to mention that the investigation was limited to some type of people in the region.”

>> A detailed explanation of our sampling approach is provided in the manuscript. While it is not perfect, we have interviewed a wide range of people with different, region-specific perceptions.

**Reviewer 3**

The authors should mention the social aspect of marbled crayfish in the Analamanga region where mostly only poor people rely on it as source of protein and many others have some "pejorative consideration" of this species, which means a kind of "seeing people eaten it as very poor" --- such consideration is not linked with its impact to the biodiversity but some type of "social and cultural" consideration locally known as "foza orana" : something bad - destroying etc. This mentality might have impact on limiting its consumption in the Analamanga region

>> We thank the reviewer for this constructive comment. The negative associations that many people have for the marbled crayfish as “foza orana” is indeed interesting and we have added this to the text.
