# Supplementary material for: Perceived socio-economic impacts of the marbled crayfish invasion in Madagascar
Source: PLoS One. 2020 Apr 15;15(4):e0231773. doi: 10.1371/journal.pone.0231773 (PMC7159205; doi:10.1371/journal.pone.0231773)
Supplement: Attachment — Submitted filename: Response to reviewers 2.docx [file pone.0231773.s007.docx]

**Editor comments**

1. Reviewer 1, who previously provided a very comprehensive review, deems some of his comments have not been fully addressed.

>> We have now fully addressed the points that were raised by Reviewer 1.

2. The title shall be updated to reflect the clear nature of the study. This research examined 'perceived' impacts. It did not assess impacts per se, but the perceptions of those afflicted by this invasive species. For instance, it could be updated to "Perceived socio-economic impacts of the marbled crayfish invasion in Madagascar"

>> Done as suggested.

1. Reviewer 1 is also clear on the need for a complete proof-read of the manuscript and has provided additional comments as an Appendix. The authors shall proof-read the manuscript for the proper use of English. As a case in point, the last sentence in the Abstract illustrates several grammatical issues (e.g. data are plural) "While data on the biodiversity impacts of the marbled crayfish invasion in Madagascar is still completely lacking, this study provides insight into the socio-economic impacts of the dramatic spread of this unique invasive species in Madagascar."

>> We have accepted as many of their suggested edits as possible. However, we do somewhat object to the implication that our English was not of high quality and we are holding out on some suggested edits. Please also see our response to Reviewer 1 below and the cover letter by Julia Jones.

**Reviewer 1**

1. “Despite having "carefully edited" the manuscript, the authors still need to address a number of grammatical problems / issues before it can be published. I have addressed many of them (see the copy edits). Although some of my suggestions are stylistic (subjective), others are objective (not subject to what you feel comfortable with). For example, splitting the verb (have recently been observed) is improper English. It's a problem throughout your manuscript.”

>> The reviewer is understandably frustrated that (s)he spent a lot of time editing our manuscript and we did not accept the edits. After the first round of reviews, the author team decided not to accept many of the edits suggested by this reviewer because the 2^nd^ author (a native speaker) did not agree with this reviewer’s stylistic edits and argued to retain many of our formulations. Perhaps we should have explained this decision more clearly in our response after the 1^st^ round. This time, as the reviewer is insisting on the edits and the editor has supported them, we have accepted the majority of the edits (more than 130 out of the 144 edits that were made). However, we have rejected some edits that would have changed the meaning or the emphasis of the contents of our manuscript. For example, the reviewer wanted us to change ‘primary livelihood’ for ‘primary job’. This is not appropriate as in most cases local livelihood’s do not depend on a ‘job’ as usually understood. We have also rejected the edits to the first paragraph of the discussion.

2. “The second issue involves the dependent variable. Social science does not support the use of one "global" indicator for overall perception. You measured six items, and they should be summed to form an overall perception. That's the purpose of the scale, hence a Cronbach's alpha is a needed and appropriate statistic to include in your analysis.”

>> The reviewer is suggesting that the various items in our survey are measuring a latent variable which relates to a general attitude towards the crayfish. We have redone the analysis in the way the reviewer suggests. Reassuringly, the results confirmed our previous conclusions (as detailed below). The manuscript has been revised and updated correspondingly.

We changed the dependent variable from a one-item overall perception of crayfish impact to an index composed of 4 items: overall perception, impact on household economy, impact on food security, impact on health. We did not include the other impact variables (rice farming, animal feed and fishing), because they were not presented to the full sample, but only shown to respondents engaged in these activities. We wanted to keep the balanced sample that includes people with different livelihoods. Because there was missing data due to nonresponse, we imputed scale values for some cases, but only for the respondents with at least 2 of 4 valid (non-missing) item responses. We checked the 4-item scale reliability using Cronbach’s alpha, which showed a satisfying level of internal consistency. Finally, the index was standardized to a mean of 0 and standard deviation of 1 to simplify the interpretation of the regression coefficients.

We then ran the linear regression (OLS) with the new dependent variable (crayfish impact index), and checked the residuals for violations of assumptions (there were none). We removed the ordinal regression sensitivity check, since it was not adequate anymore (the dependent variable measurement level can be treated as interva ldata). The results were very similar to the ones where we used the overall perception index.
